# Meroterpene-Like α-Glucosidase Inhibitors Based on Biomimetic Reactions Starting from β-Caryophyllene

**DOI:** 10.3390/molecules25020260

**Published:** 2020-01-08

**Authors:** Da-Wei Yan, Cheng-Di Huang, Hang-Hang Zheng, Na Zhao, Xiao-Lan Feng, Shuang-Jiang Ma, An-Ling Zhang, Qiang Zhang

**Affiliations:** 1 Shaanxi Key Laboratory of Natural Products & Chemical Biology, College of Chemistry & Pharmacy, Northwest A&F University, Yangling 712100, China; 15829769577@163.com (D.-W.Y.); chengdihuang@nwafu.edu.cn (C.-D.H.); zhh18710683097@163.com (H.-H.Z.); 1984162639@nwafu.edu.cn (N.Z.); tianjijuechen00@163.com (S.-J.M.); 2 College of Veterinary Medicine, Northwest A&F University, Yangling 712100, China; xiaolanfeng@nwafu.edu.cn

**Keywords:** meroterpene, 4-hydroxycoumarin, β-caryophyllene, lawsone, hetero[4+2] cycloaddition, α-glucosidase, hyperglycemia

## Abstract

*Background*: Natural meroterpenes derived from phloroglucinols and β-caryophyllene have shown high inhibitory activity against α-glucosidase or cancer cells, however, the chemical diversity of this type of skeletons in Nature is limited. *Methods*: To expand the chemical space and explore their inhibitory activities against α-glucosidase (EC 3.2.1.20), we employed β-caryophyllene and some natural moieties (4-hydroxycoumarins, lawsone or syncarpic acid) to synthesize new types of meroterpene-like skeletons. All the products (including side products) were isolated and characterized by NMR, HR-MS, and ECD. *Results*: In total, 17 products (representing seven scaffolds) were generated through a one-pot procedure. Most products (12 compounds) showed more potential activity (IC_50_ < 25 μM) than the positive controls (acarbose and genistein, IC_50_ 58.19, and 54.74 μM, respectively). Compound **7** exhibited the most potent inhibition of α-glucosidase (IC_50_ 3.56 μM) in a mixed-type manner. The CD analysis indicated that compound **7** could bind to α-glucosidase and influence the enzyme’s secondary structure. *Conclusions*: Compound **7** could serve as a new type of template compound to develop α-glucosidase inhibitors. Full investigation of a biomimic reaction can be used as a concise strategy to explore diverse natural-like skeletons and search for novel lead compounds.

## 1. Introduction

Natural products (NPs) are a diverse family of organic molecules, most of which exhibit significant bioactivities to treat human diseases [1,2,3,4,5,6,7,8]. Thus NPs are an essential source for discovering novel drugs or lead compounds [6]. However, the range of scaffolds readily accessible in Nature is limited [9]. Furthermore, interest in generating novel synthetic scaffolds has recently declined in pharmaceutical research due to the elaborate isolation procedures required or lengthy total synthesis pathways [9]. Recently, there has been increased interest in NP-inspired or NP-like products to improve the chemical diversity of NPs through different routes: (1) diversity-oriented synthesis (DOS) starting from isolated NPs [10,11], (2) expansion of NP space catalysed by uncommon P450 reactions [12], (3) construction of new lead compounds inspired by bioactive NPs [13], (4) recombined NP moieties generated via coupling reactions [14], (5) diversity-enhanced extraction directly from plants [15]. In particular easily accessible NPs can serve as ideal starting scaffolds to efficiently afford diverse NP-like structures [16]. This strategy can avoid lengthy reaction procedures for building complex skeletons.

**Scheme 1:**
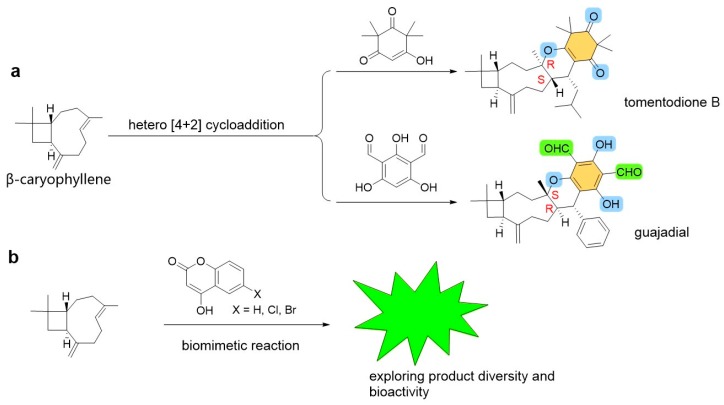
(**a**) Typical meroterpenes and their biogeneration in Nature. (**b**) A biomimetic strategy to explore NP-like meroterpenes.

Natural meroterpenes, arising from phloroglucinols, have attracted recent interest in natural products chemistry, total synthesis, and medicinal chemistry due to their special skeletons and bioactivities [17,18,19,20]. Especially the typical skeletons obtained from guava (*Psidium guajava*) and *Rhodomyrtus tomentosa* [21,22,23,24,25,26,27,28] contain a phloroglucinol moiety and a sesquiterpene skeleton. They are biogenerated through a hetero [4+2] cycloaddition (Scheme 1a). Some of them possess high activities against cancer cells and α-glucosidase. However, only very limited skeletons were found in Nature so far. In our previous work, some phloroglucinol-like moieties such as lawsone, 2,4,6-trihydroxyisophthalaldehyde [29], and polyphenol moieties [30] could promote *α*-glucosidase inhibition. The NP 4-hydroxycoumarin is considered as a moiety responsible for *α*-glucosidase inhibition [31]. Also, the compound can be applied to construct various products due to its reactivity [32]. We thus employed 4-hydroxycoumarin and its halogenated derivatives to explore new meroterpene-like products using the biomimetic reactions (Scheme 1b).

## 2. Results and Discussion

### 2.1. Synthesis

**Scheme 2:**
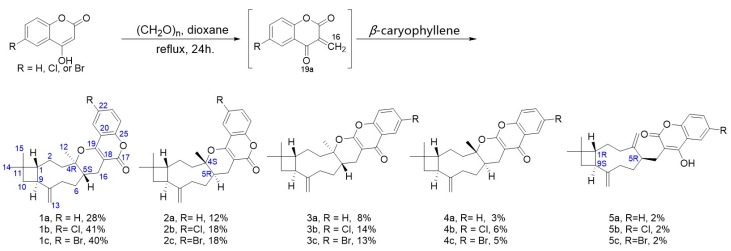
One-pot synthesis of meroterpene-like compounds starting from caryophyllene and 4-hydroxycoumarins. *Reagents and conditions*: (CH_2_O)_n_, anhydrous dioxane, reflux, 24 h.

**Scheme 3:**
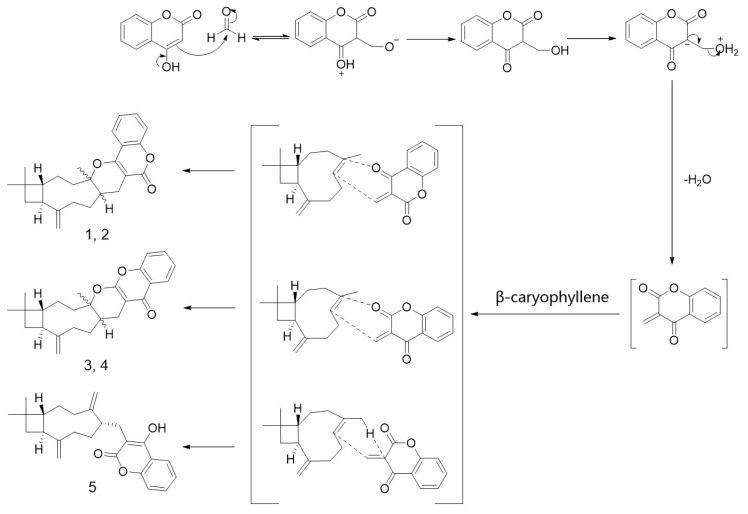
A speculated mechanism for the formation of the skeletons **1**–**5**.

The synthesis was carried out by a one-pot procedure from coumarin, paraformaldehyde, and *β*-caryophyllene. In total, 15 compounds corresponding to five different skeletons (Scheme 2) were acquired. Due to the electron donation of O-19a in the coumarin building blocks, the major products were obtained from cycloaddition at the 16 and 19a positions (Scheme 2). The product diversity can be rationalized by the reaction mechanism shown in Scheme 3. The reaction starting from the unsubstituted 4-hydroxycoumarin has already been reported [33,34]. However, the reported procedure did not separate the isomer pairs of **1a**/**2a** and **3a**/**4a** since their chromatographic retention times are very close. We optimized the separation to purify these isomers. In the presence of Cl- or Br- substituents on the 4-hydroxycoumarin moiety, the total conversion increased to 81% or 78%, respectively. Generally, the stable conformers of caryophyllene favored generating (4*R*,5*S*)-configuration products. However, in the reported total synthesis [35], it was still tricky to obtain enantioselective products. We did not optimize the reaction to promote geneartion of a single target product since the purpose of the reaction in the current work was to acquire diverse skeletons and screen for bioactive products. Thus we explored all the major and side products to get diverse compounds to seek higher bioactivities.

**Figure 1:**
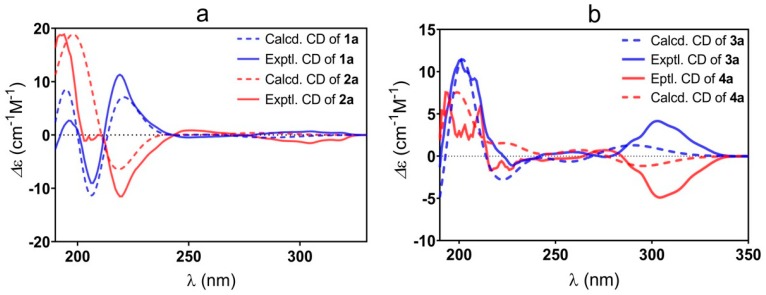
Experimental and calculated ECD spectra of **1a** and **2a** (**a**), **3a**, and **4a** (**b**).

**Figure 2:**
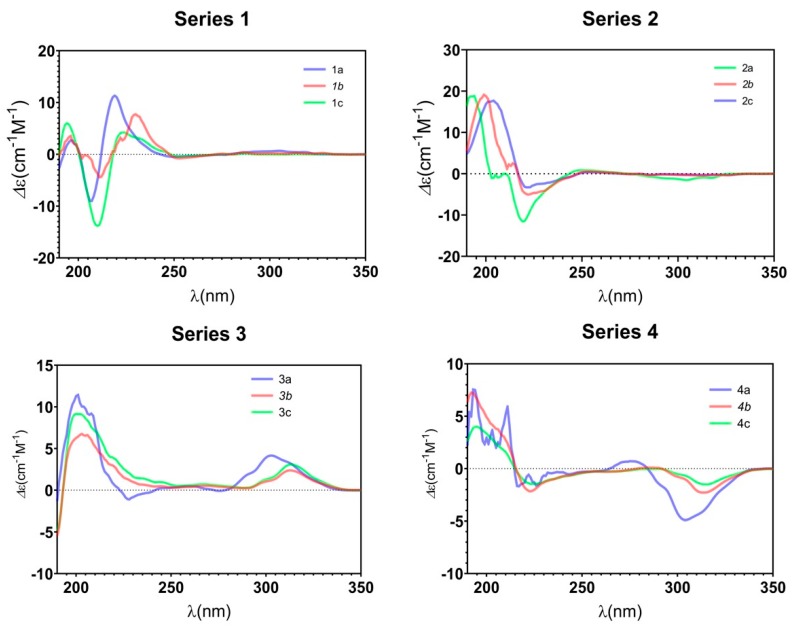
Experimental ECD spectra of compound series **1**–**4**.

To assign the absolute configurations (ACs) of those newly formed chiral carbons (C-4 and C-5) unambiguously, we carried out ECD calculations [36,37] and compared the calculated curves with the experimental ones. The skeletons **1** and **2** possess the same planar structures but inverse chiralities at C-4 and C-5. Their experimental ECD spectra showed an almost mirror curve from 212 to 320 nm. Comparing their experimental ECD spectra with the calculated curves of the (4*S*,5*R*) and (4*R*,5*S*)-isomers allowed us to establish the absolute configurations of **1a** and **2a** (Figure 1a). The ACs of **3a** and **4a** were also determined by ECD associated with the calculated spectra (Figure 1b). The halogen (Cl or Br)-substituted products **b** and **c** in the series **1**–**4** had the same Cotton effect as the corresponding **a** products, respectively (Figure 2), which indicated the same configurations of C-4 and C-5 in each set of compounds **1**–**4**.

**Figure 3:**
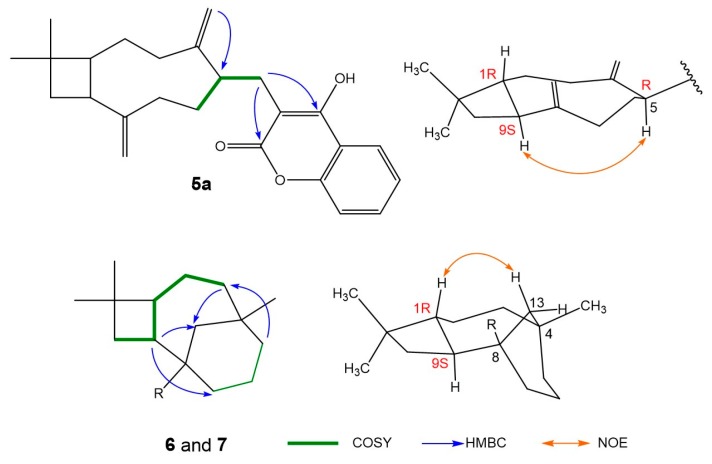
Selected 2D NMR correlations of compounds **5a**, **6**, and **7**.

The planar structure of **5a** was confirmed by the HMBC correlations, as shown in Figure 3. The chiral C-5 is connected to the chromophore via a flexible CH_2_ group, thus its configuration cannot be determined from the ECD spectra. However, the chirality of C-1 and C-9 is inherited from *β*-caryophyllene and remains unchanged during the synthesis procedure. Thus, the NOESY correlation from H-9S to H-5 (as shown in Figure 2) indicated the chiral C-5 was in an (*R*) configuration. The corresponding ^1^H- and ^13^C-NMR data of **5b** and **5c** when compared with those of **5a** indicated that the compounds **5b** and **5c** possess the same scaffolds and configuration as **5a**.

**Scheme 4:**
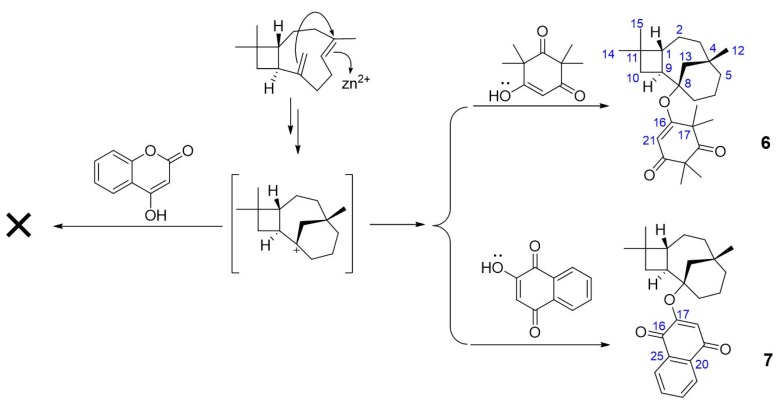
Meroterpene-like compounds coupling caryophyllene with lawsone or syncarpic acid. *Reagents and conditions*: ZnI_2_, toluene, 110 °C, 18 h.

Inspired by the high inhibition against α-glucosidase exhibited by compound **5a** (Table 1), we considered that a flexible connection between coumarins and caryophyllene would be beneficial for the bioactivities. To verify our speculation, we attempted to connect 4-hydroxycoumarin and the caryophyllene skeleton via an ether bond. However, according to the GC-EIMS and LC-QTOF-MS/MS analysis of the crude product, none of the expected MS signals were detected (see Figure S1 in Supplementary Materials). Then we used another two similar building blocks (syncarpic acid and lawsone) to replace the 4-hydroxycoumarin to acquire products **6** and **7** (Scheme 4). These two building blocks can also provide hydrogen binding sites. In the presence of ZnI_2_, the skeleton of caryophyllene rearranged via a transannular reaction to yield a relatively stable carbocation, which was then attached to the hydroxyl to afford compounds **6** and **7**.

The planar structures of **6** and **7** were elucidated according to the 2D NMR correlations, as shown in Figure 3. Since the chirality of C-1(*R*) and C-9(*S*) is already known, the newly formed chiral centers (C-4 and C-8) in compounds **6** and **7** were deduced according to the NOESY correlation H-1/H-13 as shown in Figure 3.

### 2.2. α-Glucosidase Inhibition

*α*-Glucosidase is a key enzyme for hydrolyzing amylum into glucose, which will raise the glucose levels in the blood [38]. Thus inhibiting this enzyme will postpone glucose absorption, thus lowering postprandial blood glucose levels [39]. To screen for lead compounds, we primarily bioassayed all the compounds and building blocks on *α*-glucosidase at the 25 and 50 μM level. Those compounds (as listed in Table 1) that showed high inhibition (>50% at 25 μM level) were then subjected to further IC_50_ evaluation. Compounds **5a**, **6,** and **7** showed promising inhibition (IC_50_ < 10 μM).

Interestingly, none of the building blocks, including the coumarins and caryophyllene, showed any inhibitory effects against α-glucosidase. Only when the coumarins were combined with caryophyllene to form meroterpene-like compounds, inhibition was observed. The compound set **1** (**1a**, **1b,** and **1c**) all showed weak activities against the bio-target. The compound sets **2**–**4** showed moderate inhibition against the target protein. The halogen atoms might provide an additional site for hydrogen bonding since compounds **2a**, **3a** and **4a** showed limited inhibition at 25 μM while the compounds **2b** and **3b** (Cl substituted) and **2c** and **3c** (Br substituted) all showed moderate activities (IC_50_ 12.0–23.6 μM).

The compound set **5** showed better inhibition than compounds **2**–**4**. These results indicated that a flexible connection between *β*-caryophyllene and 4-hydroxycoumarin is beneficial to improve the inhibition. Furthermore, the hydroxyl group could be necessary for binding to the target protein. When the rearranged caryophyllene was linked with aromatic ketones through an oxygen bridge (compounds **6** and **7**), the product showed better inhibition than the positive control. Among our synthetic products, compound **7** showed the highest inhibition, probably due to the presence of the *para*-substituted carbonyls and the hydrophobic caryophyllene moiety.

**Figure 4:**
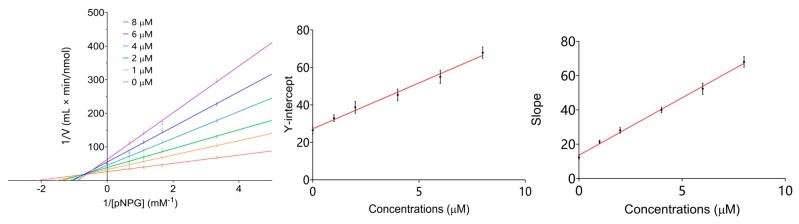
Lineweaver-Burk plots of compound **7.**

To explore the interaction mechanism of the best inhibitor **7**, we carried out the enzyme kinetic study using Lineweaver-Burk plots analysis [40,41]. *α*-Glucosidase was treated with pNPG and compound **7** at various concentrations. As shown in Figure 4, compound **7** showed a non-competitive type of inhibition against *α*-glucosidase. Replotting the slope and Y-intercept values taken from each line in the primary Lineweaver–Burk plot allowed extrapolation of the inhibition constants K_i,free_ (the affinity to the free enzyme) and K_i,bound_ (the affinity to the complex enzyme-substrate). The K_i,free_ and K_i,bound_ values of **7** were 2.03 and 5.57 μM, respectively.

**Figure 5:**
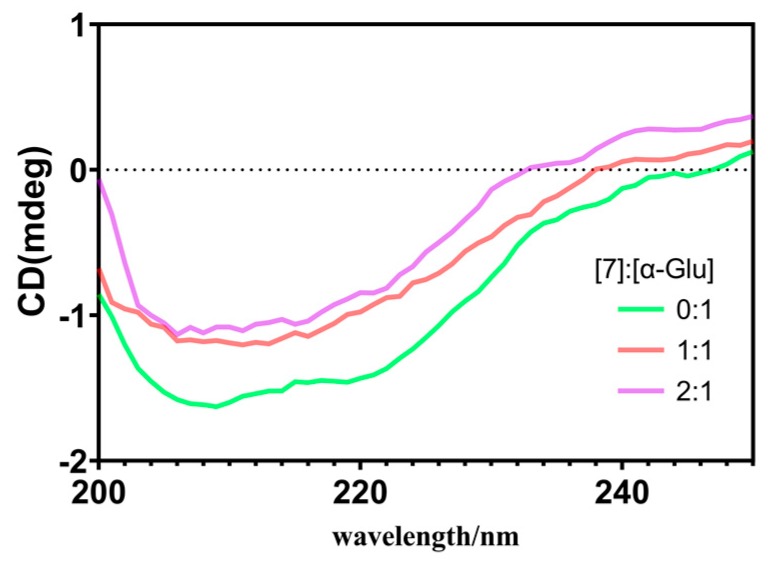
CD spectra of α-glucosidase in the presence of compound **7**. The concentration of α-glucosidase is 2 μM. The molar ratios of compound **7** to the enzyme were 0:1, 1:1, and 2:1.

CD spectroscopy is considered as a reliable and sensitive method for monitoring the secondary structural changes of macromolecules interacting with small molecules [42]. The *α*-glucosidase yield two negative bands at 209 and 219 nm on CD the spectrum which originated from the *n* π* and π ⟶ π* electron transfer of the *α*-helix motifs [43,44]. With increasing levels of compound **7** to the enzyme, a regular increasing intensity of both negative bands occurred (as shown in Figure 5). These CD changes indicated that the secondary structure of the target protein was influenced by the small molecule **7**.

## 3. Materials and Methods 

### 3.1. General Information

Optical rotations were measured on an MCP300 polarimeter (Anton Paar, Graz, Austria) equipped with a thermally jacketed 10 cm cell at 20 °C. Sample concentrations (c) are given in g/100 mL. Melting points were obtained on a MP420 auto melting point system (Hanon, Jinan, China) and are uncorrected. IR spectra were obtained on a Tensor 27 spectrometer (Bruker, Ettlingen, Germany). NMR spectra were recorded in CDCl_3_ using an AVANCE III 400 MHz spectrometer (Bruker, Ettlingen, Germany). The solvent residual peak was used as an internal reference for chemical shifts (in ppm). Coupling constants, *J*, are reported in Hertz (Hz). ESI-MS spectra were measured on a LTQ Fleet instrument (Thermo Fisher Scientific, Waltham, MA, USA). HR ESI-MS data were obtained by an Triple TOF 4600 system (AB Sciex, Redwood City, CA, USA). ECD spectra were measured on a Chirascan CD Spectrometer (Applied Photophysics, London, Britain). Column chromatography was performed on silica gel (90–150 μm, Dingkang, Qingdao, China) and Chromatorex C_18_ gel (40−75 μm, Fuji Silysia Chemical LTD, Aichi-ken, Japan). GF_254_ plates were used for thin-layer chromatography (TLC). HPLC analysis and preparations were performed on a 1525 instrument (Waters, Milford, MA, USA) equipped with a 250 × 10 mm, 5 μm semi-preparative column (Dr. Maisch GmbH, Ammerbuch, Germany). The reagents 4-hydroxycoumarin (98%), paraformaldehyde (96%), caryophyllene (90% GC) 6-chloro-4-hydroxycoumarin (97%, TCI) and 6-chloro-4-hydroxycoumarin (98%, TCI) were purchased from Energy Chemical Company (Shanghai, China).

### 3.2. Synthetic Procedures and Product Characterization

#### 3.2.1. Products Starting from 4-Hydroxycoumarin

To a 50 mL reaction tube was added 4-hydroxycoumarin (170 mg, 1.049 mmol) and paraformaldehyde (252 mg). The reaction tube was then flushed with argon and evacuated three times. Anhydrous 1,4-dioxane (5 mL) was added by syringe, and the mixture was stirred under reflux for 1 h. Then anhydrous 1,4-dioxane (2 mL) and *β*-caryophyllene (630 μL, 3.150 mmol) were injected into the reaction tube. The resulting solution was stirred for 24 h at reflux under an argon atmosphere, after which complete conversion of the starting material was confirmed by TLC inspection. After cooling to rt, the reaction mixture was washed with 10 mL of saturated Na_2_CO_3_. The organic layer was collected and removed the solvent under vacuum to yield crude product (869 mg). The crude products were separated on silica gel column (EtOAc in petroleum ether gradient, from 25:1 to 5:1, v/v) to 5 fractions (Fr. A~E). These fractions were further purified by HPLC using the Dr. Maisch GmbH semi-preparative column, eluting with 100% MeCN, to yield compounds **1a** (110 mg, 28%), **2a** (48 mg, 12%), **3a** (31 mg, 8%), **4a** (10 mg, 3%) and **5a** (7 mg, 2%).

**Compound 1a:** oil; ${\left[\alpha \right]}_{D}^{20}$ = −68.00 (*c* 0.05 in MeCN); UV (MeCN) λ_max_ nm (log ε) 205(4.57), 269 (3.84), 280 (3.91), 304 (3.88), 316 (3.70); IR (KBr, cm^−1^): 2932, 1712, 1636, 1396, 1219, 1042, 772; ^1^H-NMR (CDCl_3_) *δ* 0.97 (s, 3 H, H_3_-14), 1.01 (s, 3 H, H_3_-15), 1.24 (s, 3 H, H_3_-12), 1.42–1.56 (m, 1 H, H-6a), 1.60–1.64 (m, 1 H, H-2b), 1.67 (d, *J* = 9.4 Hz, 1 H, H-2a), 1.70–1.74 (m, 1 H, H-1), 1.75–1.82 (m, 1 H, H-6b), 1.83–1.88 (m, 1 H, H-10b), 1.88–1.95 (m, 1 H, 10a), 2.03–2.09 (m, 1 H, H-5), 2.12–2.14 (m, 1 H, H-3a), 2.13–2.17 (m, 1 H, H-16a), 2.17–2.22 (m, 1 H, H-7a), 2.22–2.29 (m, 1 H, H-3b), 2.42–2.46 (m, 1 H, H-7b), 2.47–2.52 (m, 1 H, H-9), 2.59–2.71 (m, 1 H, H-16b), 4.92 (s, 1 H, H-13b), 4.88 (s, 1 H, H-13a), 7.27 (t, *J* = 7.4 Hz, 1 H, H-22), 7.31 (d, *J* = 8.2 Hz, 1 H, H-24), 7.49 (td, *J* = 8.2, 1.2 Hz, 1 H, H-23), 7.76 (dd, *J* = 7.8, 1.2 Hz, 1 H, H-21); ^13^C-NMR (CDCl_3_) *δ* 21.1(C-14), 22.2(C-12), 22.3(C-2), 26.1(C-16), 30.2(C-15), 33.4(C-6), 33.8(C-11), 34.0(C-5), 35.2 (C-7), 36.5(C-10), 37.8(C-3), 41.5(C-9), 53.5(C-1), 84.4(C-4), 99.8(C-18), 110.6(C-13), 116.1(C-23), 116.5(C-20), 122.3(C-21), 123.6(C-22), 131.1(C-24), 151.8(C-25), 152.5(C-8), 158.8(C-19), 162.9(C-17); HRESIMS *m*/*z* [M + H]^+^ calcd for C_25_H_31_O_3_ 379.2273, found 379.2245.

**Compound 2a:** oil; ${\left[\alpha \right]}_{D}^{20}$ = +24.80 (*c* 0.05 in MeCN); UV (MeCN) λ_max_ nm (log ε) 206(4.60), 269 (3.98), 280 (4.05), 304 (4.02), 316 (3.84); IR (KBr, cm^−1^): 2939, 2864, 1711, 1634, 1395, 1042, 754; ^1^H-NMR (CDCl_3_) *δ*, 0.82–0.91 (m, 1 H, H-6a), 0.95 (s, 3 H, H_3_-14), 1.00 (s, 3 H, H_3_-15), 1.16 (s, 3 H, H_3_-12), 1.40–1.49 (m, 1 H, H-6b), 1.54–1.60 (m, 2 H, H-2a + H-2b), 1.63 (dd, *J* = 9.2, 2.2 Hz, 1 H, H-1), 1.70 (d, *J* = 9.8 Hz, 1 H, H-10b), 1.72–1.80 (m, 1 H, H-10a), 1.88 (dd, *J* = 15.5, 8.0 Hz, 1 H, H-7a), 2.03–2.09 (m, 1 H, H-5), 2.09–2.12 (m, 1 H, H-3a), 2.13–2.18 (m, 1 H, H-16a), 2.30 (dd, *J* = 15.7, 10.2 Hz, 1 H, H-3b), 2.43–2.52 (m, 1 H, H-7b), 2.62 (q, *J* = 9.1 Hz, 1 H, H-9), 2.85 (dd, *J* = 14.9, 2.7 Hz, 1 H, H-16b), 4.75 (s, 1 H, H-13b), 4.84 (s, 1 H, H-13a), 7.26 (t, *J* = 7.4 Hz, 1 H, H-22), 7.31 (d, *J* = 8.2 Hz, 1 H, H-24), 7.50 (td, *J* = 7.8, 1.0 Hz, 1 H, H-23), 7.77 (dd, *J* = 8.0, 1.4 Hz, 1 H, H-21); ^13^C-NMR (CDCl_3_) *δ* 20.0 (C-14), 22.5 (C-12), 22.8 (C-2), 25.3 (C-16), 29.7 (C-15), 33.4 (C-6), 33.7 (C-11), 34.8 (C-5), 36.5 (C-7), 38.5 (C-10), 38.9 (C-3), 42.5 (C-9), 56.3 (C-1), 84.4 (C-4), 99.8 (C-18), 110.1 (C-13), 116.1 (C-23), 116.5 (C-20), 122.4 (C-21), 123.6 (C-22), 131.2 (C-23), 152.5 (C-25), 154.5 (C-8), 159.1 (C-19), 162.9 (C-17); HRESIMS *m*/*z* [M + Na]^+^ calcd for C_25_H_30_O_3_Na 401.2093, found 401.2068.

**Compound 3a:** oil; ${\left[\alpha \right]}_{D}^{20}$ = +15.60 (*c* 0.05 in MeCN); UV (MeCN) λ_max_ nm (log ε) 204(4.58), 223 (4.43), 274 (4.16), 286 (4.09); IR (KBr, cm^−1^): 2934, 2864, 1568, 1420, 760; ^1^H-NMR (CDCl_3_) *δ* 1.00 (s, 6 H, H_3_-14, 15), 1.29 (s, 3 H, H_3_-12), 1.41–1.52 (m, 1 H, H-6a), 1.59–1.67 (m, 2 H, H-2a + H-2b), 1.68–1.76 (m, 2 H, H-1 + H-6b), 1.81–1.88 (m, 1 H, H-10b), 1.88–1.94 (m, 1 H, H-10a), 1.99 (ddd, *J* = 15.5, 6.3, 2.5 Hz, 1 H, H-5), 2.11–2.13 (m, 1 H, H-3a), 2.14–2.15 (m, 1 H, H-16a), 2.15–2.24 (m, 2 H, H-3b + H-7a), 2.40–2.50 (m, 2 H, H-7b + H-9), 2.74–2.84 (m, 1 H, H-16b), 4.87 (s, 1 H, H-13a), 4.91 (s, 1 H, H-13b), 7.35 (br. s, 1 H, H-24), 7.36 (br. s, 1 H, H-22), 7.57 (td, *J* = 7.7, 1.8 Hz, 1 H, H-23), 8.15–8.21 (m, 1 H, H-21); ^13^C-NMR (CDCl_3_) *δ* 21.0 (C-14), 22.1 (C-12), 22.3 (C-2), 24.8 (C-16), 30.2 (C-15), 32.9 (C-6), 33.9 (C-5), 34.3 (C-11), 35.1 (C-7), 36.5 (C-10), 37.8 (C-3), 41.4 (C-9), 53.5 (C-1), 88.5 (C-4), 95.5 (C-18), 109.7 (C-13), 117.0 (C-24), 122.7 (C-22), 124.7 (C-20), 125.7 (C-21), 132.5 (C-23), 151.7 (C-25), 153.2 (C-8), 162.8 (C-17), 177.3 (C-19); HRESIMS *m*/*z* [M + H]^+^ calcd for C_25_H_31_O_3_ 379.2273, found 379.2265.

**Compound 4a:** oil; ${\left[\alpha \right]}_{D}^{20}$ = −79.39 (*c* 0.05 in MeCN); UV (MeCN) λ_max_ nm (log ε) 206(4.61), 223 (4.53), 274 (4.29), 286 (4.21); IR (KBr, cm^−1^): 2936, 2860, 1624, 1568, 1420, 1261, 760; ^1^H-NMR (CDCl_3_) *δ* 0.99 (s, 3 H, H_3_-14), 1.01 (s, 3 H, H_3_-15), 1.23 (s, 3 H, H_3_-12), 1.53–1.57 (m, 2 H, H-6a + H-6b), 1.59–1.65 (m, 2 H, H-2a + H-2b), 1.71 (m, 1 H, H-1), 1.77 (m, 1 H, H-10a), 1.78–1.82 (m, 1 H, H-10b), 1.86 (td, *J* = 7.8, 1.2 Hz, 1 H, H-5), 2.04–2.10 (m, 1 H, H-3a), 2.11–2.18 (m, 2 H, H-7a + H-16a), 2.21–2.30 (m, 1 H, H-3b), 2.43–2.52 (m, 1 H, H-7b), 2.59–2.70 (q, *J* = 7.8 Hz, 1 H, H-9), 3.02 (dd, *J* = 15.1, 4.1 Hz, 1 H, 16b), 4.76 (br. t, *J* = 1.6 Hz, 1 H, H-13a), 4.85 (br. d, *J* = 1.6 Hz, 1 H, H-13b), 7.35–7.41 (m, 2 H, H-24 + H-22), 7.60 (ddd, *J* = 8.4, 7.0, 1.8 Hz, 1 H, H-23), 8.21 (dt, *J* = 8.2, 0.8 Hz, 1 H, H-21); ^13^C-NMR (CDCl_3_) *δ* 20.0 (C-14), 22.6 (C-12), 22.9 (C-2), 24.0 (C-16), 29.7 (C-15), 33.2 (C-6), 33.4 (C-11), 35.2 (C-5), 36.6 (C-7), 38.5 (C-10), 38.7 (C-3), 42.5 (C-9), 56.4 (C-1), 88.6 (C-4), 95.5 (C-18), 110.2 (C-13), 117.1 (C-24), 122.7 (C-22), 124.8 (C-20), 125.7 (C-21), 132.6 (C-23), 153.3 (C-25), 154.5 (C-8), 163.0 (C-17), 177.4 (C-19); HRESIMS *m*/*z* [M + H]^+^ calcd. for C_25_H_31_O_3_ 379.2273, found 379.2248.

**Compound 5a:** oil; ${\left[\alpha \right]}_{D}^{20}$ = −10.20 (*c* 0.05 in MeCN); UV (MeCN) λ_max_ nm (log ε) 202(4.43), 254 (3.89), 311 (3.38); IR (KBr, cm^−1^):3470, 2934, 1630, 1524, 776; ^1^H-NMR (CDCl_3_) *δ* 0.92 (s, 3 H, H_3_-14), 0.93 (s, 3 H, H_3_-15), 1.24–1.29 (m, 2 H, H-6a + H-6b), 1.46–1.55 (m, 2 H, H-2a + H-2b), 1.57–1.60 (m, 1 H, H-1), 1.70–1.74 (m, 1 H, H-10b), 1.78–1.85 (m, 3 H, h-3a, 5, 10a), 2.25 (m, 1 H, H-16a), 2.27–2.38 (m, 2 H, H-3b + H-7a), 2.41–2.46 (m, 2 H, H-7b + H-9), 2.90 (d, *J* = 9.8 Hz, 1 H, H-16b), 4.91 (s, 1 H, H-12a), 4.93 (s, 1 H, H-12b), 5.15 (s, 1 H, H-13a), 5.19 (s, 1 H, H-13b), 7.01 (s, 1 H, HO-19), 7.25–7.28 (overlapped, 1 H, H-21), 7.29–7.34 (overlapped, 1 H, H-24), 7.52 (ddd, *J* = 8.4, 7.2, 1.6 Hz, 1 H, H-22), 7.80 (dd, *J* = 7.8, 1.6 Hz, 1 H, H-23); ^13^C-NMR (CDCl_3_) *δ* ppm 21.7(C-14), 29.7(C-15), 32.7(C-2), 33.0(C-16), 33.2(C-11), 35.9(C-6), 36.0(C-7), 36.5(C-10), 40.5(C-3), 41.9(C-9), 47.6(C-5), 54.5(C-1), 104.1(C-18), 109.6(C-12), 110.2(C-13), 115.4(C-20), 116.4(C-24), 123.1(C-21), 123.8(C-22), 131.7(C-23), 152.0(C-25), 152.4(C-8), 160.0(C-4), 161.0(C-19), 163.4(C-17); HRESIMS *m*/*z* [M + H]^+^ calcd. for C_25_H_31_O_3_ 379.2273, found 379.2256.

#### 3.2.2. Products Starting from 6-Chloro-4-Hydroxycoumarin

The procedure was the same as the above reaction. Paraformaldehyde (248 mg), 6-chloro-4-hydroxycoumarin (193 mg, 0.982 mmol) and β-caryophyllene (690 μL, 3.450 mmol) were used as starting materials and 919 mg of crude product were obtained from the reaction. After the same separation and purification procedure as above, five products were isolated: **1b** (41%, 168 mg), **2b** (18%, 71 mg), **3b** (14%, 59 mg), **4b** (6%, 25 mg), **5b** (2%, 7 mg).

**Compound 1b:** oil; ${\left[\alpha \right]}_{D}^{20}$ = −176.19 (*c* 0.05 in MeCN); UV (MeCN) λ_max_ nm (log ε) 204(4.62), 273 (4.12), 285 (4.15), 311 (4.12), 325 (4.00); IR (KBr, cm^−1^): 2936, 1717, 1636, 1385, 758; ^1^H-NMR (CDCl_3_) *δ* 0.99 (s, 3 H, H_3_-14), 1.02 (s, 3 H, H_3_-15), 1.24 (s, 3 H, H_3_-12), 1.46–1.56 (m, 1 H, H-6a), 1.59–1.65 (m, 2 H, H-2a + H-2b), 1.70 (d, *J* = 10.2 Hz, 1 H, H-1), 1.76–1.87 (m, 2 H, H-6b + H-10b), 1.88–1.94 (m, 1 H, H-10a), 2.02–2.08 (m, 1 H, H-5), 2.09–2.14 (m, 2 H, H-3a + H-16a), 2.15–2.20 (m, 1 H, H-7a), 2.22–2.29 (m, 1 H, H-3b), 2.42–2.51 (m, 2 H, H-7b + H-9), 2.58–2.70 (m, 1 H, H-16b), 4.89 (s, 1 H, H-13a), 4.92 (s, 1 H, H-13b), 7.25 (d, *J* = 8.6 Hz, 1 H, H-24), 7.44 (dd, *J* = 8.8, 2.5 Hz, 1 H, H-23), 7.71 (d, *J* = 2.3 Hz, 1 H, H-21); ^13^C-NMR (CDCl_3_) *δ* ppm 21.1(C-14), 22.2(C-12), 22.3(C-2), 26.1(C-16), 30.2(C-15), 33.4(C-6), 33.9(C-11), 33.9(C-5) 35.2(C-7), 36.5(C-10), 37.8(C-3), 41.4(C-9), 53.6(C-1), 84.9(C-4), 100.7(C-18), 110.7(C-13), 117.2(C-20), 118.0(C-24), 121.9(C-21), 129.1(C-22), 131.1(C-23), 150.8(C-25), 151.8(C-8), 157.7(C-19), 162.4(C-17); HRESIMS *m*/*z* [M + H]^+^ calcd for C_25_H_30_ClO_3_ 413.1883, found 413.1864.

**Compound 2b:** white solid, mp 161.4–163.2 °C (CHCl_3_); ${\left[\alpha \right]}_{D}^{20}$ = +72.39 (*c* 0.05 in MeCN); UV (MeCN) λ_max_ nm (log ε) 209(4.61), 273 (3.93), 285 (3.95), 311 (3.93), 325 (3.81); IR (KBr, cm^−1^): 2936, 1715, 1634, 1385, 1042, 814; ^1^H-NMR (CDCl_3_) *δ* 0.97 (s, 3 H, H_3_-14), 1.02 (s, 3 H, H_3_-15), 1.17 (s, 3 H, H_3_-12), 1.50–1.58 (m, 2 H, H-6a + H-6b), 1.61 (d, *J* = 14.9 Hz, 2 H, H-2a + H-2b), 1.63–1.68 (m, 1 H, H-1), 1.69–1.73 (m, 1 H, H-10a), 1.75–1.79 (m, 1 H, H-10b), 1.89 (dd, *J* = 15.7, 8.6 Hz, 1 H, H-5), 2.08–2.18 (m, 3 H, H-3a + H-7a + H-16a), 2.31 (dd, *J* = 15.5, 10.4 Hz, 1 H, H-3b), 2.43–2.53 (m, 1 H, H-7b), 2.63 (q, *J* = 9.1 Hz, 1 H, H-9), 2.85 (dd, *J* = 15.5, 3.3 Hz, 1 H, H-16b), 4.77 (s, 1 H, H-13a), 4.85 (s, 1 H, H-13b), 7.26 (d, *J* = 9.0 Hz, 1 H, H-24), 7.45 (dd, *J* = 8.8, 2.5 Hz, 1 H, H-23), 7.72 (d, *J* = 2.7 Hz, 1 H, H-21); ^13^C-NMR (CDCl_3_) *δ* 20.0 (C-14), 22.6 (C-12), 22.8 (C-2), 25.3 (C-16), 29.7 (C-15), 33.4 (C-11), 33.7 (C-6), 34.8 (C-5), 36.5 (C-7), 38.5 (C-10), 38.8 (C-3), 42.5 (C-9), 56.3 (C-1), 85.0 (C-4), 100.7 (C-18), 110.2 (C-13), 117.2 (C-20), 118.0 (C-24), 122.0 (C-21), 129.1 (C-22), 131.2 (C-23), 150.8 (C-25), 154.4 (C-8), 158.1 (C-19), 162.3 (C-17); HRESIMS *m*/*z* [M + H]^+^ calcd for C_25_H_30_ClO_3_ 413.1883, found 413.1881.

**Compound 3b:** oil; ${\left[\alpha \right]}_{D}^{20}$ = +22.60 (*c* 0.05 in MeCN); UV (MeCN) λ_max_ nm (log ε) 211(4.45), 282 (3.88); IR (KBr, cm^−1^): 2928, 1624, 1564, 1447, 772; ^1^H-NMR (CDCl_3_) *δ* 1.00 (s, 6 H, H_3_-14, 15), 1.29 (s, 3 H, H_3_-12), 1.43–1.52 (m, 1 H, H-6a), 1.61–1.73 (m, 4 H, H-1 + H-2a + H-2b + H-6b), 1.78–1.87 (m, 1 H, H-10a), 1.91 (td, *J* = 10.2, 5.5 Hz, 1 H, H-10b), 2.00 (ddd, *J* = 15.6, 6.4, 2.3 Hz, 1 H, H-5), 2.10–2.21 (m, 4 H, H-3a + H-3b + H-7a + H-16a), 2.40–2.51 (m, 2 H, H-7b + H-9), 2.73–2.84 (m, 1 H, H-16b), 4.88 (s, 1 H, H-13a), 4.92 (s, 1 H, H-13b), 7.25–7.34 (m, 1 H, H-24), 7.52 (dd, *J* = 8.8, 2.5 Hz, 1 H, H-23), 8.14 (d, *J* = 2.3 Hz, 1 H, H-21); ^13^C-NMR (CDCl_3_) *δ* 21.0 (C-14), 22.1 (C-12), 22.3 (C-2), 24.7 (C-16), 30.2 (C-15), 32.9 (C-6), 33.9 (C-11), 34.2 (C-5), 35.1 (C-7), 36.4 (C-10), 37.8 (C-3), 41.4 (C-9), 53.6 (C-1), 89.0 (C-4), 95.7 (C-18), 110.8 (C-13), 118.7 (C-24), 123.7 (C-20), 125.2 (C-21), 130.5 (C-22), 132.6 (C-23), 151.5 (C-25), 151.6 (C-8), 162.9 (C-17), 176.1(C-19); HRESIMS *m*/*z* [M + H]^+^ calcd for C_25_H_30_ClO_3_ 413.1883, found 413.1862.

**Compound 4b:** white solid, mp 137.0–139.4 °C (CHCl_3_); ${\left[\alpha \right]}_{D}^{20}$ = −44.40 (*c* 0.05 in MeCN); UV (MeCN) λ_max_ nm (log ε) 211(4.47), 282 (3.90); IR (KBr, cm^−1^): 2957, 1624, 1562, 1447, 1259, 808; ^1^H-NMR (CDCl_3_) *δ* 0.99 (s, 3 H, H_3_-14), 1.01 (s, 3 H, H_3_-15), 1.23 (s, 3 H, H_3_-12), 1.53–1.57 (m, 2 H, H-6a + H-6b), 1.58–1.68 (m, 3 H, H-1 + H-2a + H-2b), 1.71 (d, *J* = 10.2 Hz, 1 H, H-10a), 1.75–1.79 (m, 1 H, H-10b), 1.82–1.89 (m, 1 H, H-5), 2.09–2.19 (m, 3 H, H-3a + H-7a + H-16a), 2.25 (dd, *J* = 15.8, 10.0 Hz, 1 H, H-3b), 2.42–2.51 (m, 1 H, H-7b), 2.64 (q, *J* = 9.0 Hz, 1 H, H-9), 2.99 (dd, *J* = 15.1, 4.1 Hz, 1 H, H-16b), 4.76 (br. s, 1 H, H-13a), 4.85 (br. s, 1 H, H-13b), 7.33 (d, *J* = 9.0 Hz, 1 H, H-24), 7.53 (dd, *J* = 8.8, 2.5 Hz, 1 H, H-23), 8.16 (d, *J* = 2.7 Hz, 1 H, H-21); ^13^C-NMR (CDCl_3_) *δ* 20.0 (C-14), 22.6 (C-12), 22.9 (C-2), 23.9 (C-16), 29.7 (C-15), 33.2 (C-6), 33.4 (C-11), 35.1 (C-5), 36.5 (C-7), 38.5 (C-10), 38.7 (C-3), 42.5 (C-9), 56.2 (C-1), 89.0 (C-4), 95.7 (C-18), 110.3 (C-13), 118.7 (C-24), 123.7 (C-20), 125.2 (C-21), 130.6 (C-22), 132.6 (C-23), 151.5 (C-25), 154.4 (C-8), 163.1 (C-17), 176.1 (C-19); HRESIMS *m*/*z* [M + H]^+^ calcd for C_25_H_30_ClO_3_ 413.1883, found 413.1874.

**Compound 5b:** oil; ${\left[\alpha \right]}_{D}^{20}$ = −6.80 (*c* 0.05 in MeCN); UV (MeCN) λ_max_ nm (log ε) 201(4.05), 219 (4.02), 319 (2.97); IR (KBr, cm^−1^): 3285, 2934, 2864, 1711, 1638, 1466, 1277, 758; ^1^H-NMR (CDCl_3_) *δ* 0.93 (s, 6 H, H_3_-14, 15), 1.25–1.33 (m, 2 H, H-6a + H-6b), 1.41–1.50 (m, 2 H, H-2a + H-2b), 1.56–1.59 (m, 1 H, H-1), 1.71–1.73 (m, 1 H, H-10a), 1.81 (m, 3 H, H-3a + H-5 + H-10b), 2.22–2.28 (m, 1 H, H-16a), 2.31–2.40 (m, 2 H, H-3b, 7a), 2.41–2.45 (m, 2 H, H-7b + H-9), 2.89 (m, *J* = 10.2 Hz, 1 H, H-16b), 4.90 (s, 1 H, H-13a), 4.93 (s, 1 H, H-13b), 5.16 (s, 1 H, H-12a), 5.19 (s, 1 H, H-12b), 7.10 (s, 1 H, HO-10), 7.25 (d, *J* = 9.0 Hz, 1 H, H-24), 7.46 (dd, *J* = 8.6, 2.3 Hz, 1 H, H-23), 7.77 (d, *J* = 2.3 Hz, 1 H, H-21); ^3^C-NMR (CDCl_3_) *δ* 21.6 (C-14), 29.7 (C-15), 32.7 (C-2), 32.9 (C-16), 33.2 (C-11), 35.9 (C-6), 36.0 (C-7), 36.4 (C-10), 40.6 (C-3), 41.9 (C-9), 47.6 (C-5), 54.5 (C-1), 105.0 (C-18), 109.6 (C-12), 110.3 (C-13), 116.6 (C-20), 117.8 (C-24), 122.8 (C-21), 130.9 (C-22), 131.7 (C-23), 150.7 (C-25), 152.0 (C-8), 159.0 (C-4), 161.0 (C-19), 162.9 (C-17); HRESIMS *m*/*z* [M + H]^+^ calcd for C_25_H_30_ClO_3_ 413.1883, found 413.1868.

#### 3.2.3. Products Starting from 6-Bromo-4-Hydroxycoumarin

The procedure was the same as the above reaction. Paraformaldehyde (203 mg), 6-bromo-4-hydroxycoumarin (188 mg, 0.780 mmol) and β-caryophyllene (565 μL, 2.825 mmol) were used as starting materials and 772 mg of crude product was obtained from the reaction. After the same separation and purification procedure as above, five products were separated: **1c** (41%, 142 mg), **2c** (18%, 63 mg), **3c** (14%, 48 mg), **4c** (5%, 18 mg), **5c** (2%, 8 mg).

**Compound 1c:** oil; ${\left[\alpha \right]}_{D}^{20}$ = −90.99 (*c* 0.05 in MeCN); UV (MeCN) λ_max_ nm (log ε) 210(4.55), 274 (3.82), 286 (3.84), 312 (3.81), 325 (3.71); IR (KBr, cm^−1^): 2936, 1715, 1634, 1385, 764; ^1^H-NMR (CDCl_3_) *δ* 0.99 (s, 3 H, H_3_-14), 1.02 (s, 3 H, H_3_-15), 1.23 (s, 3 H, H_3_-12), 1.51 (ddd, *J* = 10.2, 7.8, 3.1 Hz, 1 H, H-6a), 1.62–1.69 (m, 2 H, H-2a + H-2b), 1.70–1.74 (m, 1 H, H-1), 1.75–1.80 (m, 1 H, H-6b), 1.81–1.86 (m, 1 H, H-10a), 1.87–1.93 (m, 1 H, H-10b), 2.02–2.08 (m, 1 H, H-5), 2019–2.12 (m, 1 H, H-3a), 2.12–2.15 (m, 1 H, H-16a), 2.18–2.21 (m, 1 H, H-7a), 2.21–2.29 (m, 1 H, H-3b), 2.42–2.51 (m, 2 H, H-7b + H-9), 2.59–2.70 (m, 1 H, H-16b), 4.88 (s, 1 H, H-13a), 4.92 (s, 1 H, H-13b), 7.18 (d, *J* = 9.0 Hz, 1 H, H-24), 7.57 (dd, *J* = 8.6, 2.3 Hz, 1 H, H-23), 7.85 (d, *J* = 2.3 Hz, 1 H, H-21); ^13^C-NMR (CDCl_3_) *δ* 21.1 (C-14), 22.2 (C-12), 22.3 (C-2), 26.1 (C-16), 30.2 (C-15), 33.4 (C-6), 33.8 (C-5), 33.9 (C-11), 35.2 (C-7), 36.5 (C-10), 37.8 (C-3), 41.4 (C-9), 53.6 (C-1), 85.0 (C-4), 100.7 (C-18), 110.7(C-13), 116.5(C-22), 117.7(C-24), 118.3(C-20), 124.9(C-21), 133.9(C-23), 151.3(C-25), 151.8(C-8), 157.6(C-19), 162.3(C-17); HRESIMS *m*/*z* [M + Na]^+^ calcd for C_25_H_29_BrO_3_Na 479.1198, found 479.1184.

**Compound 2c:** white solid, mp 149.0–151.4 °C (CHCl_3_); ${\left[\alpha \right]}_{D}^{20}$ = +67.40 (*c* 0.05 in MeCN); UV (MeCN) λ_max_ nm (log ε) 210(4.58), 274 (3.85), 285 (3.87), 312 (3.84), 325 (3.74); IR (KBr, cm^−1^): 2934, 1715, 1634, 1363, 812; ^1^H-NMR (CDCl_3_) *δ* 0.97 (s, 3 H, H_3_-14), 1.02 (s, 3 H, H_3_-15), 1.16 (s, 3 H, H_3_-12), 1.40–1.47 (m, 1 H, H-6a), 1.55–1.60 (m, 2 H, H-2a + H-2b), 1.61–1.63 (m, 1 H, H-1), 1.64–1.68 (m, 1 H, H-6b), 1.68–1.73 (m, 1 H, H-10a), 1.74–1.81 (m, 1 H, H-10b), 1.89 (dd, *J* = 15.7, 8.2 Hz, 1 H, H-5), 2.03–2.09 (m, 1 H, H-3a), 2.11–2.17 (m, 2 H, H-7a + H-16a), 2.31 (dd, *J* = 15.7, 10.2 Hz, 1 H, H-3b), 2.43–2.53 (m, 1 H, H-7b), 2.62 (q, *J* = 9.0 Hz, 1 H, H-9), 2.85 (dd, *J* = 15.7, 3.1 Hz, 1 H, H-16b), 4.76 (s, 1 H, H-13a), 4.85 (s, 1 H, H-13b), 7.20 (d, *J* = 8.6 Hz, 1 H, H-24), 7.59 (dd, *J* = 9.0, 2.3 Hz, 1 H, H-23), 7.86 (d, *J* = 2.3 Hz, 1 H, H-21); ^13^C-NMR (CDCl_3_) *δ* 20.0 (C-14), 22.6 (C-12), 22.9 (C-2), 25.3 (C-16), 29.7 (C-15), 33.4 (C-11), 33.7 (C-6), 34.8 (C-5), 36.5 (C-7), 38.5 (C-10), 38.8 (C-3), 42.5 (C-9), 56.3 (C-1), 85.0 (C-4), 100.7 (C-18), 110.2 (C-13), 116.5 (C-22), 117.6 (C-24), 118.3 (C-20), 125.1 (C-21), 134.0 (C-23), 151.3 (C-25), 154.4 (C-8), 158.0 (C-19), 162.3 (C-17); HRESIMS *m*/*z* [M + H]^+^ calcd for C_25_H_30_BrO_3_ 457.1378, found 457.1360.

**Compound 3c:** oil; ${\left[\alpha \right]}_{D}^{20}$ = +34.20 (*c* 0.05 in MeCN); UV (MeCN) λ_max_ nm (log ε) 212(4.56), 282 (4.00); IR (KBr, cm^−1^): 2936, 1624, 1562, 1445, 1396, 756; ^1^H-NMR (CDCl_3_) *δ* 1.01 (s, 6 H, H_3_-14 + H_3_-15), 1.29 (s, 3 H, H_3_-12), 1.42–1.53 (m, 1 H, H-6a), 1.62–1.72 (m, 4 H, H-1 + H-2a + H-2b + H-6b), 1.77–1.87 (m, 1 H, H-10a), 1.92 (td, *J* = 10.2, 5.5 Hz, 1 H, H-10b), 2.00 (ddd, *J* = 15.3, 6.3, 2.3 Hz, 1 H, H-5), 2.11–2.20 (m, 4 H, H-3a + H-3b + H-7a + H-16a), 2.41–2.50 (m, 2 H, H-7b + H-9), 2.73–2.84 (m, 1 H, H-16b), 4.88 (s, 1 H, H-13a), 4.92 (s, 1 H, H-13b), 7.25 (d, *J* = 8.6 Hz, 1 H, H-24), 7.66 (dd, *J* = 9.0, 2.3 Hz, 1 H, H-23), 8.30 (d, *J* = 2.3 Hz, 1 H, H-21); ^13^C-NMR (CDCl_3_) *δ* 21.0 (C-14), 22.1 (C-12), 22.3 (C-2), 24.7 (C-16), 30.2 (C-15), 32.9 (C-6), 33.9 (C-11), 34.2 (C-5), 35.1 (C-7), 36.4 (C-10), 37.8 (C-3), 41.4 (C-9), 53.6 (C-1), 89.1 (C-4), 95.7 (C-18), 110.8 (C-13), 118.0 (C-24), 119.0 (C-22), 124.1 (C-20), 128.3 (C-21), 135.4 (C-23), 151.6 (C-25), 151.9 (C-8), 162.9 (C-17), 175.9 (C-19); HRESIMS *m*/*z* [M + H]^+^ calcd for C_25_H_30_BrO_3_ 457.1378, found 457.1357.

**Compound 4c:** oil; ${\left[\alpha \right]}_{D}^{20}$ = −29.00 (*c* 0.05 in MeCN); UV (MeCN) λ_max_ nm (log ε) 212(4.26), 282 (3.70); IR (KBr, cm^−1^): 2941, 1622, 1562, 1447, 768; ^1^H-NMR (CDCl_3_) *δ* 0.98 (s, 3 H, H_3_-14), 0.99 (s, 3 H, H_3_-15), 1.22 (s, 3 H, H_3_-12), 1.50–1.57 (m, 3 H, H-2a + H-2b + H-6a), 1.58–1.60 (m, 1 H, H-6b), 1.60–1.63 (m, 1 H, H-1), 1.67–1.78 (m, 2 H, H-10a, 10b), 1.82–1.88 (m, 1 H, H-5), 2.09–2.18 (m, 3 H, H-3a + H-7a + H-16a), 2.24 (dd, *J* = 15.7, 9.4 Hz, 1 H, H-3b), 2.41–2.50 (m, 1 H, H-7b), 2.63 (q, *J* = 9.4 Hz, 1 H, H-9), 2.98 (dd, *J* = 15.3, 4.3 Hz, 1 H, H-16b), 4.75 (br. t, *J* = 1.6, 1.6 Hz, 1 H, H-13a), 4.84 (br. d, *J* = 0.8 Hz, 1 H, H-13b), 7.25 (d, *J* = 5.1 Hz, 1 H, H-24), 7.66 (dd, *J* = 8.6, 2.3 Hz, 1 H, H-23), 8.31 (d, *J* = 2.3 Hz, 1 H, h-21); ^13^C-NMR (CDCl_3_) *δ* 19.7 (C-14), 22.3 (C-12), 22.6 (C-2), 23.6 (C-16), 29.4 (C-15), 32.9 (C-6), 33.1 (C-11), 34.8 (C-5), 36.2 (C-7), 38.2 (C-10), 38.4 (C-3), 42.2 (C-9), 55.9 (C-1), 88.8 (C-4), 95.5 (C-18), 110.0 (C-13), 117.7 (C-24), 118.7 (C-22), 123.8 (C-20), 128.1 (C-21), 135.1 (C-23), 151.7 (C-25), 154.0 (C-8), 162.7 (C-17), 175.7 (C-19); HRESIMS *m*/*z* [M + H]^+^ calcd for C_25_H_30_BrO_3_ 457.1378, found 457.1367.

**Compound 5c:** oil; ${\left[\alpha \right]}_{D}^{20}$ = −5.40 (*c* 0.05 in MeCN); UV (MeCN) λ_max_ nm (log ε) 203(4.14), 222 (4.12), 318 (3.07); IR (KBr, cm^−1^): 3659, 2934, 1713, 1464, 1275, 770; ^1^H-NMR (CDCl_3_) *δ* 0.93 (s, 6 H, H_3_-14, 15), 1.25–1.35 (m, 2 H, H-6a, 6b), 1.49–1.60 (m, 3 H, H-1 + H-2a + H-2b), 1.70–1.73 (m, 1 H, H-10a), 1.49–1.84 (m, 3 H, H-3a + H-5 + H-10b), 2.21–2.29 (m, 1 H, H-16a), 2.36 (d, *J* = 3.5 Hz, 1 H, H-7a), 2.41–2.48 (m, 3 H, H-3b + H-7b + H-9), 2.88 (d, *J* = 10.2 Hz, 1 H, H-16b), 4.90 (s, 1 H, H-13a), 4.92 (s, 1 H, H-13b), 5.16 (s, 1 H, H-12a), 5.19 (s, 1 H, H-12b), 7.12 (br. s., 1 H, HO-19), 7.20 (d, *J* = 9.0 Hz, 1 H, H-24), 7.60 (dd, *J* = 8.8, 2.2 Hz, 1 H, H-23), 7.93 (d, *J* = 2.3 Hz, 1 H, H-21); ^13^C-NMR (CDCl_3_) *δ* 21.6 (C-14), 29.7 (C-15), 32.7 (C-2), 32.9 (C-16), 33.1 (C-11), 35.8 (C-6), 36.0 (C-7), 36.4 (C-10), 40.5 (C-3), 41.8 (C-9), 47.5 (C-5), 54.5 (C-1), 105.0 (C-18), 109.6 (C-12), 110.3 (C-13), 116.6 (C-20), 117.0 (C-22), 118.1 (C-24), 125.8 (C-21), 134.5 (C-23), 151.2 (C-25), 152.0 (C-8), 158.9 (C-4), 160.9 (C-19), 162.8 (C-17); HRESIMS *m*/*z* [M + H]^+^ calcd for C_25_H_30_BrO_3_ 457.1378, found 457.1356.

#### 3.2.4. Product **6** Starting from Syncarpic Acid

Syncarpic acid was synthesized from 2′,4′,6′-trihydroxyacetophenone according to our previously reported procedure [29]. Then, to a dry reaction tube, was added syncarpic acid (50 mg, 0.275 mmol), ZnI_2_ (53 mg, 0.166 mmol), anhydrous toluene (3 mL) and β-caryophyllene (168 mg, 0.824 mmol). After stirring for 18 h at 110 °C, the mixture was concentrated. To the residue was added 5 mL water and the mixture was then extracted with EtOAc (10 mL) three times. The organic layers were combined, dried over Na_2_SO_4_ and the solvent removed under reduced pressure to yield the crude product, which was then separated on a silica gel column (petroleum ether-EtOAc 50:1) to afford oily compound **6** (38 mg, 36%).

**Compound 6**: oil; ${\left[\alpha \right]}_{D}^{20}$ = +135.6 (c 0.05 in MeCN); IR (KBr, cm^−1^): 3096, 3058, 1704, 1648, 1510, 1202; ^1^H- NMR (CDCl_3_) δ 0.95 (s, 6 H, H_3_-12, 15), 0.97 (s, 3 H, H_3_-14), 1.13(m, 1 H, H-10a), 1.19–1.28 (m, 1 H, H-5a), 1.30 (s, 6 H, Me-17), 1.34 (s, 3 H, H_3_-19a), 1.35–1.40 (m, 1 H, H-13a), 1.41 (s, 3 H, Me-19b), 1.43–1.57 (m, 4 H, H-3a + H-5b + H-7a), 1.66–1.76 (m, 2 H, H-6a + H-6b), 1.79–1.88 (m, 1 H, H-7b), 2.05 (dd, 13.3, 1.6, 1 H, H-13b), 2.27–2.43 (m, 2 H, H-9 + H-10b), 5.37(s, 1 H, H-21); ^13^C-NMR (CDCl_3_) δ 20.6 (C-14), 20.8 (C-6), 24.3 (Me-17), 24.4 (C-2), 24.8 (Me-19), 25.6 (Me-17), 25.7 (Me-19), 30.6 (C-15), 34.1 (C-12), 34.7 (C-4), 34.9 (C-11), 35.6 (C-10), 36.1 (C-3), 39.9 (C-5), 40.5 (C-7), 44.4 (C-9), 47.5 (C-13), 47.7 (C-1), 48.8 (C-17), 55.2 (C-19), 85.3 (C-8), 104.7 (C-21), 174.6 (C-16), 199.1 (C-20), 214.1 (C-18); HR ESIMS m/z [M + Na]^+^ calcd for C_25_H_38_O_3_Na 409.2718, found 409.2705.

#### 3.2.5. Product **7** Starting from Lawsone

To a dry reaction tube, was added lawsone (50 mg, 0.287 mmol), ZnI_2_ (55 mg, 0.172 mmol), anhydrous toluene (3 mL) and β-caryophyllene (176 mg, 0.863 mmol). After stirring for 18 h at 110 °C, the mixture was concentrated. Using the same procedure as above, the residue afforded compound **7** (35 mg, 32%) as an oil.

**Compound 7**: oil; ${\left[\alpha \right]}_{D}^{20}$ = +92.4 (c 0.05 in MeCN); IR (KBr, cm^−1^): 3092, 3036, 1726, 1652, 1326, 1216, 996; ^1^H-NMR (CDCl_3_) *δ* 0.95 (s, 3 H, H_3_-14), 0.98 (s, 3 H, H_3_-12), 1.00 (s, 3 H, H_3_-15), 1.16 (dd, *J* = 7.8, 6.3 Hz, 1 H, H-10a), 1.30–1.34 (m, 1 H, H-5a), 1.35–1.41 (m, 1 H, H-2a), 1.45–1.49 (m, 1 H, H-10b), 1.52–1.60 (m, 4 H, H-2b + H-3a + H-5b + H-7a), 1.63 (s, 1 H, H-13a), 1.71–1.79 (m, 2 H, H-6a + H-6b), 1.85 (dd, *J* = 9.8, 7.4 Hz, 1 H, H-7b), 1.97 (ddd, *J* = 11.5, 9.6, 3.9 Hz, 1 H, H-1), 2.23–2.29 (m, 1 H, H-13b), 2.36–2.45 (m, 2 H, H-3b + H-9), 6.11 (s, 1 H, H-18), 7.65–7.74 (m, 2 H, H-22 + H-23), 8.03–8.06 (m, 1 H, H-24), 8.08–8.12 (m, 1 H, H-21); ^13^C-NMR (CDCl_3_) δ 20.5 (C-14), 20.5 (C-6), 24.1 (C-2), 30.0 (C-15), 33.6 (C-12), 34.6 (C-4), 34.6 (C-11), 35.1 (C-10), 35.6 (C-3), 39.5 (C-5), 40.3 (C-7), 44.3 (C-9), 47.0 (C-13), 47.2 (C-1), 86.1 (C-8), 114.7 (C-18), 125.9 (C-24), 126.6 (C-21), 131.4 (C-25), 131.9 (C-20), 133.0 (C-23), 133.9 (C-22), 156.8 (C-17), 181.0 (C-16), 185.0 (C-19); HR ESIMS m/z [M + Na]^+^ calcd for C_25_H_30_O_3_Na 401.2092, found 401.2091.

### 3.3. ECD Calculation Method

The calculation was performed as our previously reported procedure [45,46]. All the conformers of every calculated compound were searched by Conflex using the MMFF94s force field [47,48]. Further optimization was performed at B3LYP/6-31 + G(*d*,*p*) level in Gaussian 09 package [49]. The theoretical CD spectra were calculated by cam-B3LYP/TZVP and added in SpecDis [50] according to their Boltzmann-calculated distributions.

### 3.4. α-Glucosidase Inhibitory Assay

The α-glucosidase inhibitory assay of the synthesized compounds was performed as previously reported procedure [45]. α-Glucosidase (EC 3.2.1.20) isolated from *Saccharomyces cerevisiae* was purchased from Sigma (St. Louis, MO, USA). The enzyme was dissolved in 200 μL of 10 mM phosphate buffer (pH 6.80) and incubated with 12 μL of the test compound in DMSO at 37 °C for 5 min. Then the enzymatic reaction was started by the addition of 36 μL of 4-nitrophenyl *α*-D-glucopyranoside (*p*NPG) and kept under 37 °C for 40 min. The amount of released 4-nitrophenol was determined according to the absorbance at 400 nm. The primary screening was carried out at two concentrations (25 and 50 μM). While the IC_50_ assay was performed with five different concentrations around the IC_50_ values. In each set of experiments, the assay was performed in triplicate. The percentage inhibition of α-glucosidase activity was calculated via the following formula: Inhibition ratio (%) = 100 × (A_control_ − A_sample_)/A_control_. The IC_50_ values were calculated in Prism 7 using a nonlinear regression method with the normalized response and variable slope.

### 3.5. Kinetic Analysis of α-Glucosidase Inhibition

The kinetic parameters of α-glucosidase inhibition by compound were evaluated by the Lineweaver-Burk plots and its secondary plots. The double-reciprocal plots were constructed with enzyme reaction initial velocity (V) versus substrate (S) concentration (1/v vs. 1/[S]) in the absence (control) or presence of compound **9** at different levels (0–8 μM). The initial rate was measured by stopping the reaction after 2 min. The type of inhibition, *K*_m,_ and V_max_ values were determined from the plots. Slopes and Y-intercepts of these reciprocal plots were also replotted against the inhibitor concentration, respectively. Data analysis was performed by the Prism software.

### 3.6. Circular Dichroism Measurement of Inhibitor-Enzyme Complex

CD spectra (190–250 nm) of α-glucosidase with and without compound **7** were recorded on a Chirascan CD spectrometer at room temperature. All the CD spectra were corrected with the buffer signal under constant nitrogen flush. The concentration of α-glucosidase was 2.0 μM, and the molar ratios of compound **7** to α-glucosidase were set to 0:1, 1:1, and 2:1.

## 4. Conclusions

In summary, we use a biomimetic hetero-Diels-Alder reaction as a tool for generating NP-like skeletons. Although the yields and stereoselectivities of these reactions were very limited, the product diversity provided a chance to explore new NP-like scaffolds. These reactions starting from 4-hydroxycoumarins and β-caryophyllene could provide a facile way to acquire meroterpene-like skeletons. All 17 products, representing seven different scaffolds, provided candidates to identify potentially new chemotypes. Due to the limited stereoselectivity of the construction reaction, all the ACs of products were determined unambiguously by ECD calculations. Inspired by the inhibition of compound **5a**, we furtherly linked β-caryophyllene and lawsone (or syncarpic acid) via an ether connection to yield compound **6** and **7**. Compounds **5a**, **6**, and **7** showed interesting potential activities (IC_50_ < 10 μM). They can be considered novel scaffolds for the design of α-glucosidase inhibitors. Furthermore, side products from biomimetic reactions can provide diverse NP-like scaffolds, and will favor searching for lead candidates for drug discovery. Since a hydrogen bonding site is essential for inhibition, we will further explore the chemical space in the future based on natural polyphenol moieties. Also, the inhibition mechanism should be further investigated based on a more promising molecule.

## Figures and Tables

**Table 1:** α-Glucosidase (EC 3.2.1.20) inhibition of highly active compounds in vitro.

Compd.	IC_50_ (μM)	Compd.	IC_50_ (μM)
**2b**	15.56 ± 0.31	**5a**	9.49 ± 0.03
**2c**	23.62 ± 1.69	**5b**	11.81 ± 0.03
**3b**	12.04 ± 0.06	**5c**	12.04 ± 0.10
**3c**	18.09 ± 0.05	**6**	8.50 ± 0.24
**4b**	15.35 ± 0.33	**7**	3.56 ± 0.24
**4c**	19.99 ± 0.47	**G**	58.19 ± 1.38
		**A**	54.74 ± 0.16

All the other synthesized compounds and starting materials showed weak inhibition (IC_50_ > 25 μM). G, genistein. A, acarbose.

## Supplementary Materials

The following are available online. Figure S1: GC-EI MS and LC-ESI-MS/MS analysis, Figure S2: NMR and HR-MS spectra.
