# Supplementary material for: Meroterpene-Like α-Glucosidase Inhibitors Based on Biomimetic Reactions Starting from β-Caryophyllene
Source: Molecules. 2020 Jan 8;25(2):260. doi: 10.3390/molecules25020260 (PMC7024386; doi:10.3390/molecules25020260)
Supplement: Supplementary file 1 [file molecules-25-00260-s001.pdf]

Meroterpene-like α-glucosidase inhibitors based on biomimic reactions staring from β-caryophyllene

Da-Wei Yan[1], Cheng-Di Huang[1], Hang-Hang Zheng[1], Na Zhao, Shuang-Jiang Ma[1], Xiao-Lan Feng[2], Jin-Ming Gao[1,*] and Qiang Zhang[1,*]

1 Shaanxi Key Laboratory of Natural Products & Chemical Biology, College of Chemistry & Pharmacy, Northwest A&F University, Yangling 712100, PR China; 15829769577@163.com (D.-W.Y.), chengdihuang@nwafu.edu.cn (C.-D.H.), zhh18710683097@163.com (H.-H.Z.), 1984162639@nwafu.edu.cn (N.Z.), tianjijuechen00@163.com (S.-J.M.)

2 College of veterinary medicine, Northwest A&F University, Yangling 712100, PR China, xiaolanfeng@nwafu.edu.cn

* Correspondence: jinminggao@nwsuaf.edu.cn (J.-M.G.), zhangq@nwsuaf.edu.cn (Q.Z.); Tel.: +86-0298-709-2335 (J.-M.G.), +86-1809-272-0670 (Q.Z.)

Content

Figure S1. GC-EI MS (a) and LC-ESI-MS/MS analysis 2

Figure S2: NMR and HR-MS spectra 4

- Compound 1a 4
- Compound 2a 7
- Compound 3a 10
- Compound 4a 13
- Compound 5a 16
- Compound 1b 23
- Compound 2b 26
- Compound 3b 29
- Compound 4b 32
- Compound 5b 35
- Compound 1c 38
- Compound 2c 41
- Compound 3c 44
- Compound 4c 47
- Compound 5c 50
- Compound 6 53
- Compound 7 60

Figure S1. GC-EI MS (a) and LC-ESI-MS/MS analysis

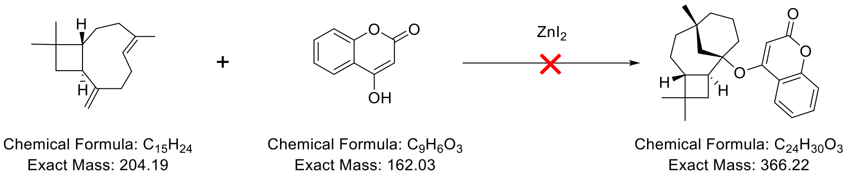

(a) GC-EI-MS analysis

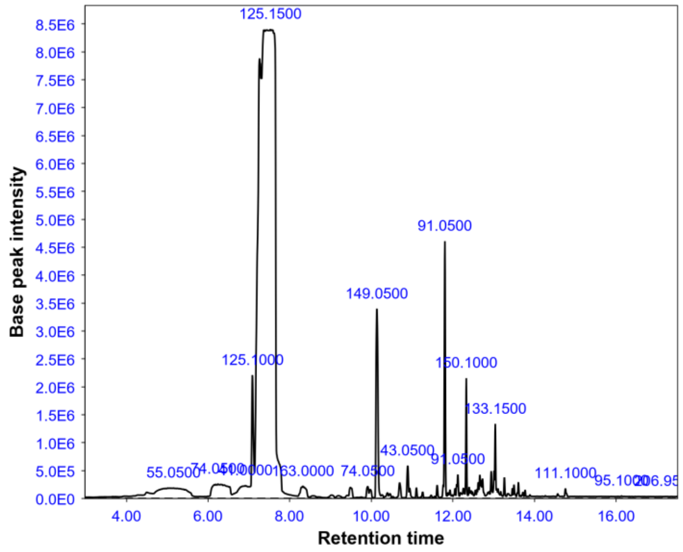

The crude product was dissolved in MeOH (200 µg/ml) and was analyzed on a Shimadzu GCMS-QP2010 Ultra instrument equipped with a capillary column of Rxi® -5Sil MS (30 m × 0.25 mm i.d., 0.25 µm film thickness). Helium (He) gas was used as a mobile phase at a constant flow of 1.3 mL/min. The temperature was held at 150 °C for 5 min, then increased to 300 at 4 °C/min, maintaining the last temperature for 5 min. The temperature of the ion source was 250 °C. EI-MS spectra (*m/z* 35 ~ 500) were recorded with a scan event time of 0.3 s. Electron energy was 70 eV. The raw data was saved into netCDF format in the GCMS solution software and then was converted into mzXML format by ProteoWizard MSConvert 3. The TIC chromatography was extracted using MZMine2.

(b) LC-QTOF-MS/MS analysis

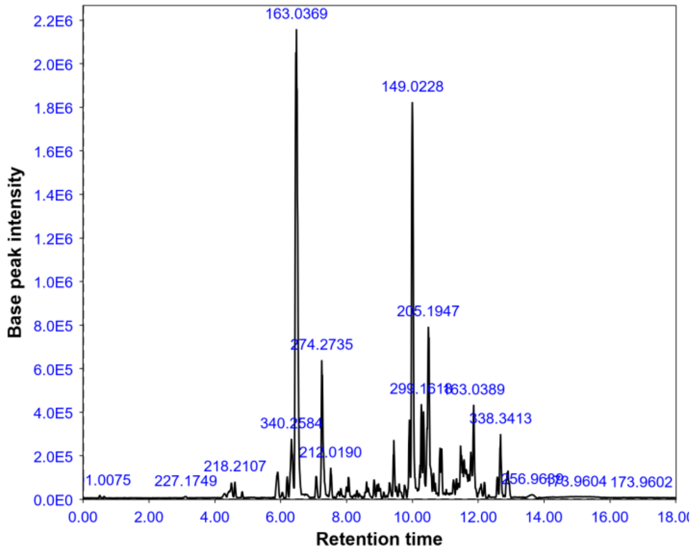

The crude product was analyzed by AB SCIEX TripleTOF5600+ equipped with a Shimadzu UPLC (LC-30A）Shim-pack XR-ODS (100 mm × 2.1 mm, 1.7 μ m) column. The temperature was maintained at 30 ° C. Mixtures of H_2O (A), and MeOH (B) were eluted at the flow rate of 0.3 mL/min with a linear gradient of 10 ~ 100% B (0 − 17 min). The samples (200 µg/mL in MeOH, 2 μ L portions were injected) were analyzed in data-dependent acquisition (DDA) mode. The full MS survey scan was performed for 150 ms in the range of *m/z* 100 ~ 2000; The collision energy was set as 40 eV. The raw data were converted to the mzXML format with MSConvert 3.0. The TIC chromatography was extracted using MZMine2 with *m/z* range 100~1500.

Figure S2: NMR and HR-MS spectra

Compound 1a

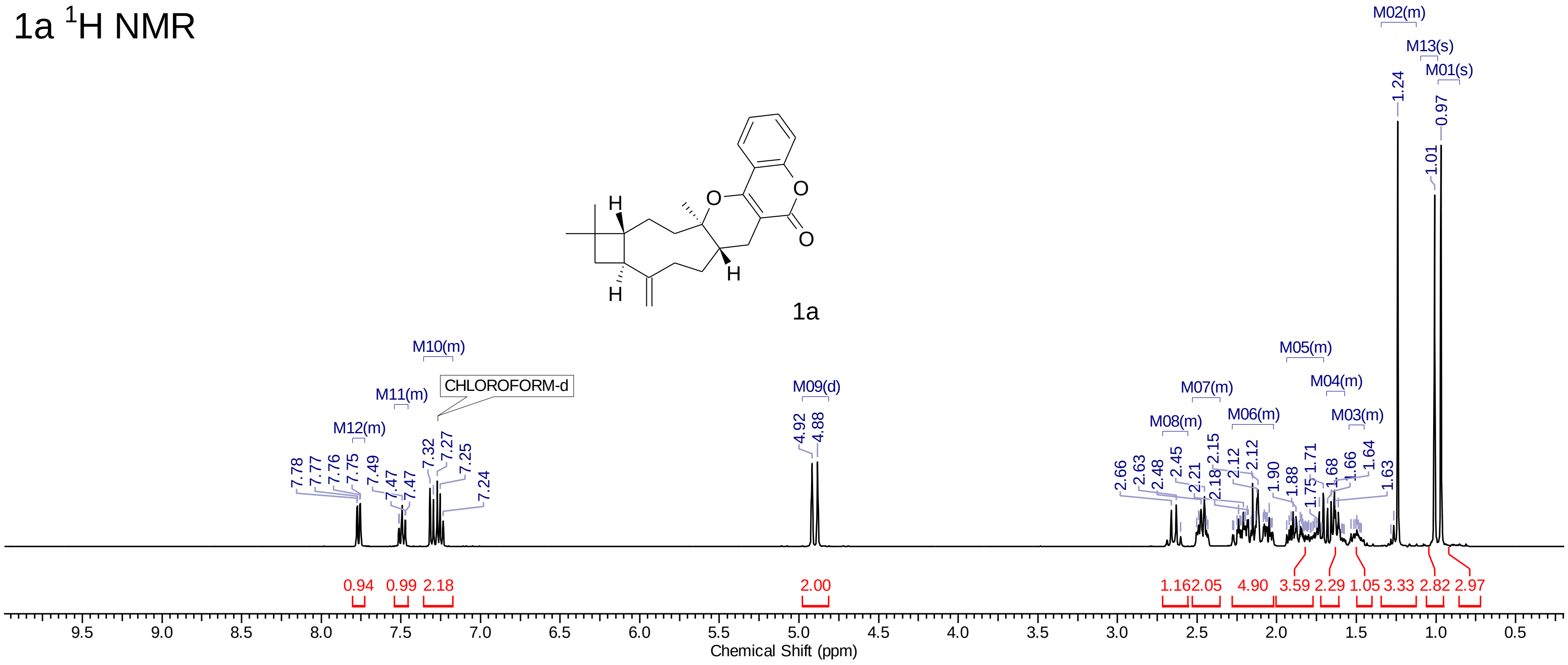

DEPT135 and ^{13}C NMR

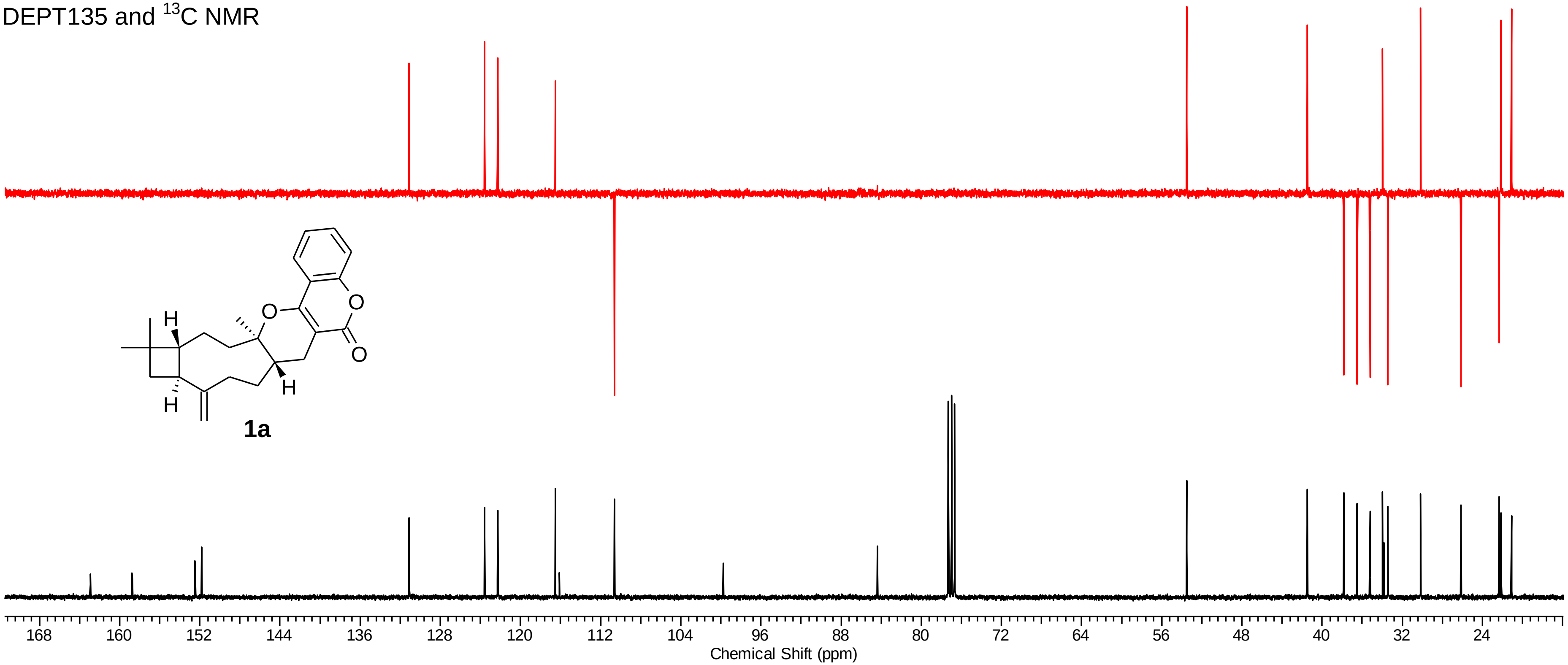

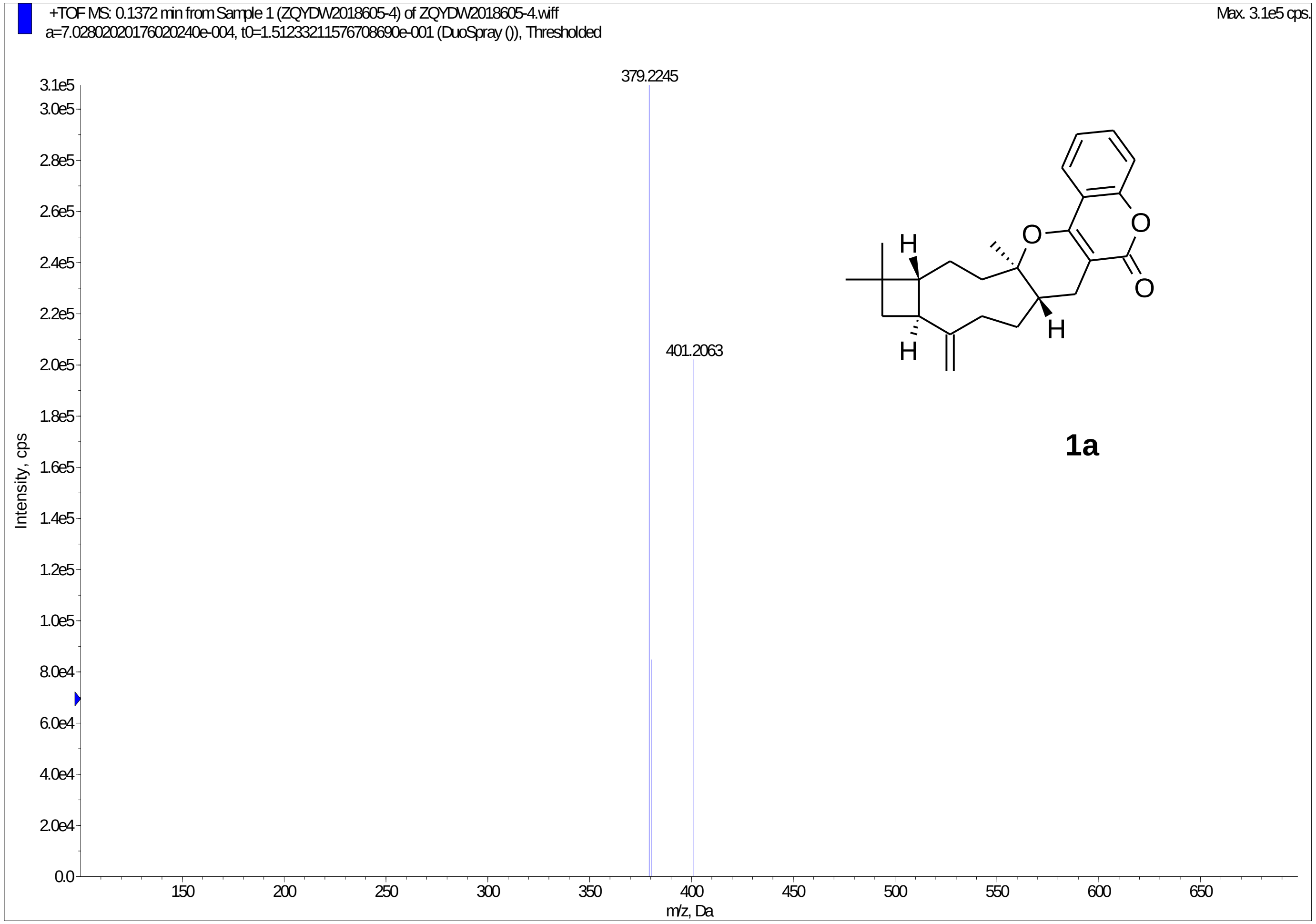
+TOF MS: 0.1372 min from Sample 1 (ZQYDW2018605-4) of ZQYDW2018605-4.wiff
a=7.02802020176020240e-004, t0=1.51233211576708690e-001 (DuoSpray ()), Thresholded
Max. 3.1e5 cps.
379.2245
401.2063
Intensity, cps
m/z, Da
1a

Compound 2a

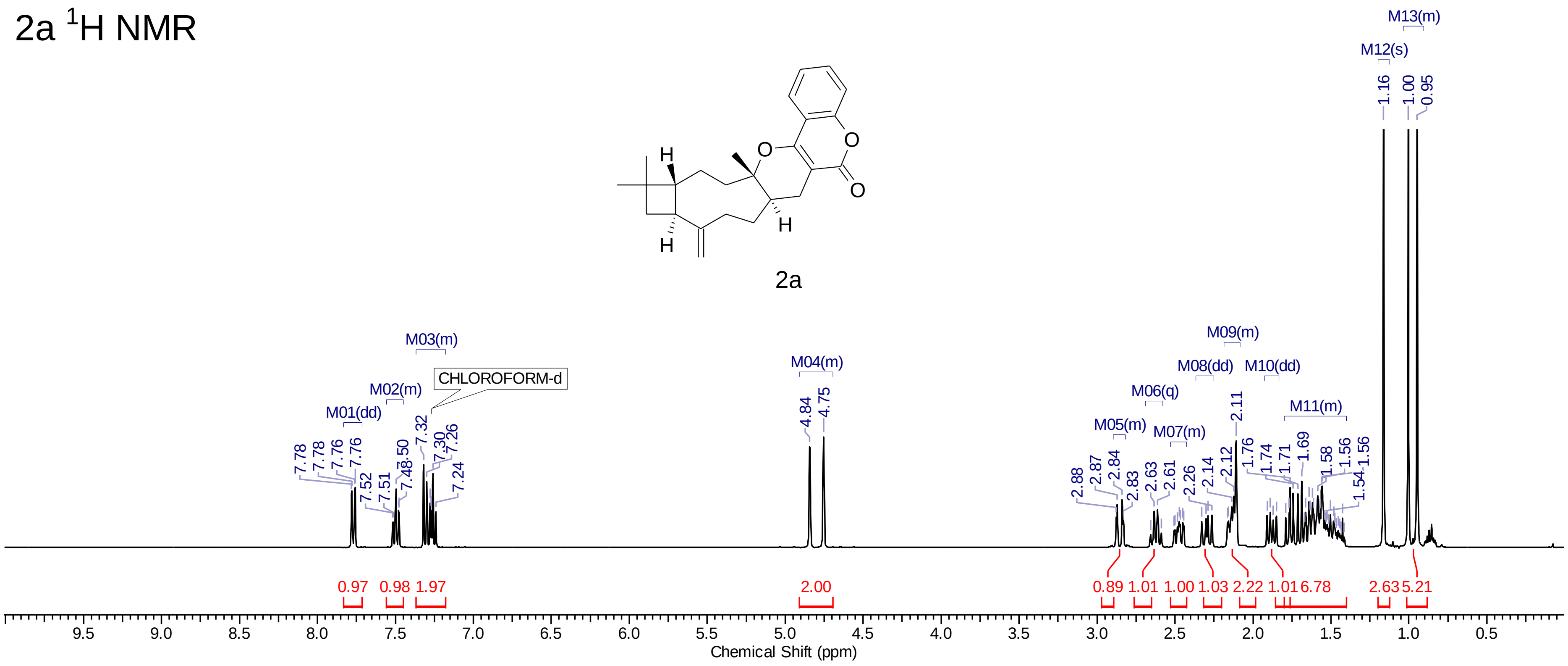
2a ^{1}H NMR
2a
CHLOROFORM-d
M01(dd)
M02(m)
M03(m)
M04(m)
M05(m)
M06(q)
M07(m)
M08(dd)
M09(m)
M10(dd)
M11(m)
M12(s)
M13(m)
0.97 0.98 1.97 2.00 0.89 1.01 1.00 1.03 2.22 1.01 6.78 2.63 5.21
Chemical Shift (ppm)

2a ^{13}C NMR

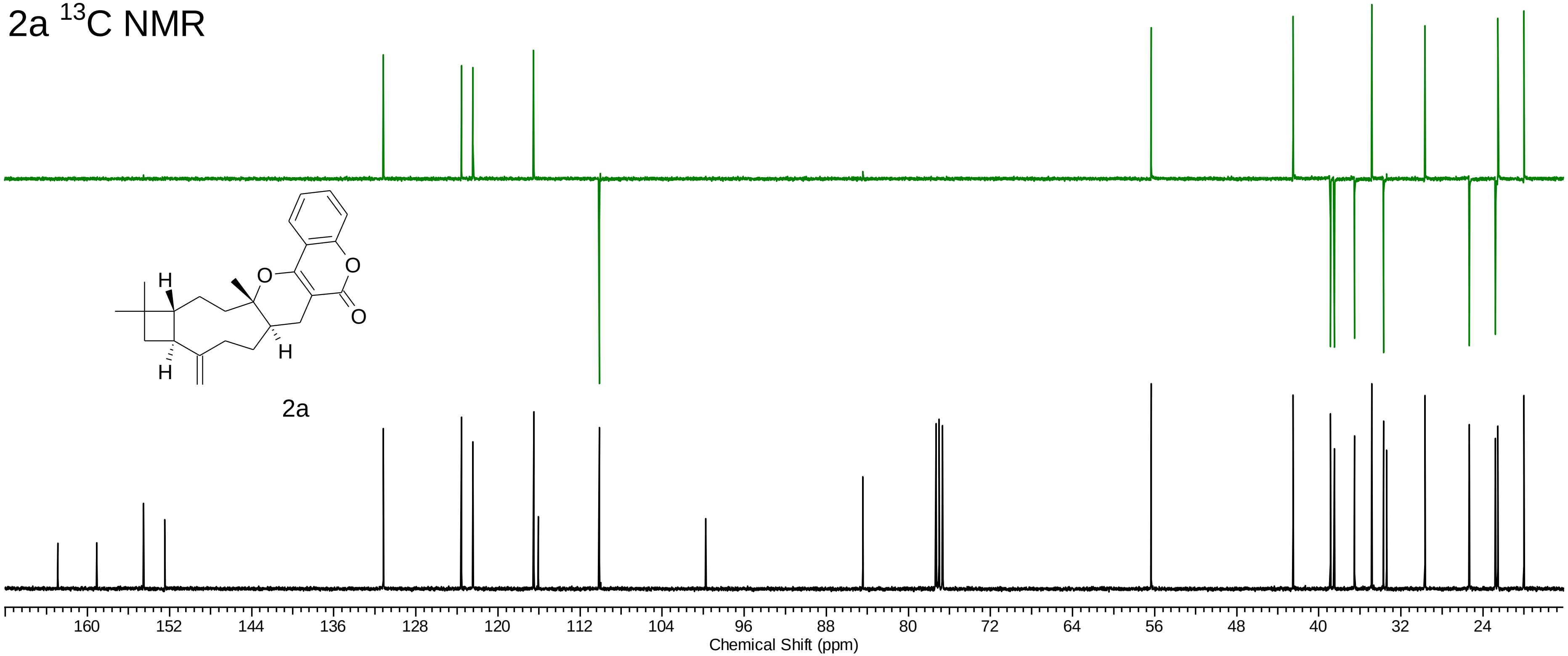

H
O
O
O
H
H
2a
160
152
144
136
128
120
112
104
96
88
80
72
64
56
48
40
32
24
Chemical Shift (ppm)

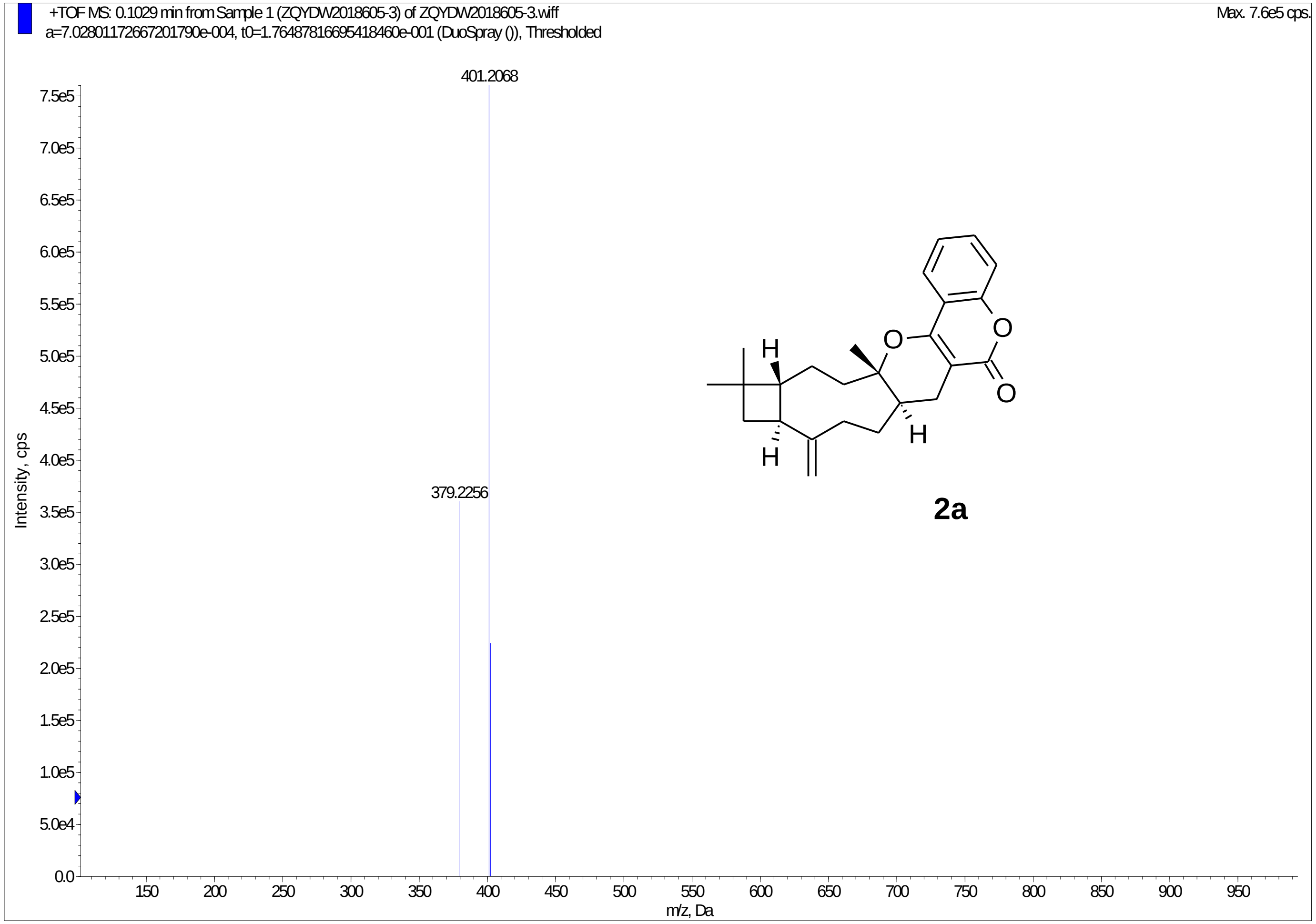
+TOF MS: 0.1029 min from Sample 1 (ZQYDW2018605-3) of ZQYDW2018605-3.wiff
a=7.02801172667201790e-004, t0=1.76487816695418460e-001 (DuoSpray ()), Thresholded
Max. 7.6e5 cps.
401.2068
379.2256
Intensity, cps
m/z, Da
2a

Compound 3a

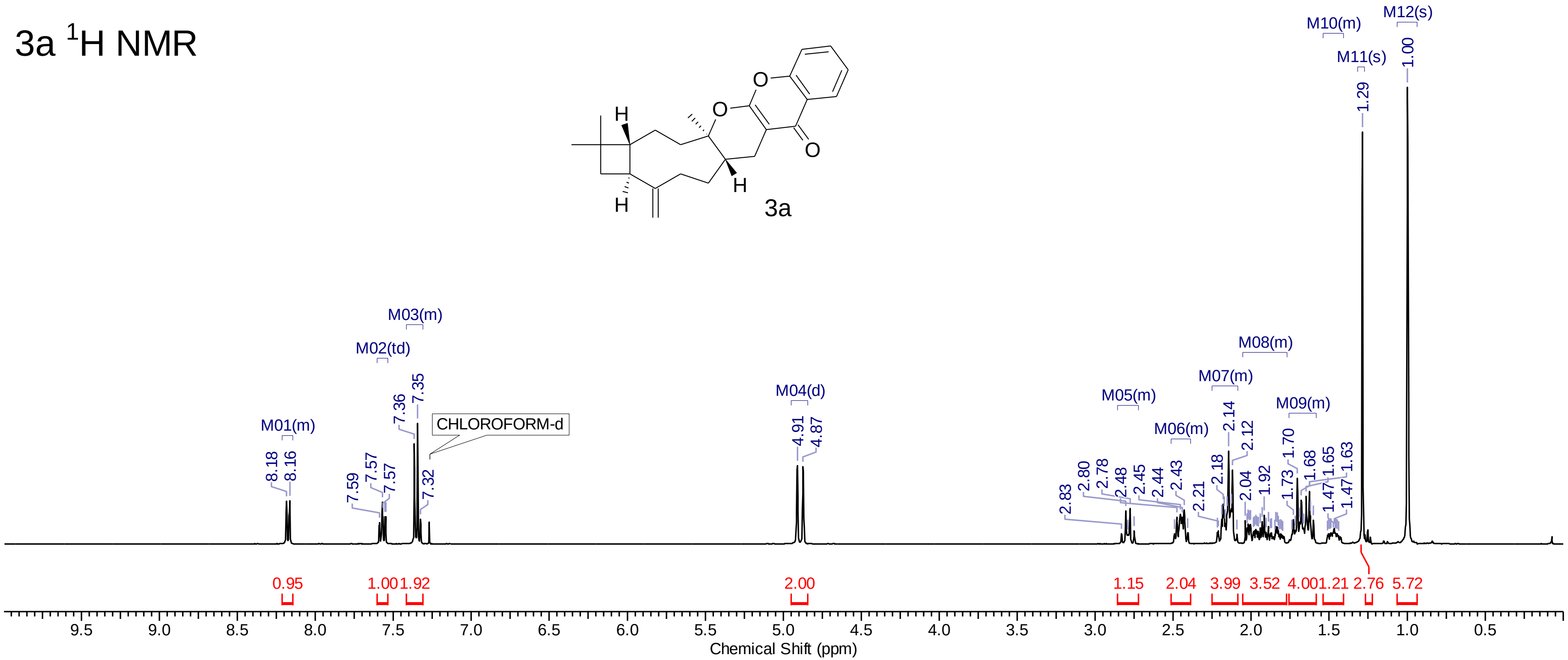
3a ^{1}H NMR
3a
M01(m)
M02(td)
M03(m)
M04(d)
M05(m)
M06(m)
M07(m)
M08(m)
M09(m)
M10(m)
M11(s)
M12(s)
CHLOROFORM-d
Chemical Shift (ppm)

DEPT135 and ^{13}C NMR

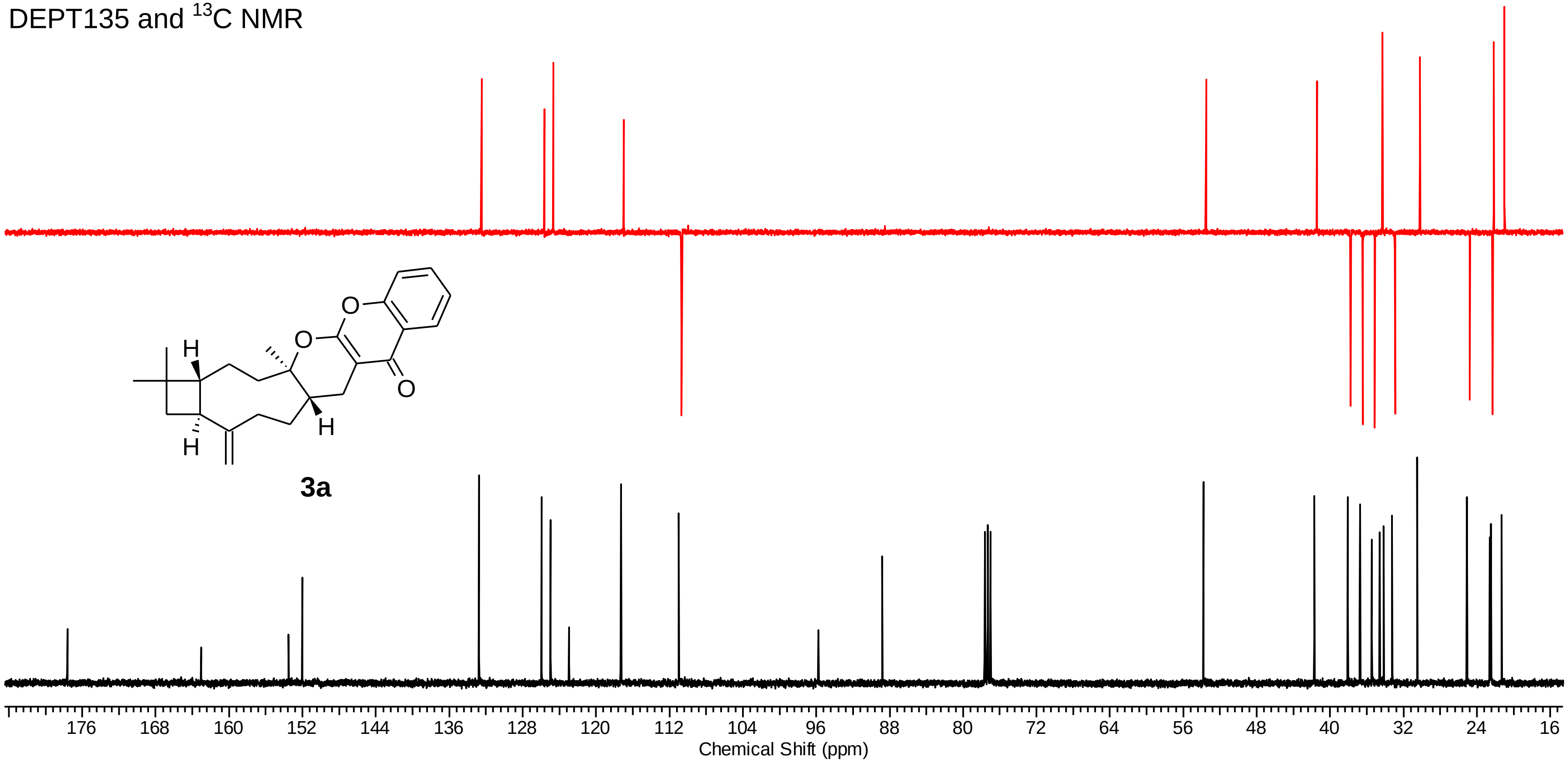

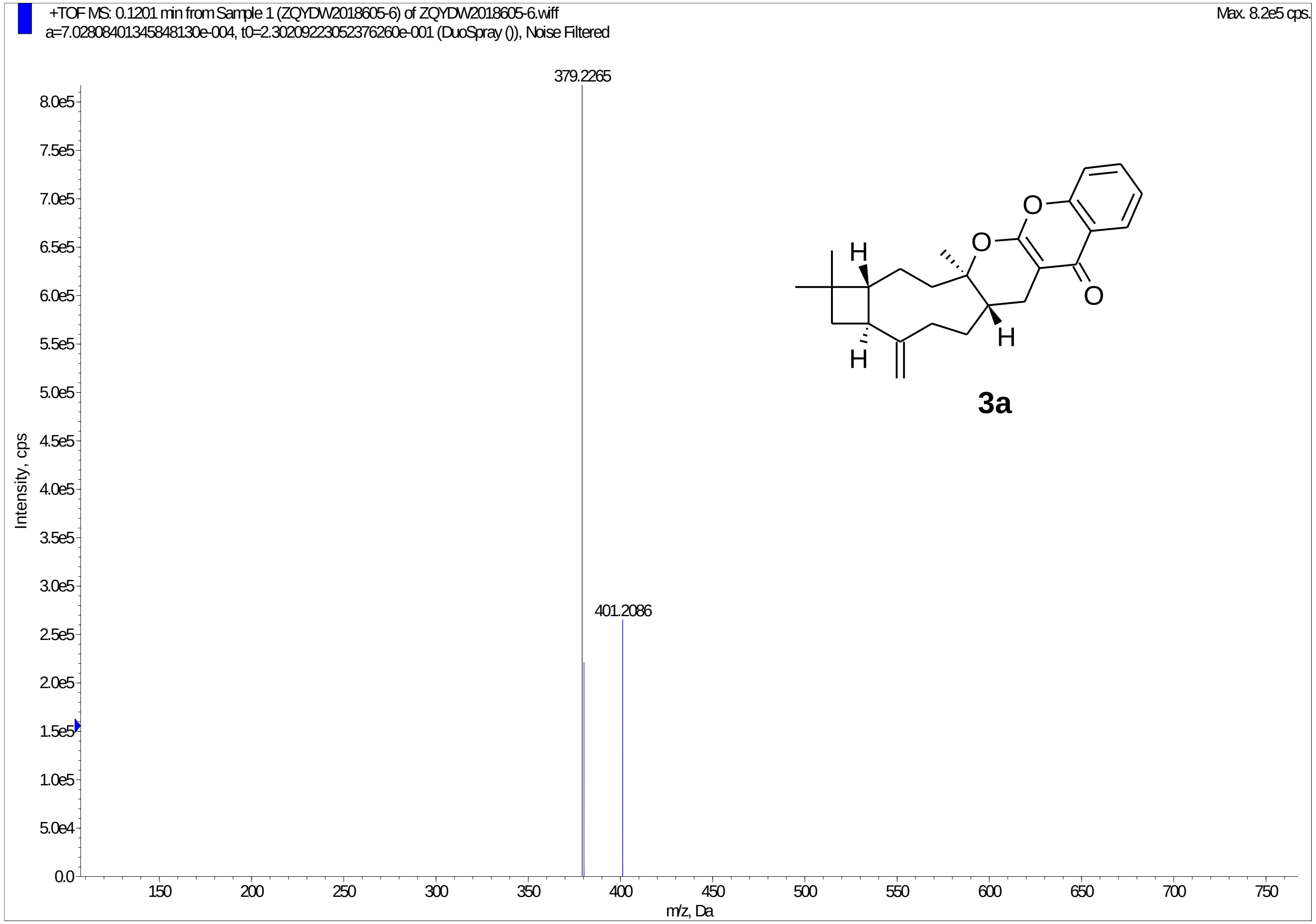
+TOF MS: 0.1201 min from Sample 1 (ZQYDW2018605-6) of ZQYDW2018605-6.wiff
a=7.02808401345848130e-004, t0=2.30209223052376260e-001 (DuoSpray ()), Noise Filtered
Max. 8.2e5 cps.
379.2265
401.2086
Intensity, cps
m/z, Da
3a

Compound 4a

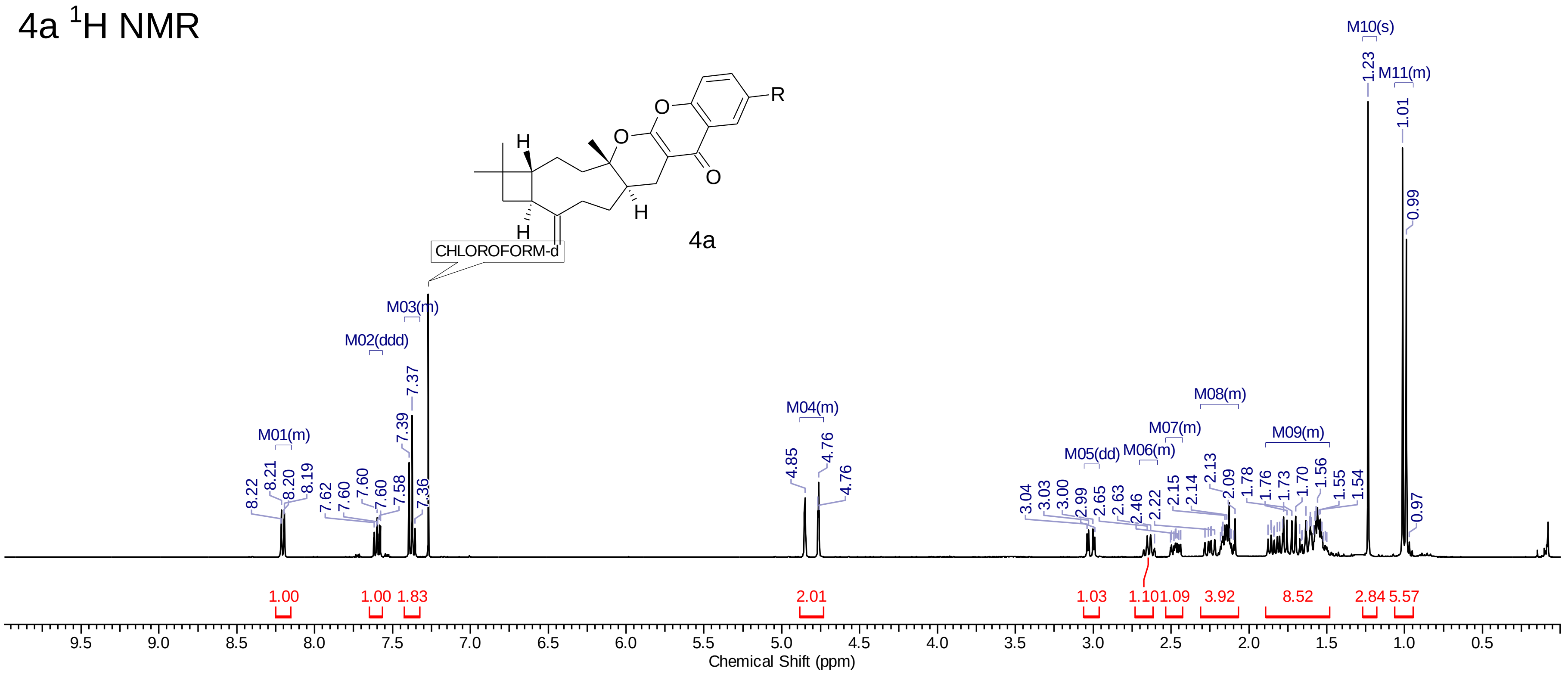
4a ¹H NMR
4a
CHLOROFORM-d
M01(m)
M02(ddd)
M03(m)
M04(m)
M05(dd)
M06(m)
M07(m)
M08(m)
M09(m)
M10(s)
M11(m)
1.00 1.00 1.83 2.01 1.03 1.10 1.09 3.92 8.52 2.84 5.57
Chemical Shift (ppm)

DEPT135 and ^{13}C NMR

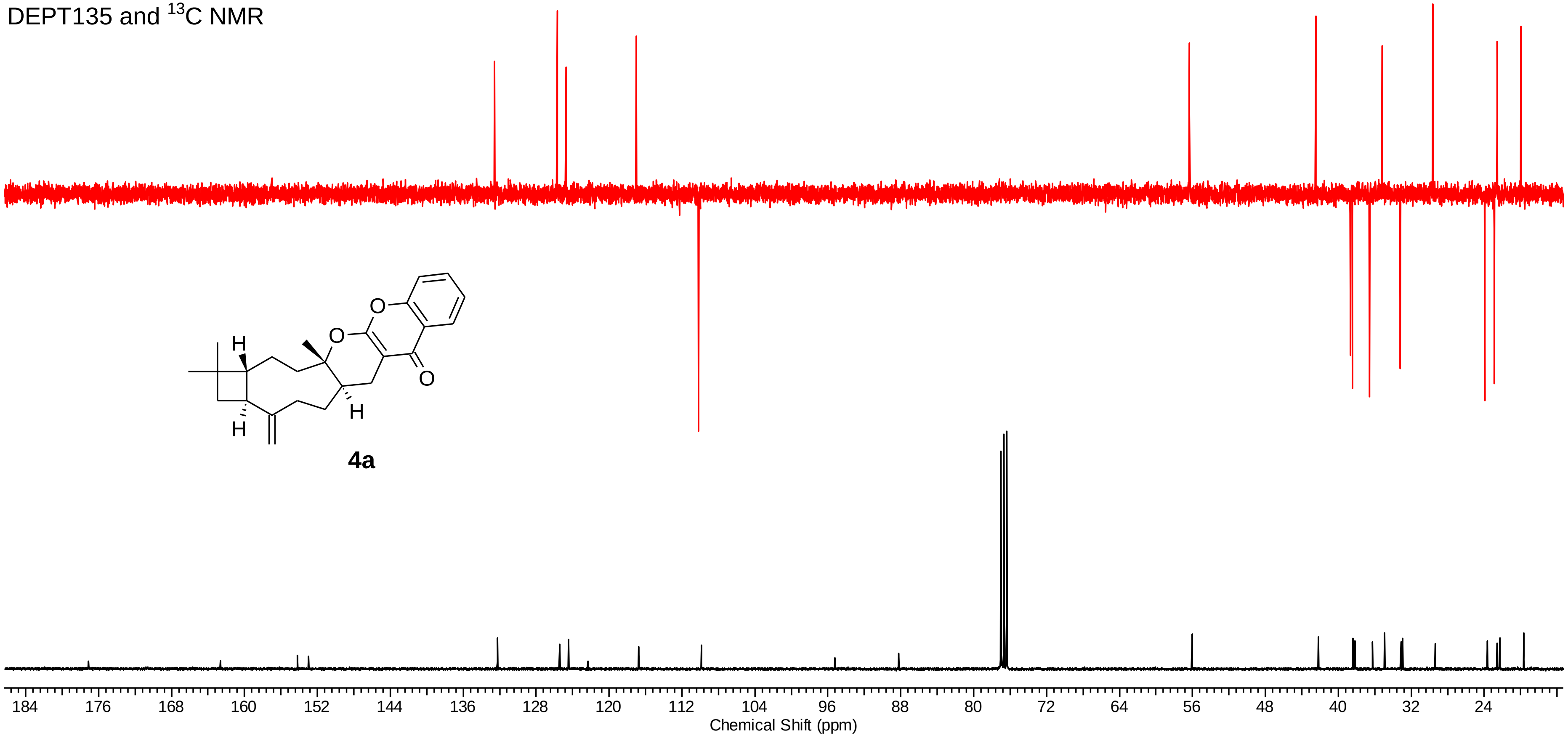

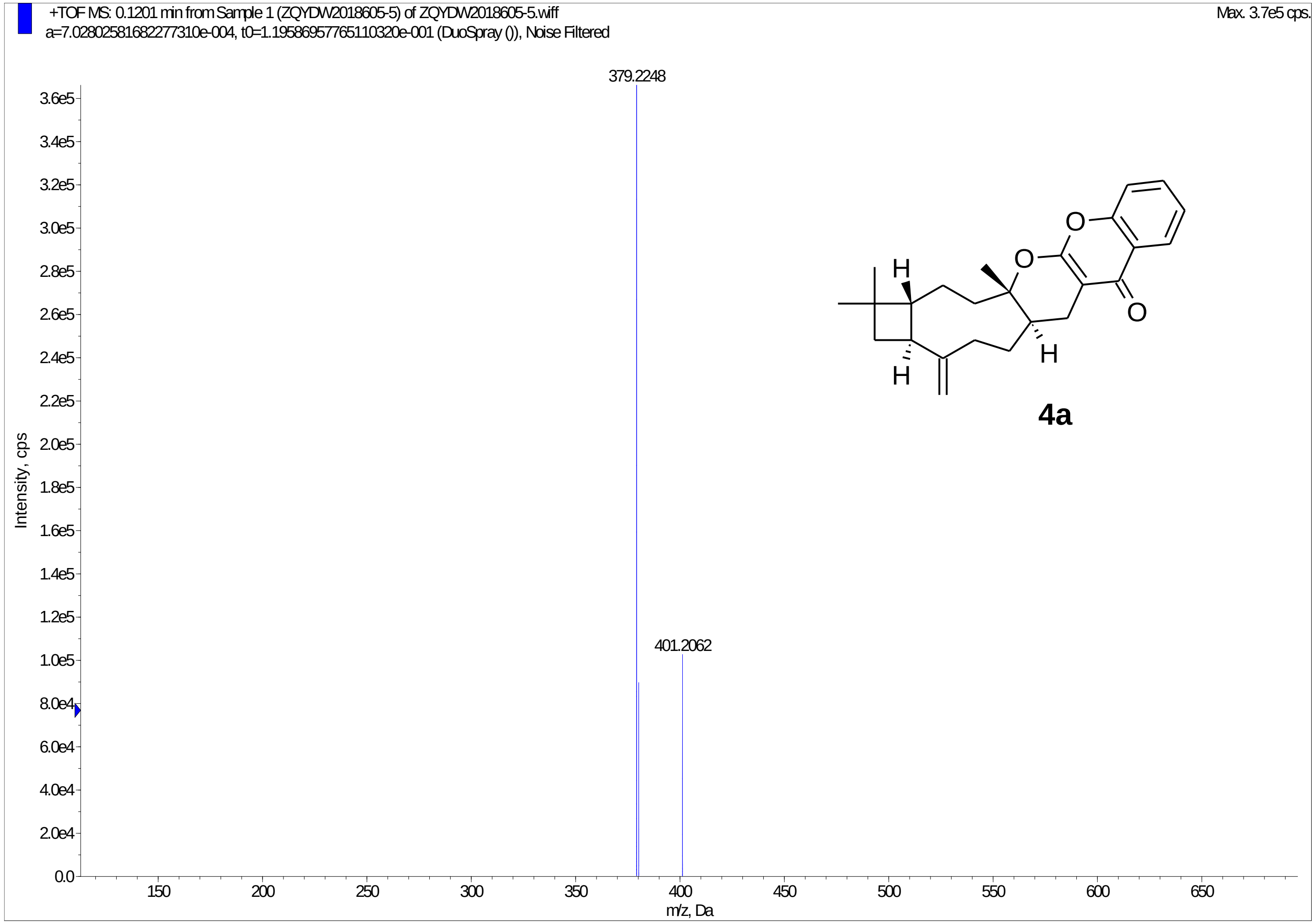
+TOF MS: 0.1201 min from Sample 1 (ZQYDW2018605-5) of ZQYDW2018605-5.wiff
a=7.02802581682277310e-004, t0=1.19586957765110320e-001 (DuoSpray ()), Noise Filtered
Max. 3.7e5 cps.
379.2248
401.2062
Intensity, cps
m/z, Da
H
H
H
O
O
O
4a

Compound 5a

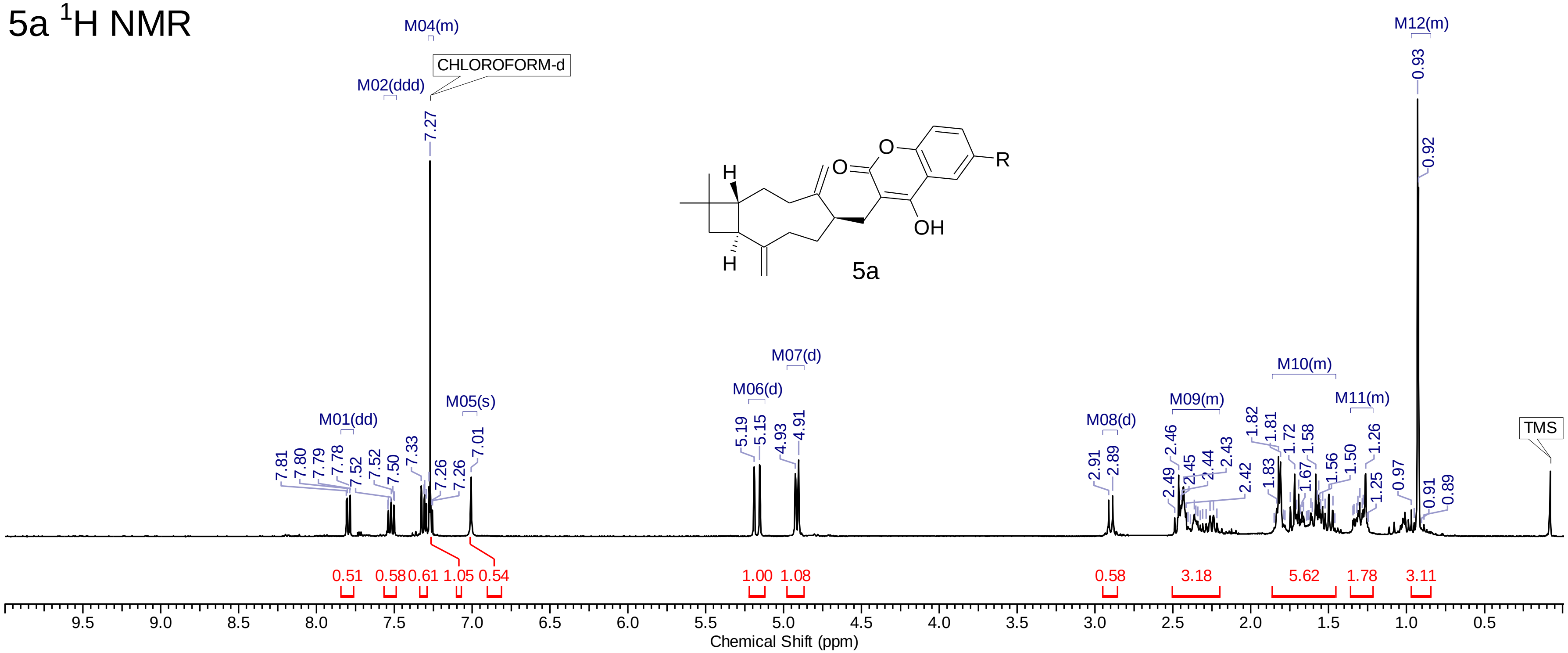
5a ¹H NMR
M01(dd)
M02(ddd)
M04(m)
CHLOROFORM-d
M05(s)
M06(d)
M07(d)
M08(d)
M09(m)
M10(m)
M11(m)
M12(m)
TMS
5a
OH
R
0.51
0.58
0.61
1.05
0.54
1.00
1.08
0.58
3.18
5.62
1.78
3.11
Chemical Shift (ppm)

DEPT135 and ^{13}C NMR

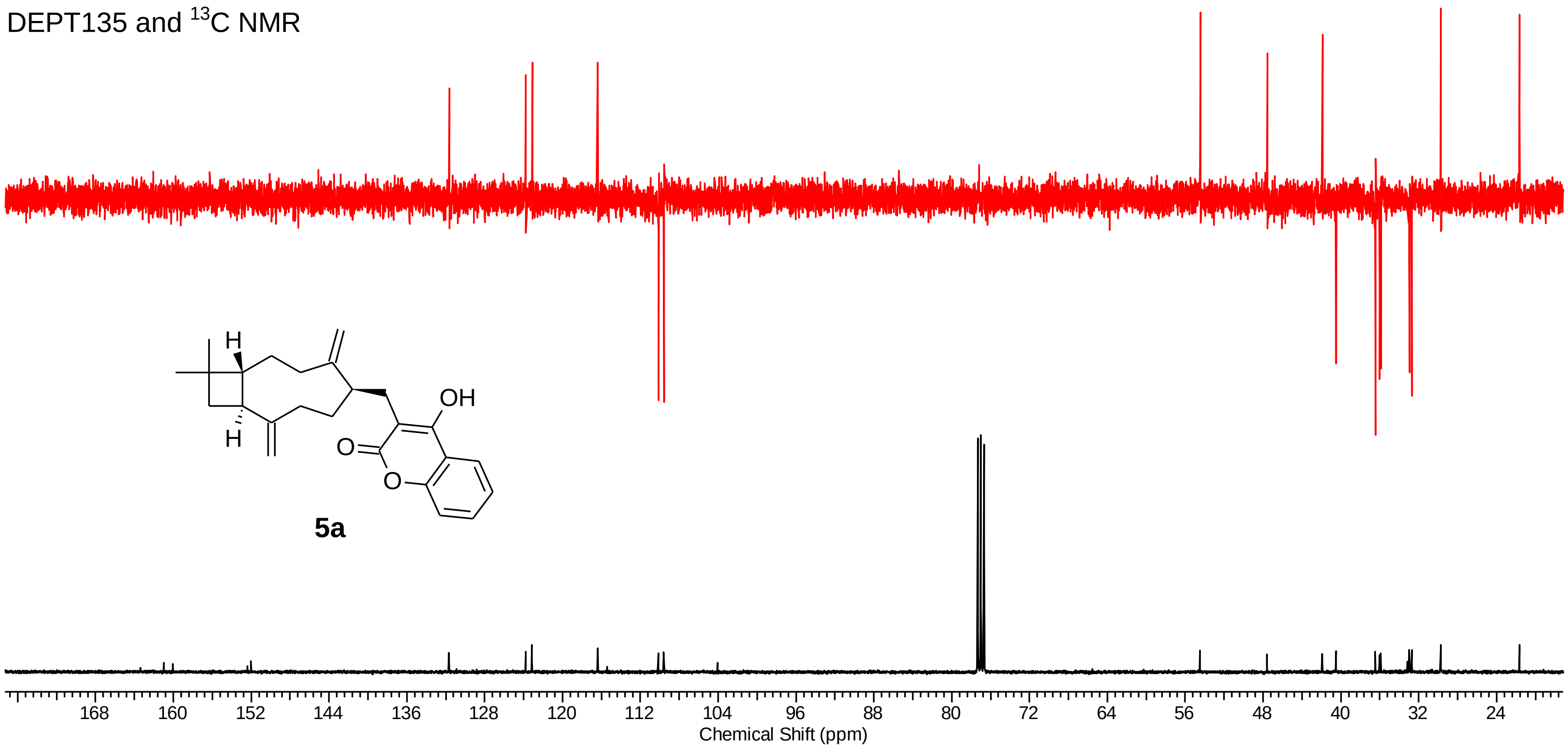

5a HSQC

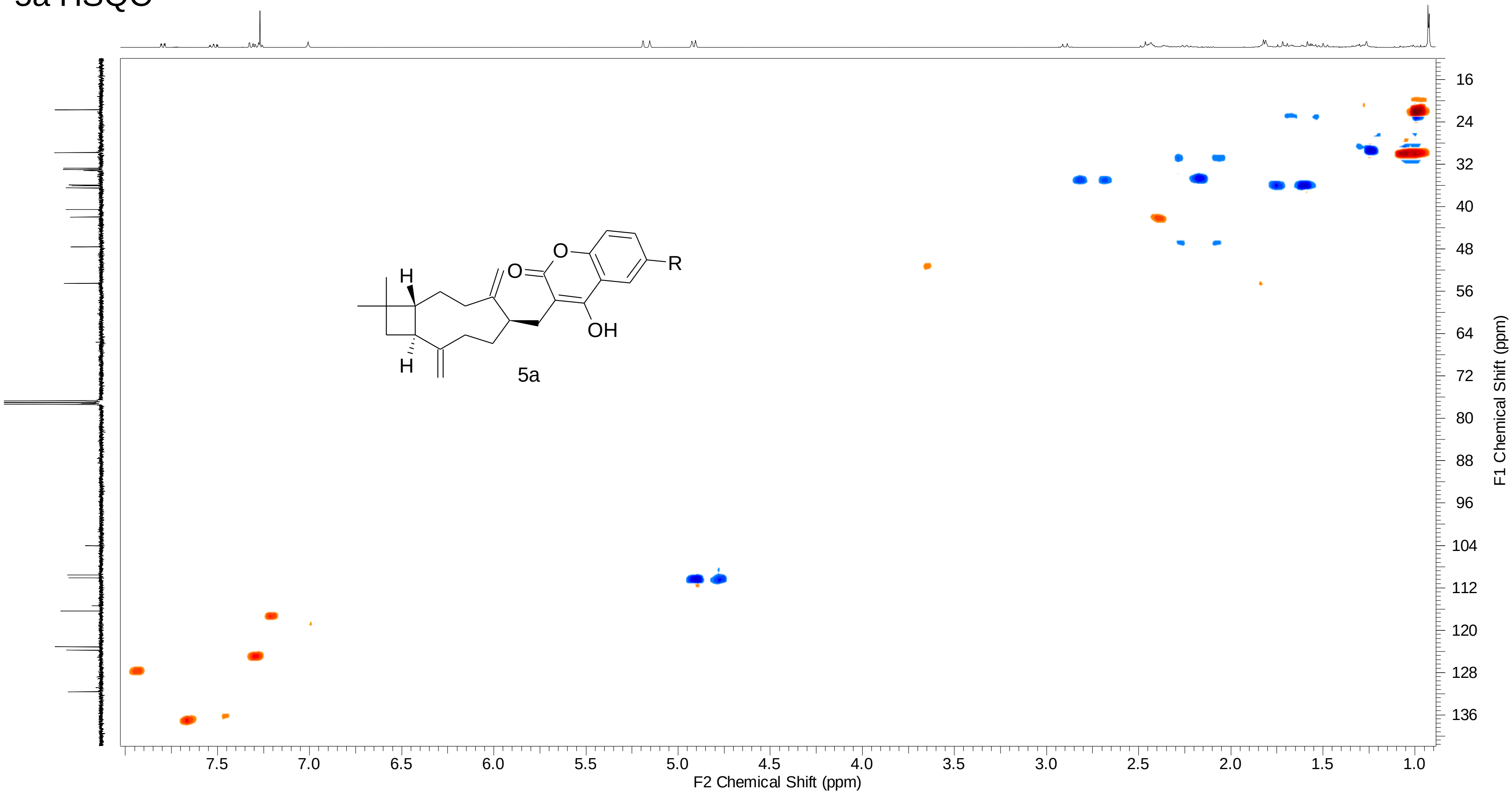

5a COSY

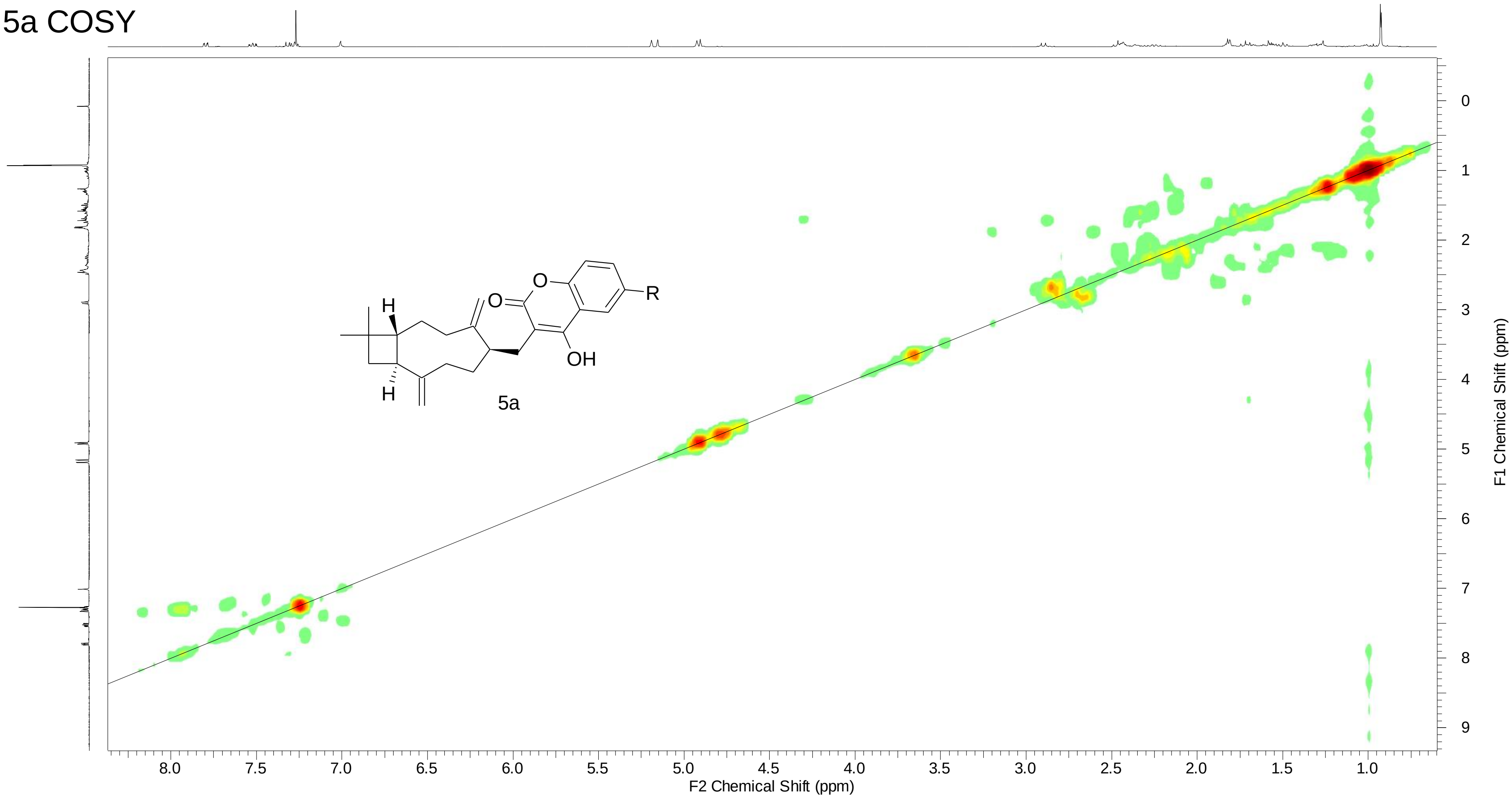

H
H
O
O
R
OH
5a
F2 Chemical Shift (ppm)
F1 Chemical Shift (ppm)
8.0
7.5
7.0
6.5
6.0
5.5
5.0
4.5
4.0
3.5
3.0
2.5
2.0
1.5
1.0
0
1
2
3
4
5
6
7
8
9

5a HMBC

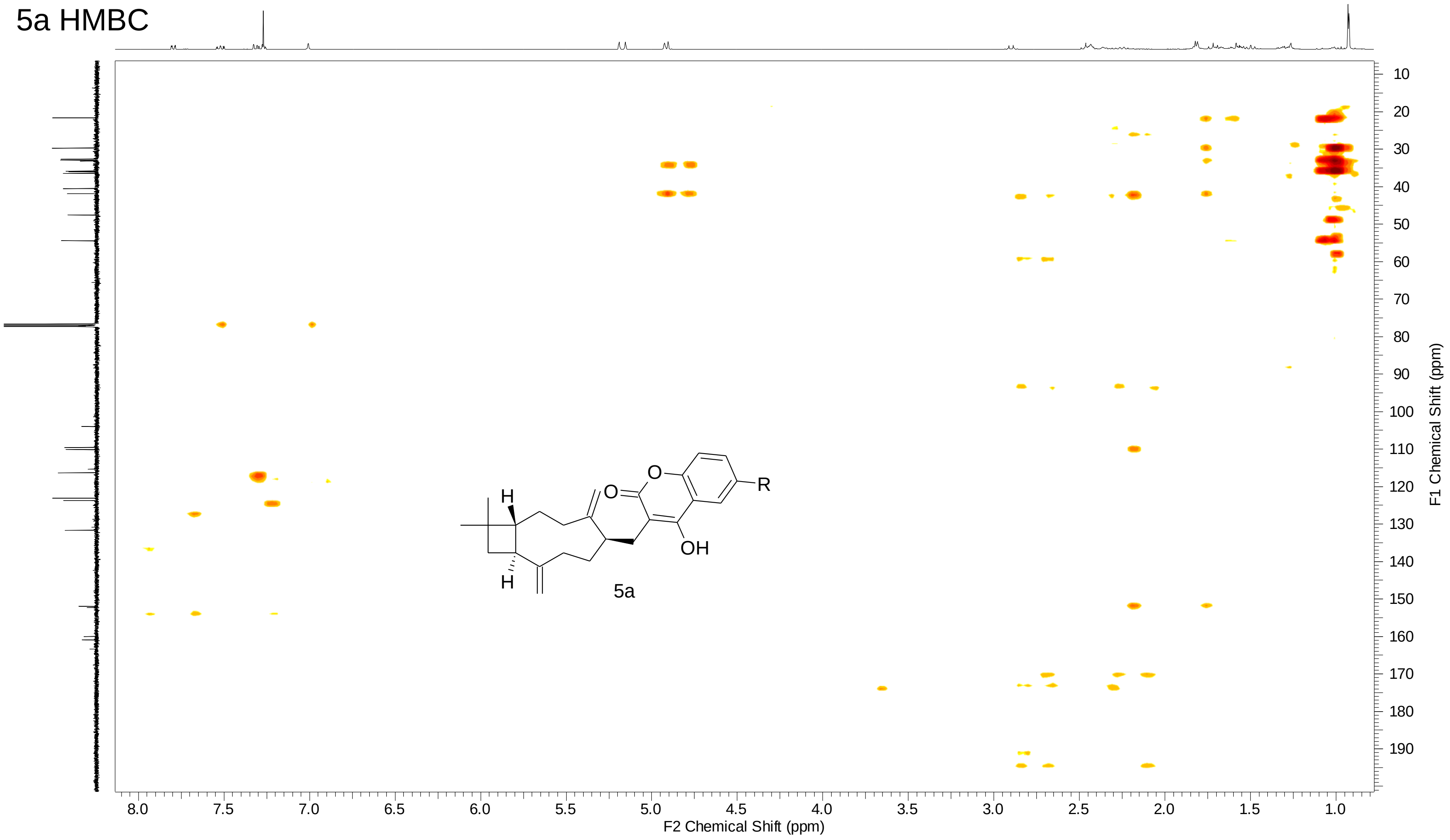

5a NOESY

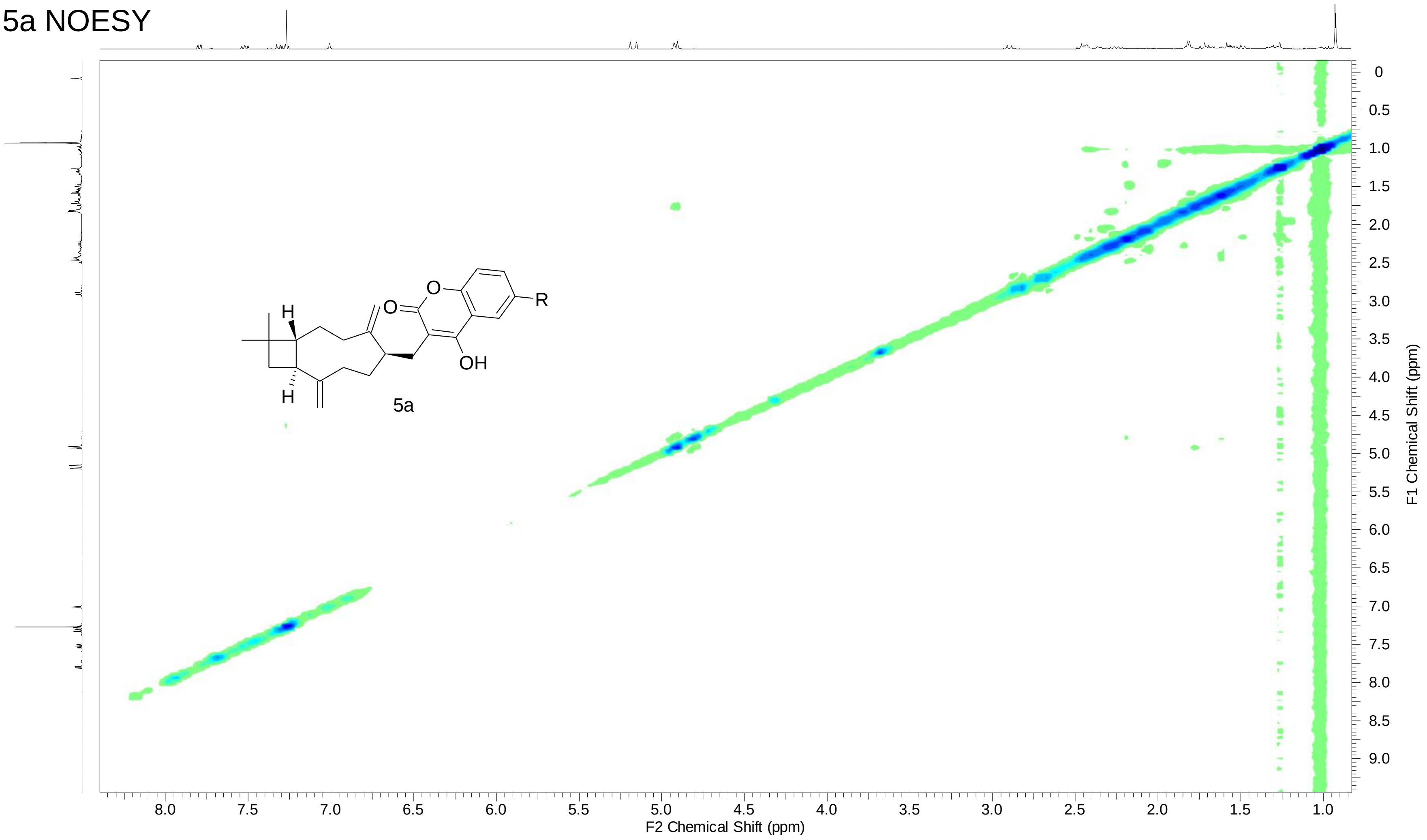
F2 Chemical Shift (ppm)
F1 Chemical Shift (ppm)
8.0
7.5
7.0
6.5
6.0
5.5
5.0
4.5
4.0
3.5
3.0
2.5
2.0
1.5
1.0
0
0.5
9.0
H
H
O
O
R
OH
5a

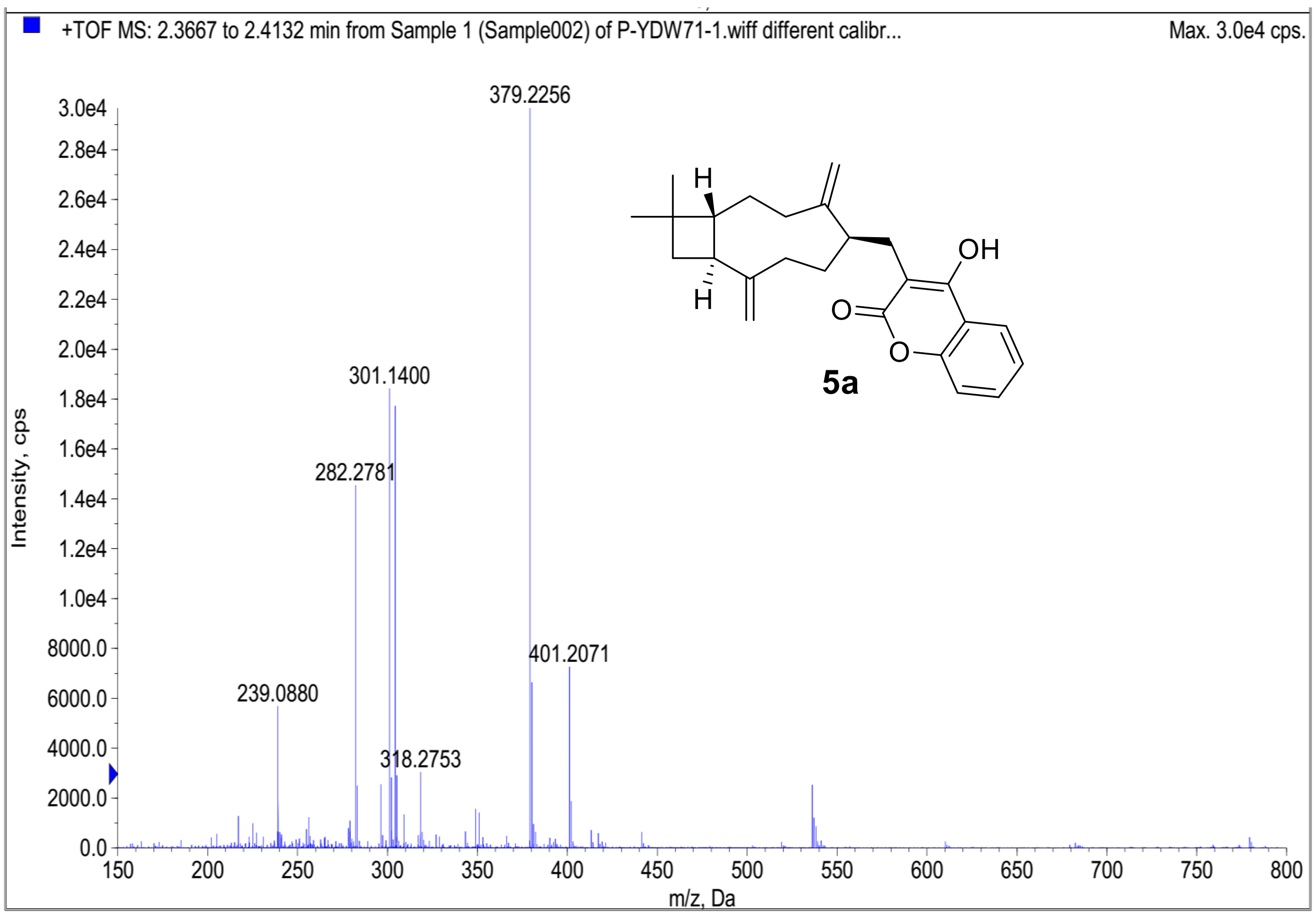
+TOF MS: 2.3667 to 2.4132 min from Sample 1 (Sample002) of P-YDW71-1.wiff different calibr...
Max. 3.0e4 cps.
379.2256
301.1400
282.2781
401.2071
239.0880
318.2753
Intensity, cps
m/z, Da
OH
O
O
H
H
5a

Compound 1b

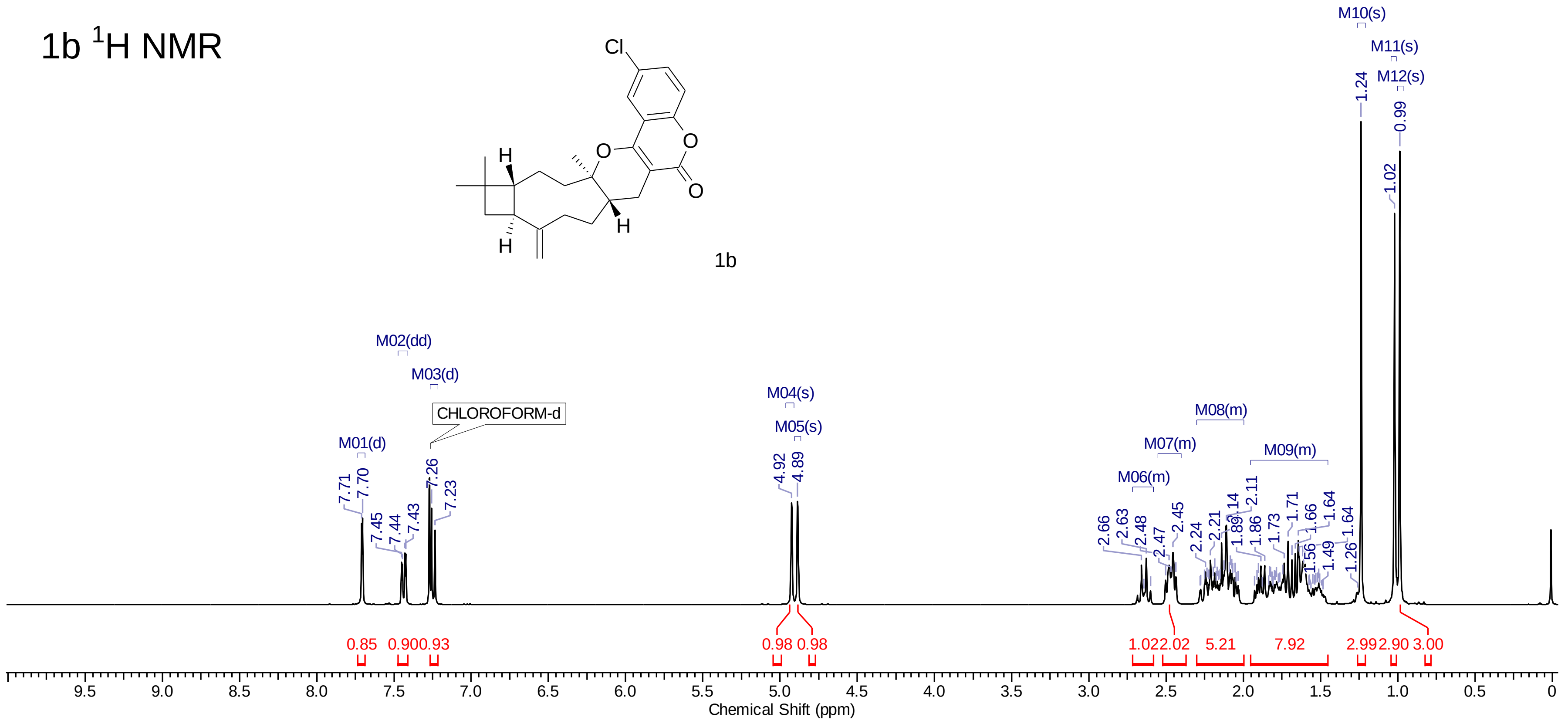
1b ^{1}H NMR
Cl
O
O
O
H
H
H
1b
M01(d)
M02(dd)
M03(d)
CHLOROFORM-d
M04(s)
M05(s)
M06(m)
M07(m)
M08(m)
M09(m)
M10(s)
M11(s)
M12(s)
7.71
7.70
7.45
7.44
7.43
7.26
7.23
4.92
4.89
2.66
2.63
2.48
2.47
2.45
2.24
2.21
2.14
2.11
1.89
1.86
1.73
1.71
1.66
1.64
1.64
1.56
1.49
1.26
1.24
1.02
0.99
0.85
0.90
0.93
0.98
0.98
1.02
2.02
5.21
7.92
2.99
2.90
3.00
9.5
9.0
8.5
8.0
7.5
7.0
6.5
6.0
5.5
5.0
4.5
4.0
3.5
3.0
2.5
2.0
1.5
1.0
0.5
0
Chemical Shift (ppm)

DEPT135 and 13C NMR

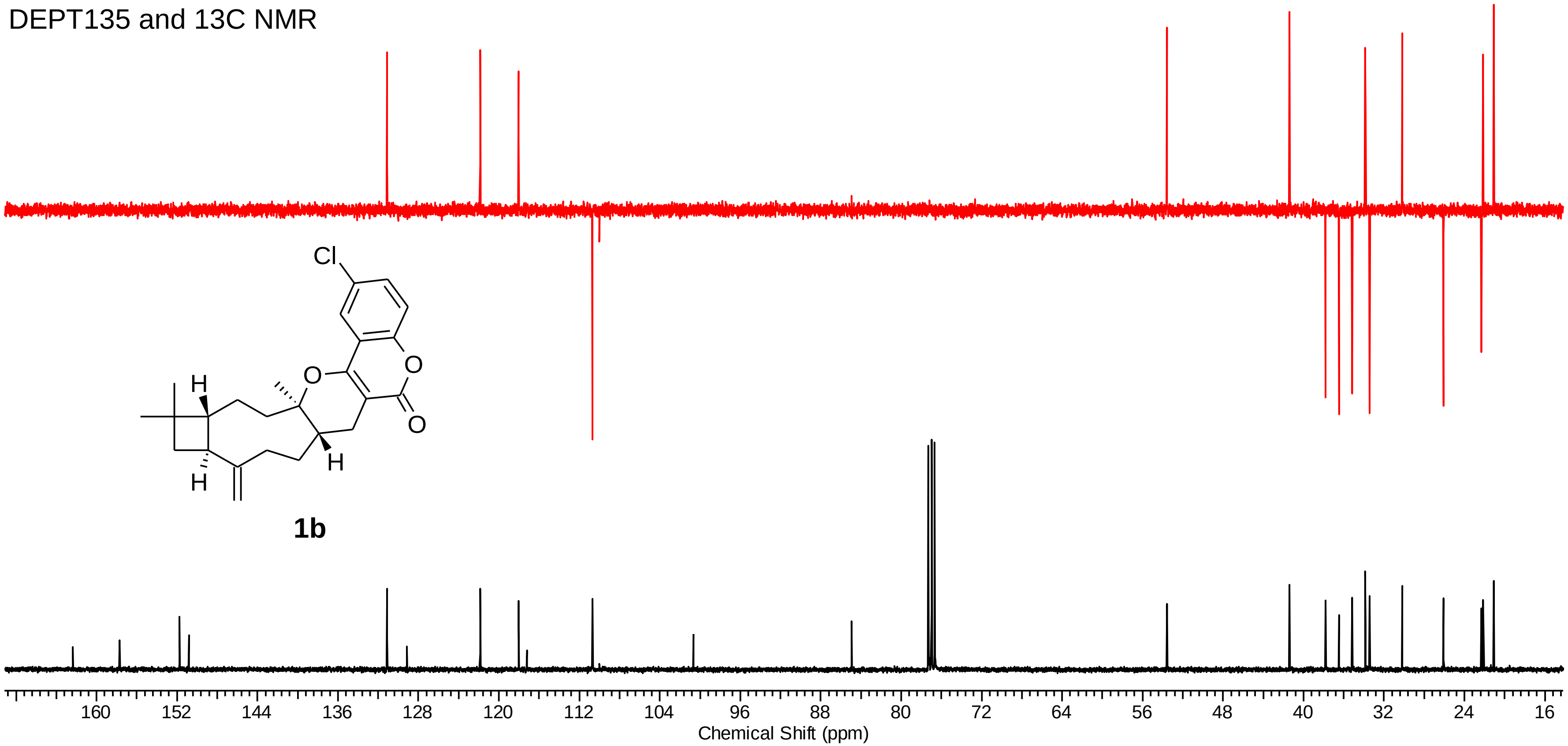

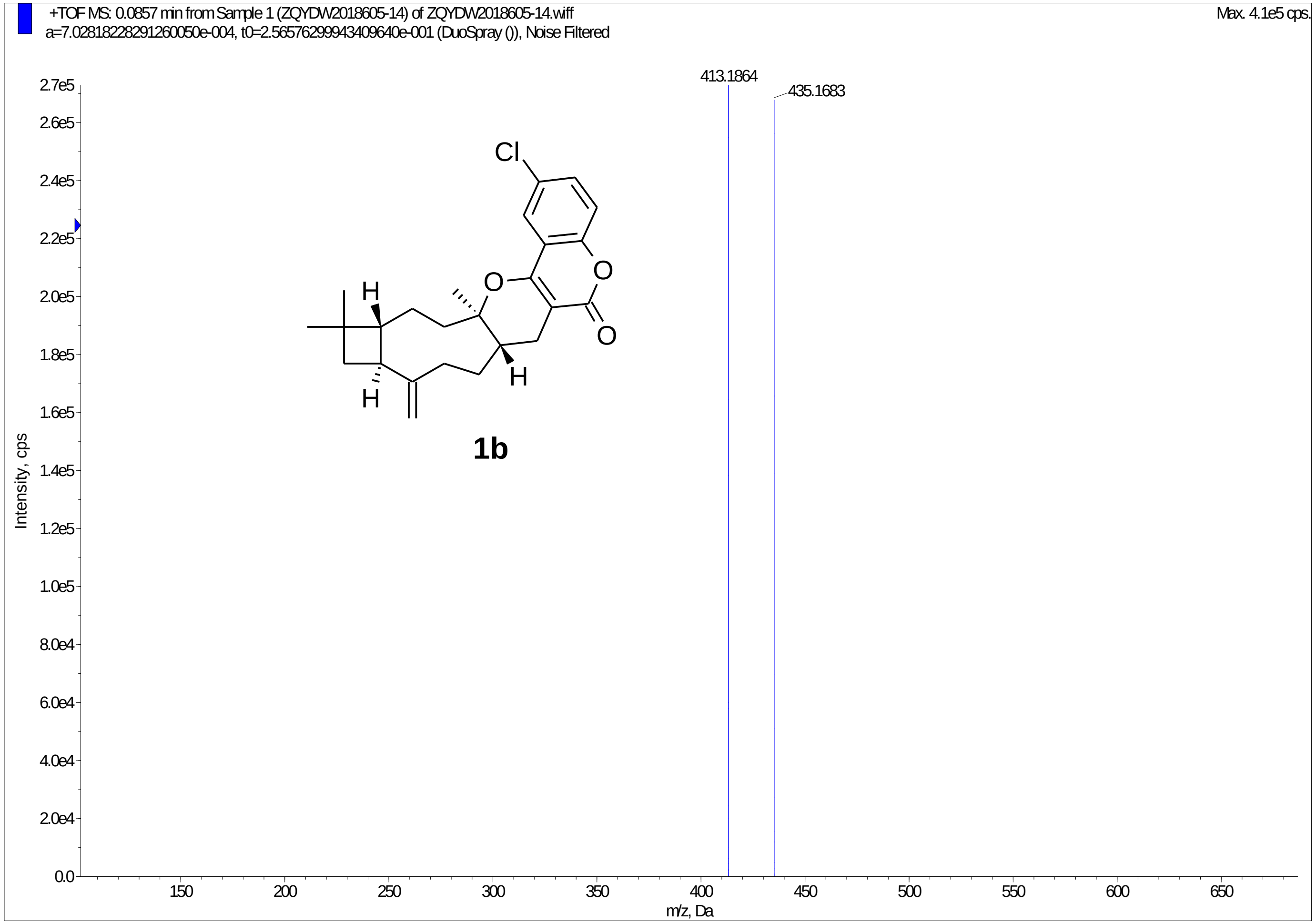
+TOF MS: 0.0857 min from Sample 1 (ZQYDW2018605-14) of ZQYDW2018605-14.wiff
a=7.02818228291260050e-004, t0=2.56576299943409640e-001 (DuoSpray ()), Noise Filtered
Max. 4.1e5 cps.
413.1864
435.1683
Intensity, cps
m/z, Da
1b

Compound 2b

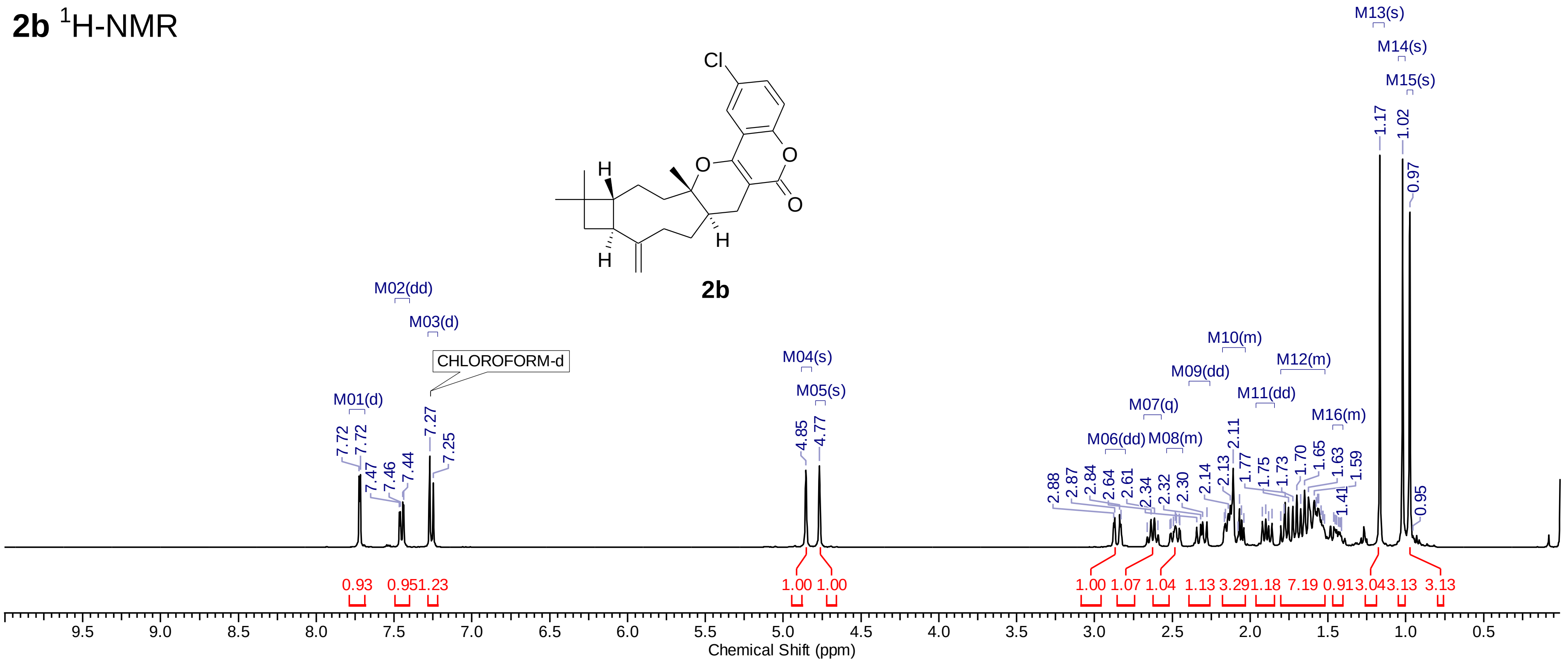

2b ^{1}H-NMR
Cl
O
O
O
H
H
H
2b
M01(d)
M02(dd)
M03(d)
CHLOROFORM-d
M04(s)
M05(s)
M06(dd)
M07(q)
M08(m)
M09(dd)
M10(m)
M11(dd)
M12(m)
M13(s)
M14(s)
M15(s)
M16(m)
7.72
7.72
7.47
7.46
7.44
7.27
7.25
4.85
4.77
2.88
2.87
2.84
2.64
2.61
2.34
2.32
2.30
2.14
2.13
2.11
1.77
1.75
1.73
1.70
1.65
1.63
1.59
1.41
1.17
1.02
0.97
0.95
0.93
0.95
1.23
1.00
1.00
1.00
1.07
1.04
1.13
3.29
1.18
7.19
0.91
3.04
3.13
3.13
9.5
9.0
8.5
8.0
7.5
7.0
6.5
6.0
5.5
5.0
4.5
4.0
3.5
3.0
2.5
2.0
1.5
1.0
0.5
Chemical Shift (ppm)

DEPT135 and ^{13}C NMR

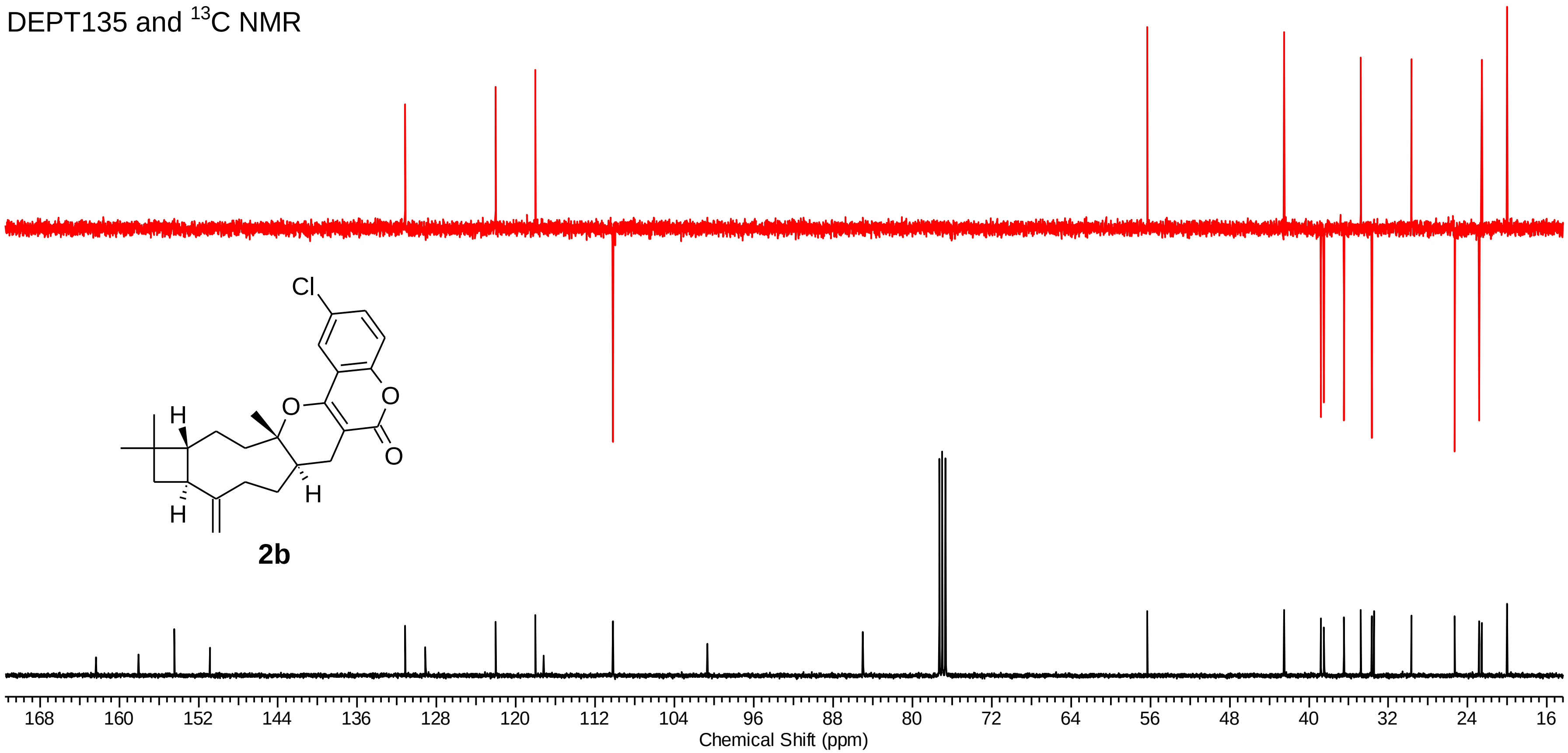

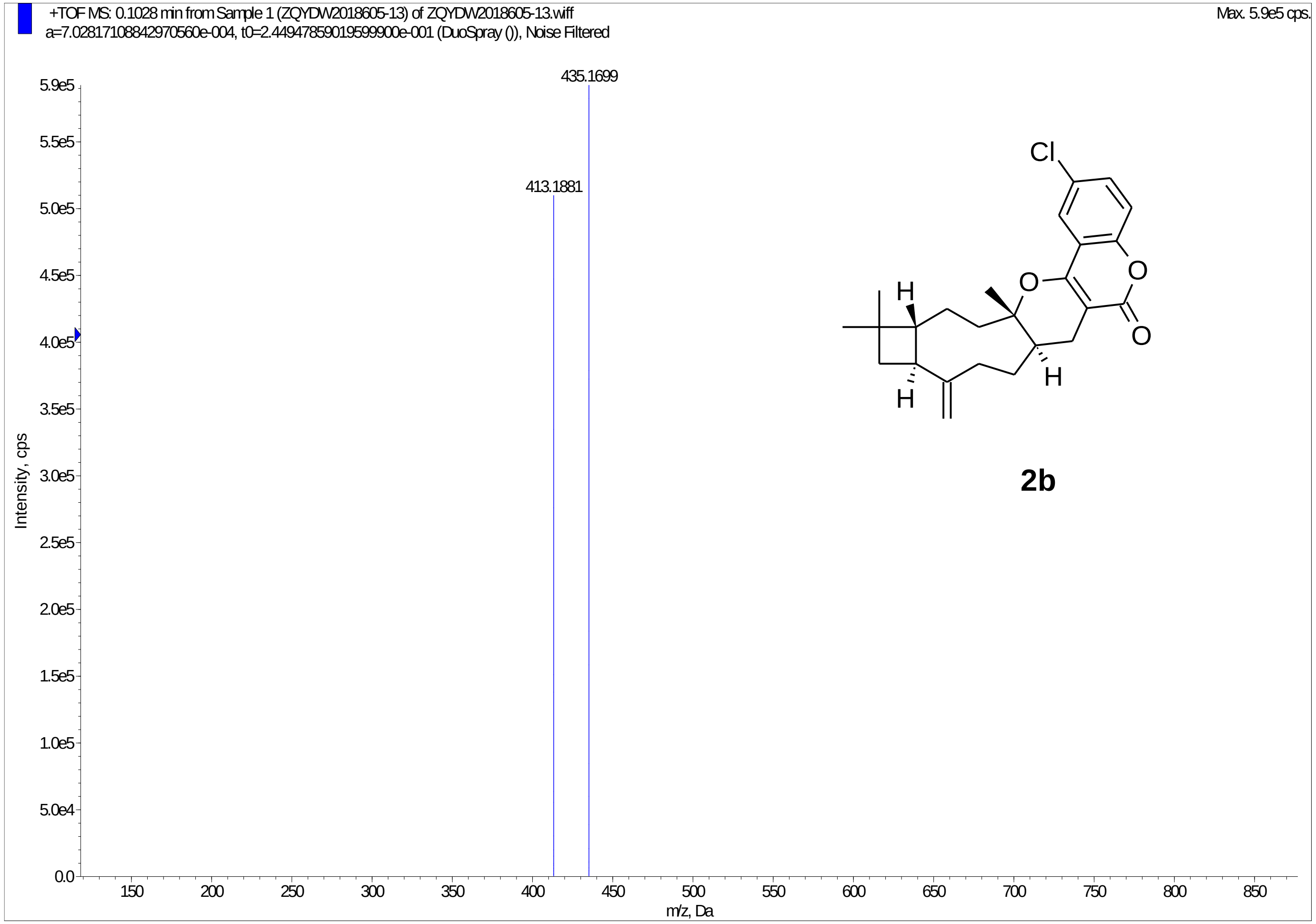
+TOF MS: 0.1028 min from Sample 1 (ZQYDW2018605-13) of ZQYDW2018605-13.wiff
a=7.02817108842970560e-004, t0=2.44947859019599900e-001 (DuoSpray ()), Noise Filtered
Max. 5.9e5 cps.
435.1699
413.1881
Intensity, cps
m/z, Da
Cl
O
O
O
H
H
H
2b

Compound 3b

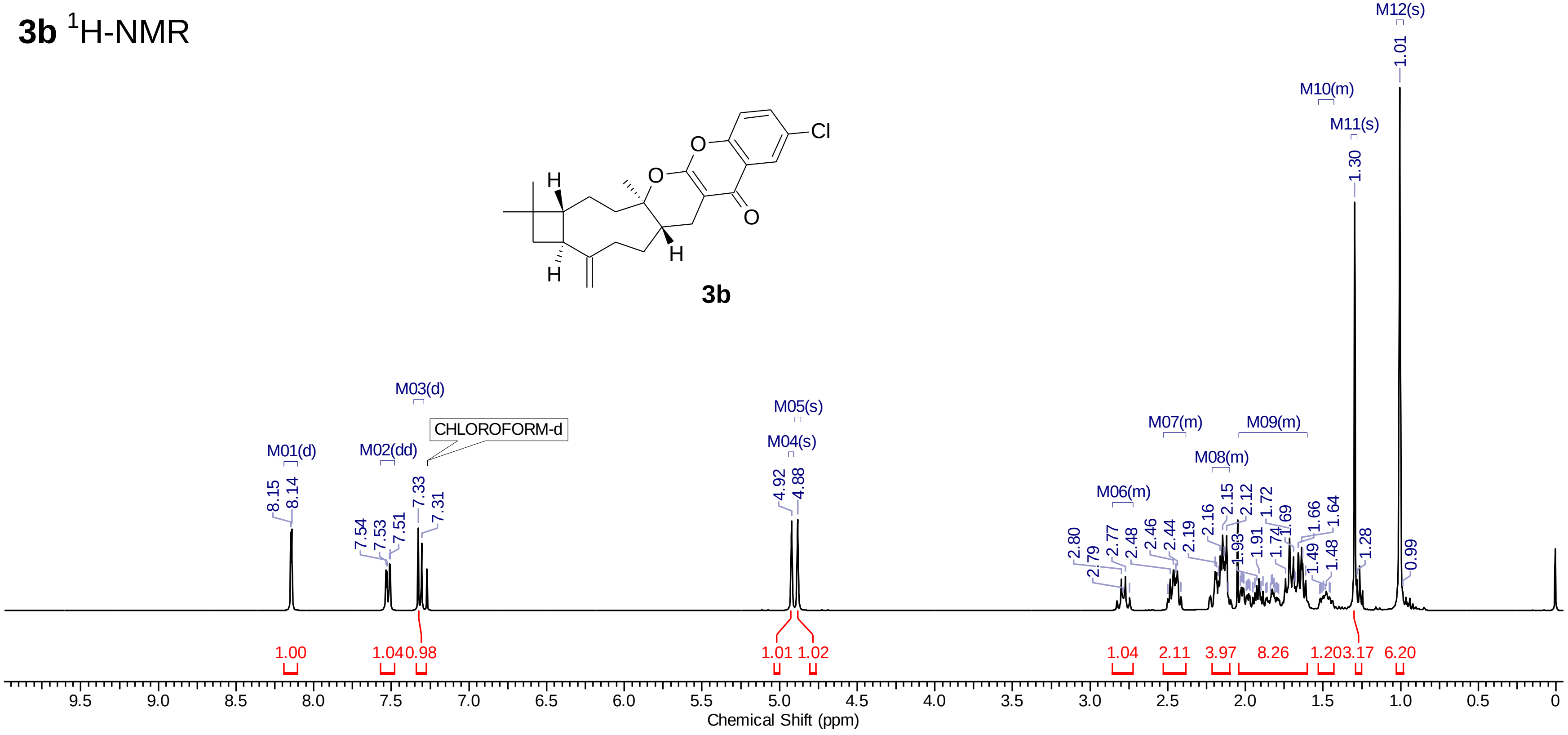

3b ^{1}H-NMR
H
O
O
Cl
O
H
H
3b
M01(d)
M02(dd)
M03(d)
CHLOROFORM-d
M04(s)
M05(s)
M06(m)
M07(m)
M08(m)
M09(m)
M10(m)
M11(s)
M12(s)
Chemical Shift (ppm)

DEPT135 and ^{13}C NMR

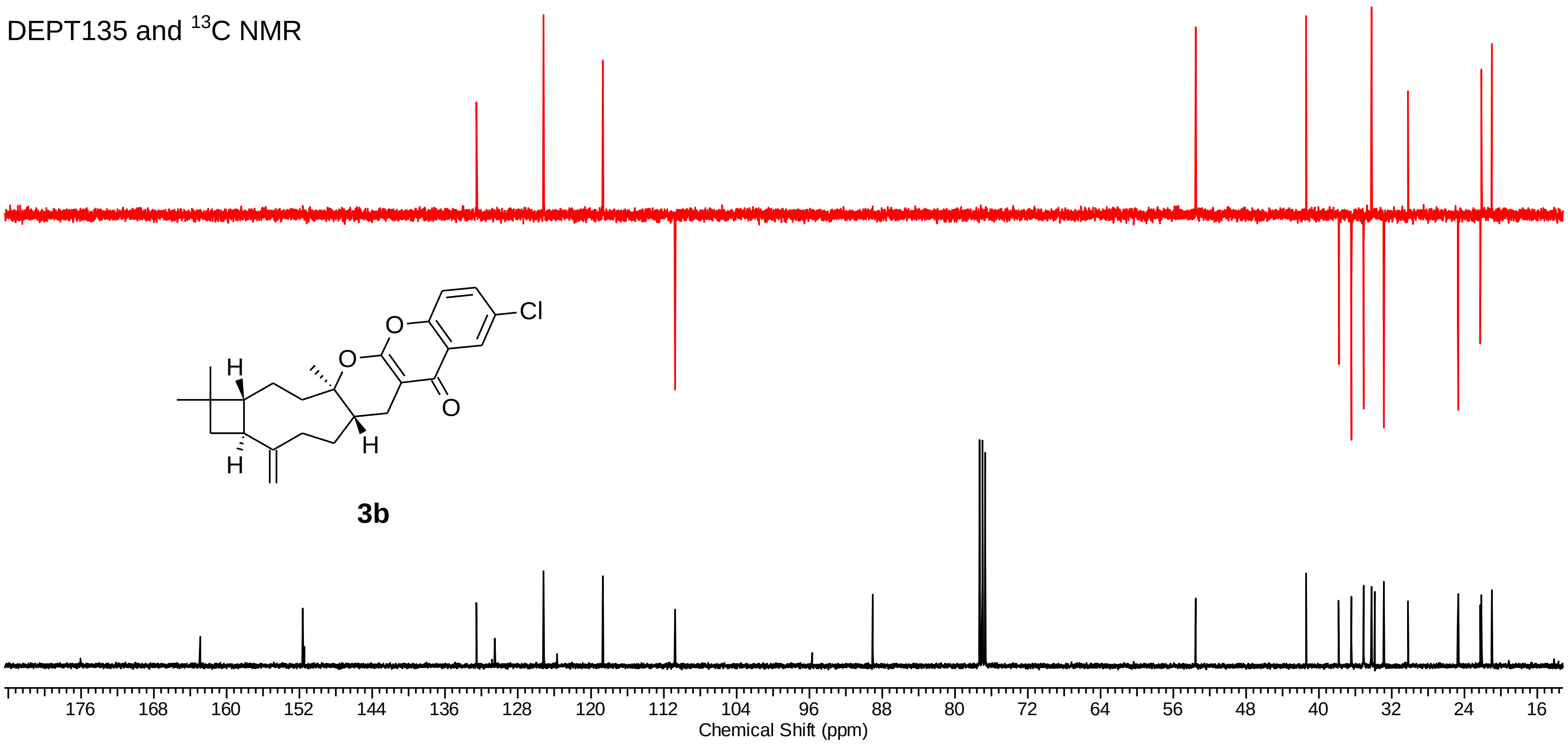

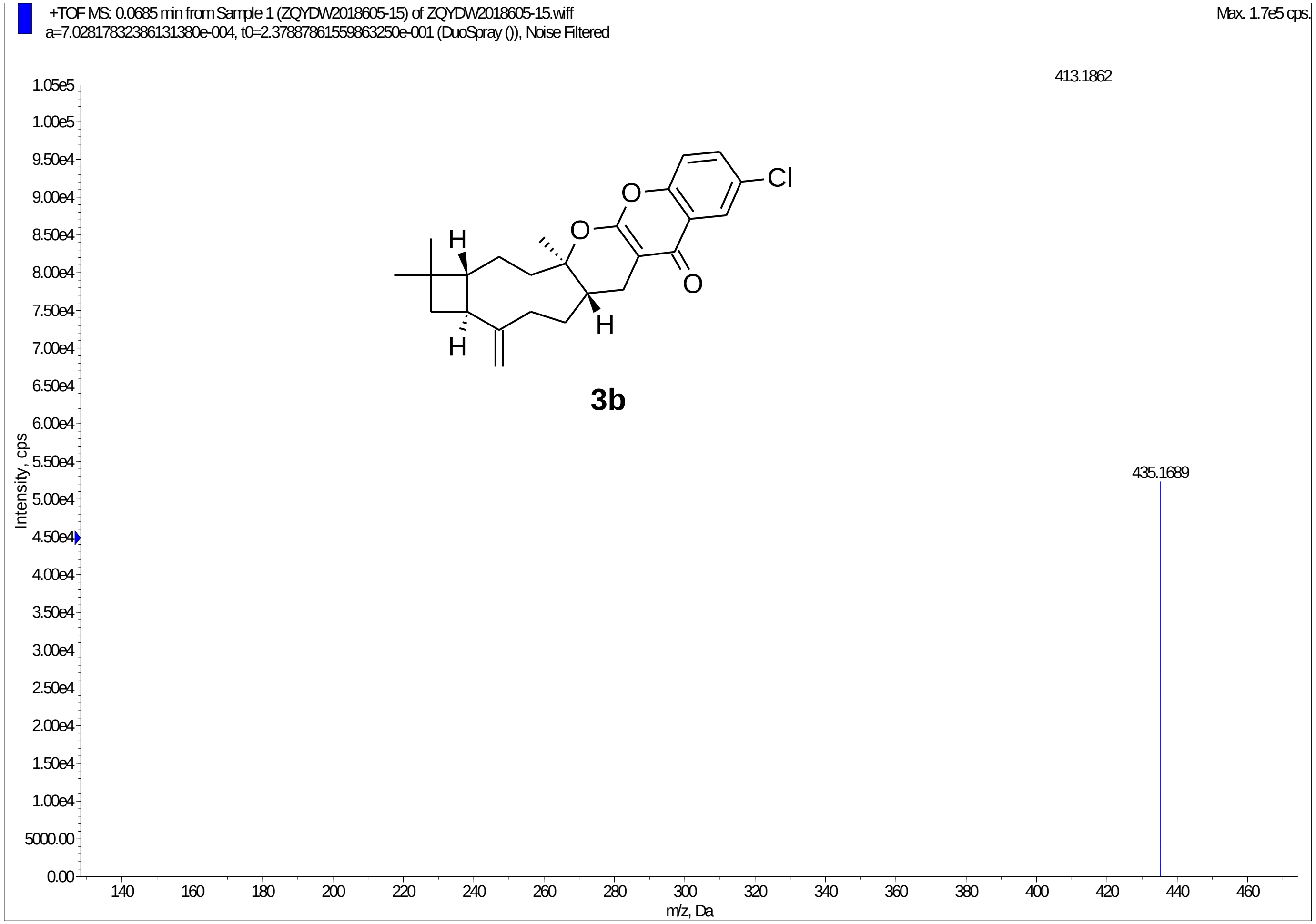
+TOF MS: 0.0685 min from Sample 1 (ZQYDW2018605-15) of ZQYDW2018605-15.wiff
a=7.02817832386131380e-004, t0=2.37887861559863250e-001 (DuoSpray ()), Noise Filtered
Max. 1.7e5 cps.
413.1862
435.1689
3b
Intensity, cps
m/z, Da

Compound 4b

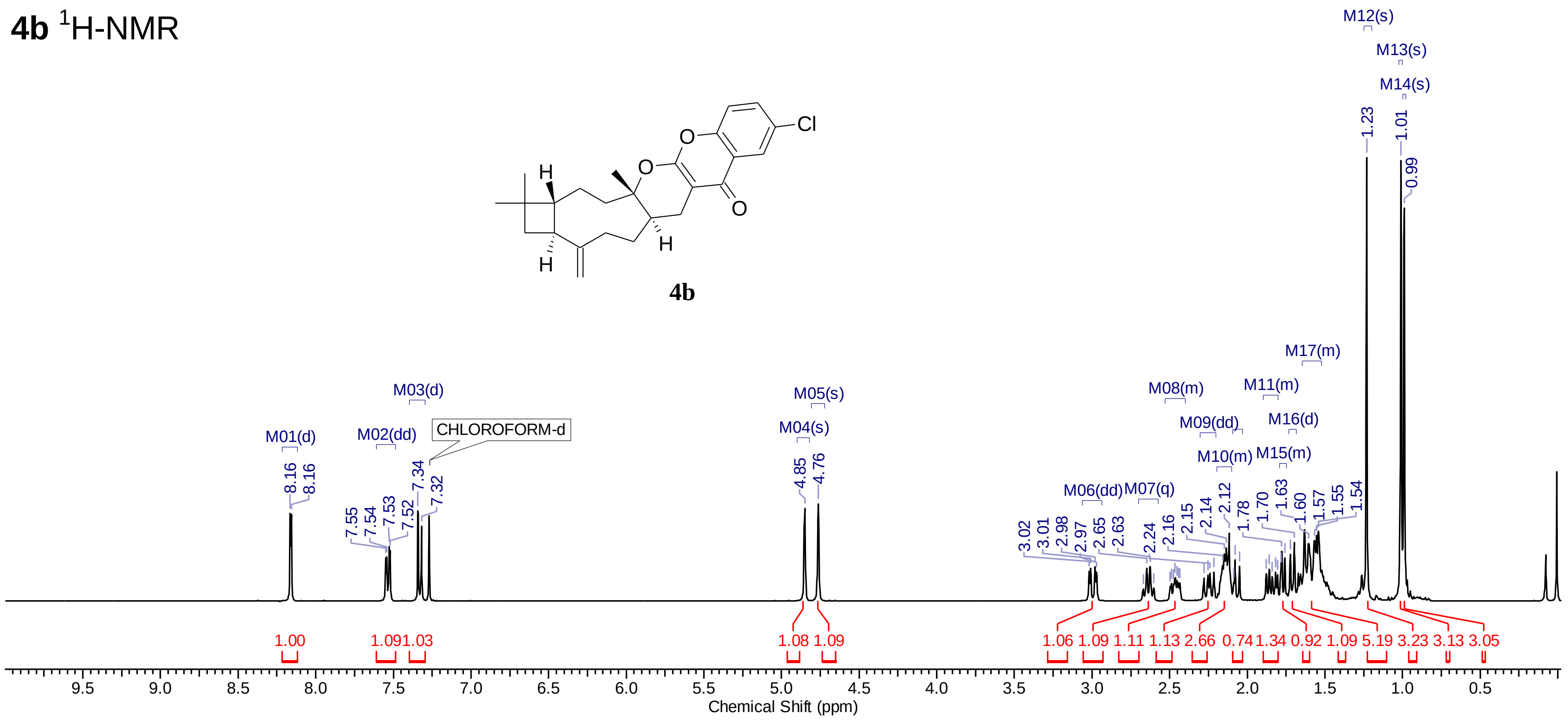
4b 1H-NMR
Cl
O
O
O
H
H
H
4b
M01(d)
M02(dd)
M03(d)
CHLOROFORM-d
M04(s)
M05(s)
M06(dd)
M07(q)
M08(m)
M09(dd)
M10(m)
M11(m)
M12(s)
M13(s)
M14(s)
M15(m)
M16(d)
M17(m)
8.16 8.16
7.55 7.54 7.53 7.52
7.34 7.32
4.85 4.76
3.02 3.01 2.98 2.97 2.65 2.63 2.24 2.16 2.15 2.14 2.12 1.78 1.70 1.63 1.60 1.57 1.55 1.54
1.23 1.01 0.99
1.00 1.09 1.03 1.08 1.09 1.06 1.09 1.11 1.13 2.66 0.74 1.34 0.92 1.09 5.19 3.23 3.13 3.05
9.5 9.0 8.5 8.0 7.5 7.0 6.5 6.0 5.5 5.0 4.5 4.0 3.5 3.0 2.5 2.0 1.5 1.0 0.5
Chemical Shift (ppm)

DEPT135 and ^{13}C NMR

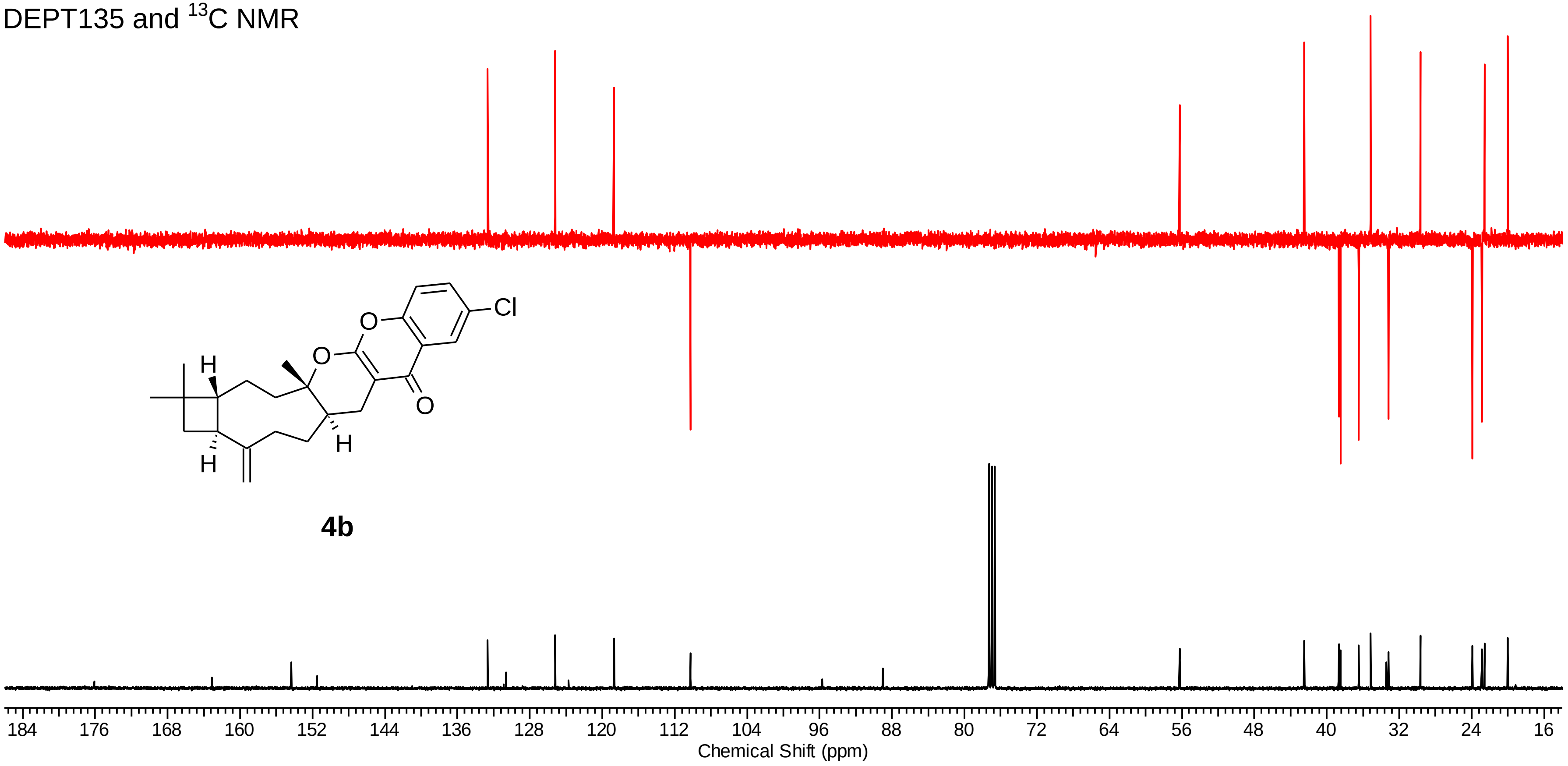

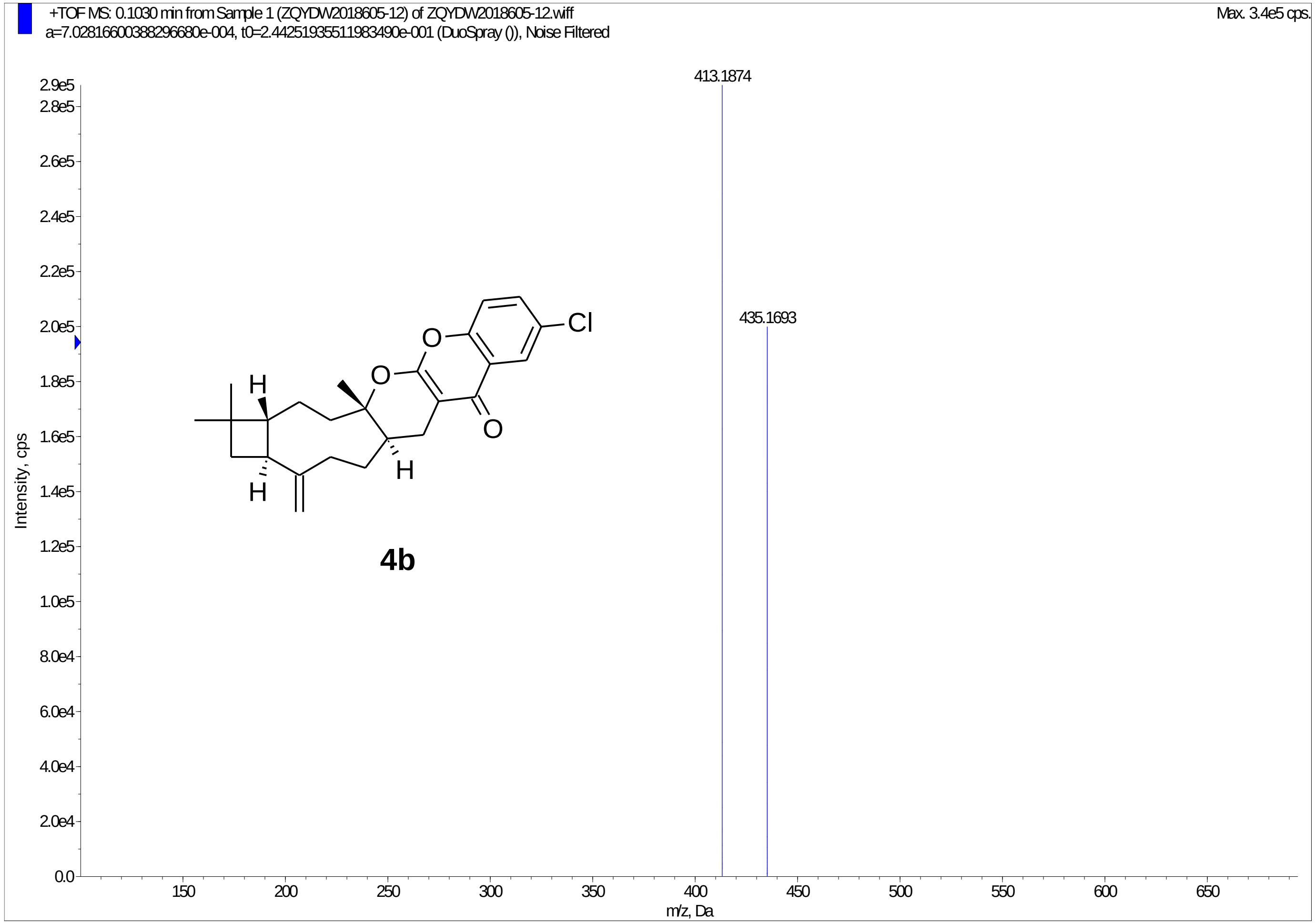
+TOF MS: 0.1030 min from Sample 1 (ZQYDW2018605-12) of ZQYDW2018605-12.wiff
a=7.02816600388296680e-004, t0=2.44251935511983490e-001 (DuoSpray ()), Noise Filtered
Max. 3.4e5 cps.
413.1874
435.1693
Intensity, cps
m/z, Da
Cl
4b

Compound 5b

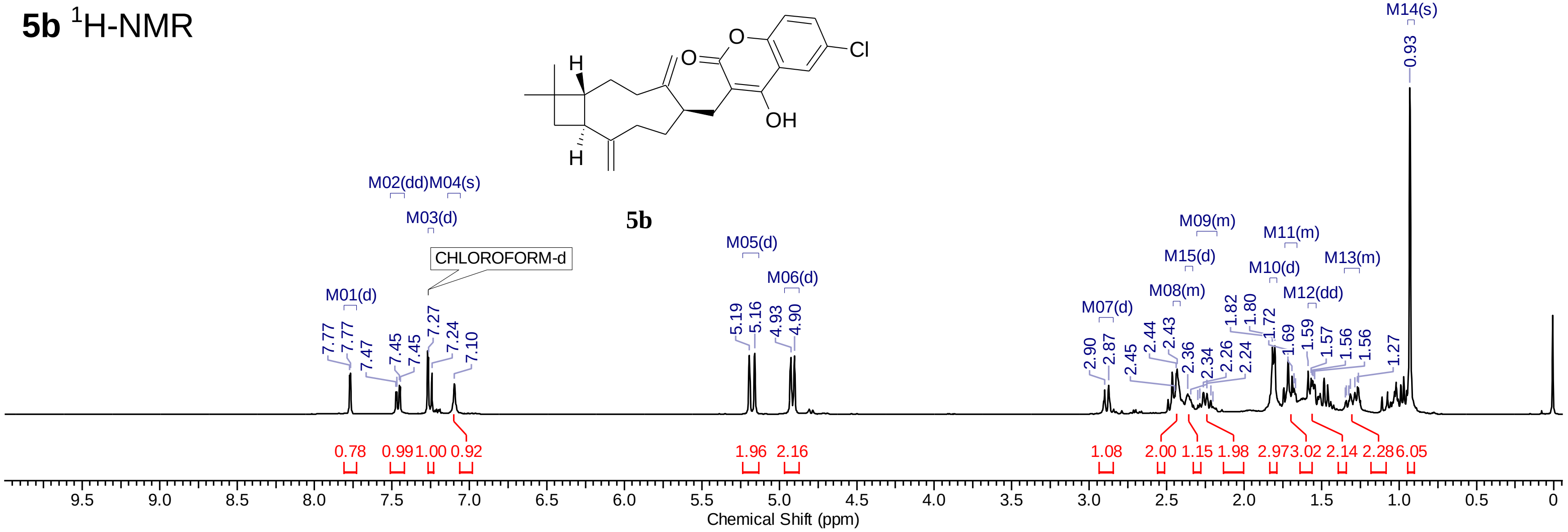
5b ¹H-NMR
O
O
Cl
OH
H
H
5b
CHLOROFORM-d
M01(d)
M02(dd)
M03(d)
M04(s)
M05(d)
M06(d)
M07(d)
M08(m)
M09(m)
M10(d)
M11(m)
M12(dd)
M13(m)
M14(s)
M15(d)
7.77
7.77
7.47
7.45
7.45
7.27
7.24
7.10
5.19
5.16
4.93
4.90
2.90
2.87
2.45
2.44
2.43
2.36
2.34
2.26
2.24
1.82
1.80
1.72
1.69
1.59
1.57
1.56
1.56
1.27
0.93
0.78
0.99
1.00
0.92
1.96
2.16
1.08
2.00
1.15
1.98
2.97
3.02
2.14
2.28
6.05
9.5
9.0
8.5
8.0
7.5
7.0
6.5
6.0
5.5
5.0
4.5
4.0
3.5
3.0
2.5
2.0
1.5
1.0
0.5
0
Chemical Shift (ppm)

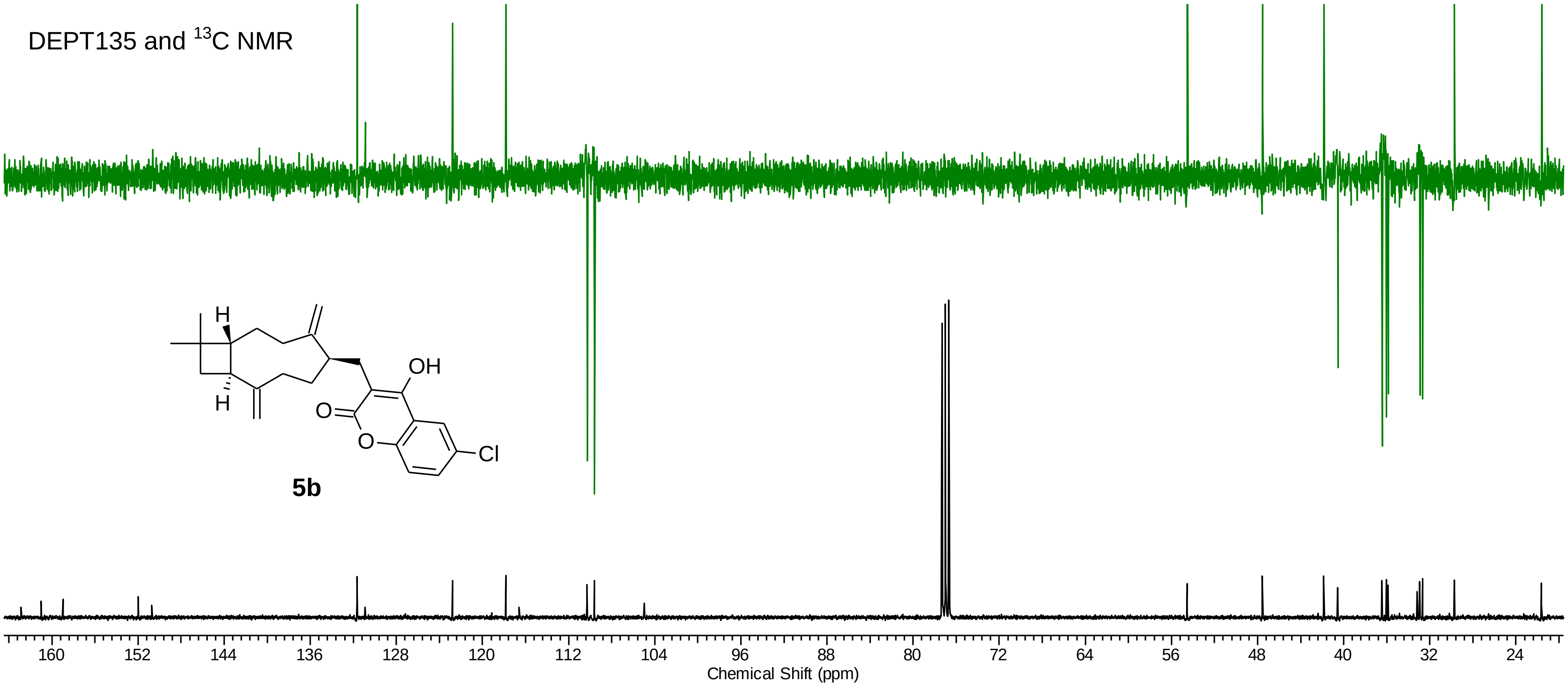

DEPT135 and 13C NMR
H
H
OH
O
O
Cl
5b
160
152
144
136
128
120
112
104
96
88
80
72
64
56
48
40
32
24
Chemical Shift (ppm)

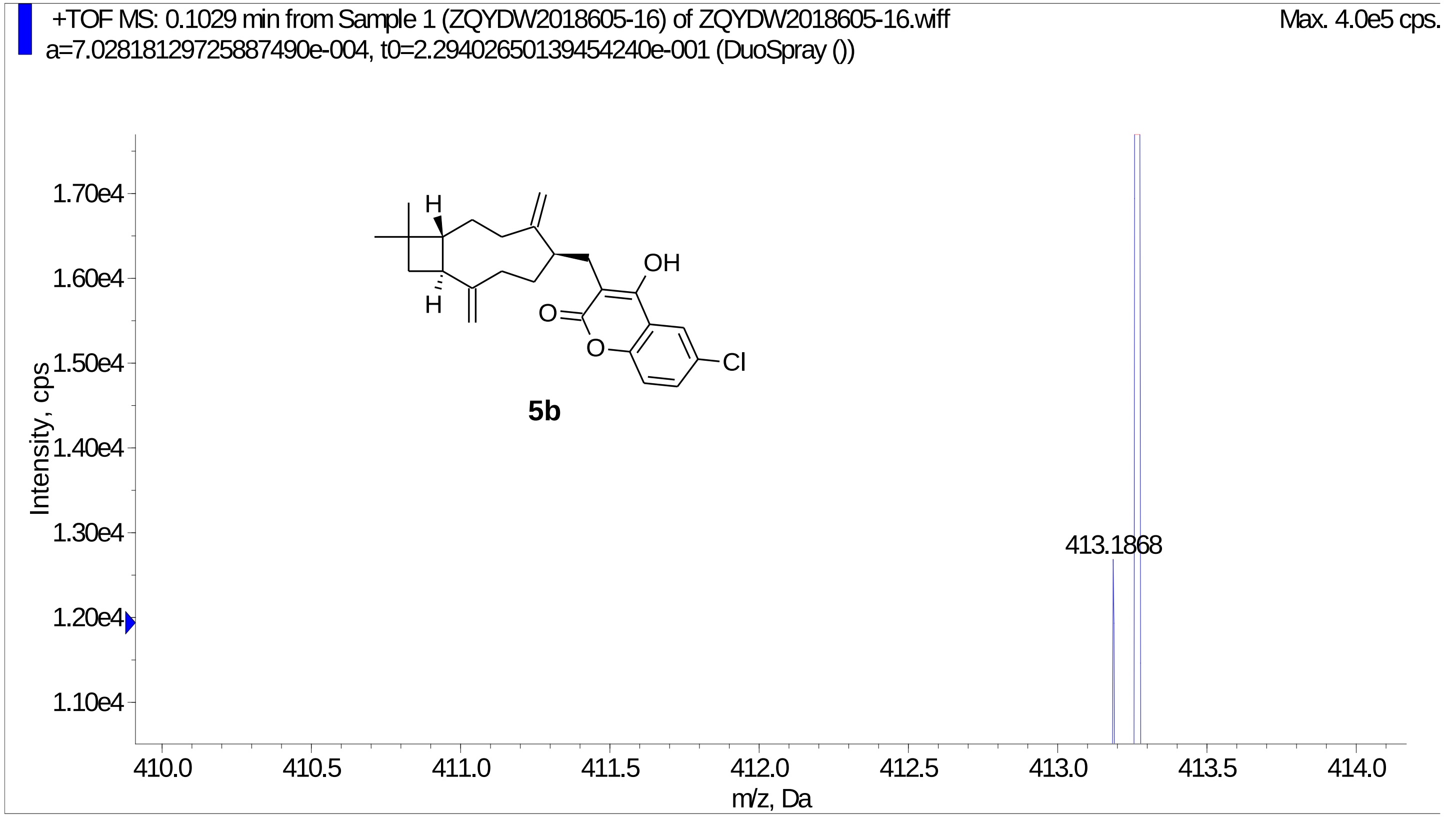
+TOF MS: 0.1029 min from Sample 1 (ZQYDW2018605-16) of ZQYDW2018605-16.wiff
Max. 4.0e5 cps.
a=7.02818129725887490e-004, t0=2.29402650139454240e-001 (DuoSpray ())
Intensity, cps
1.70e4
1.60e4
1.50e4
1.40e4
1.30e4
1.20e4
1.10e4
H
H
OH
O
O
Cl
5b
413.1868
410.0
410.5
411.0
411.5
412.0
412.5
413.0
413.5
414.0
m/z, Da

Compound 1c

1c ^{1}H-NMR

YDW84-3 H.ESP

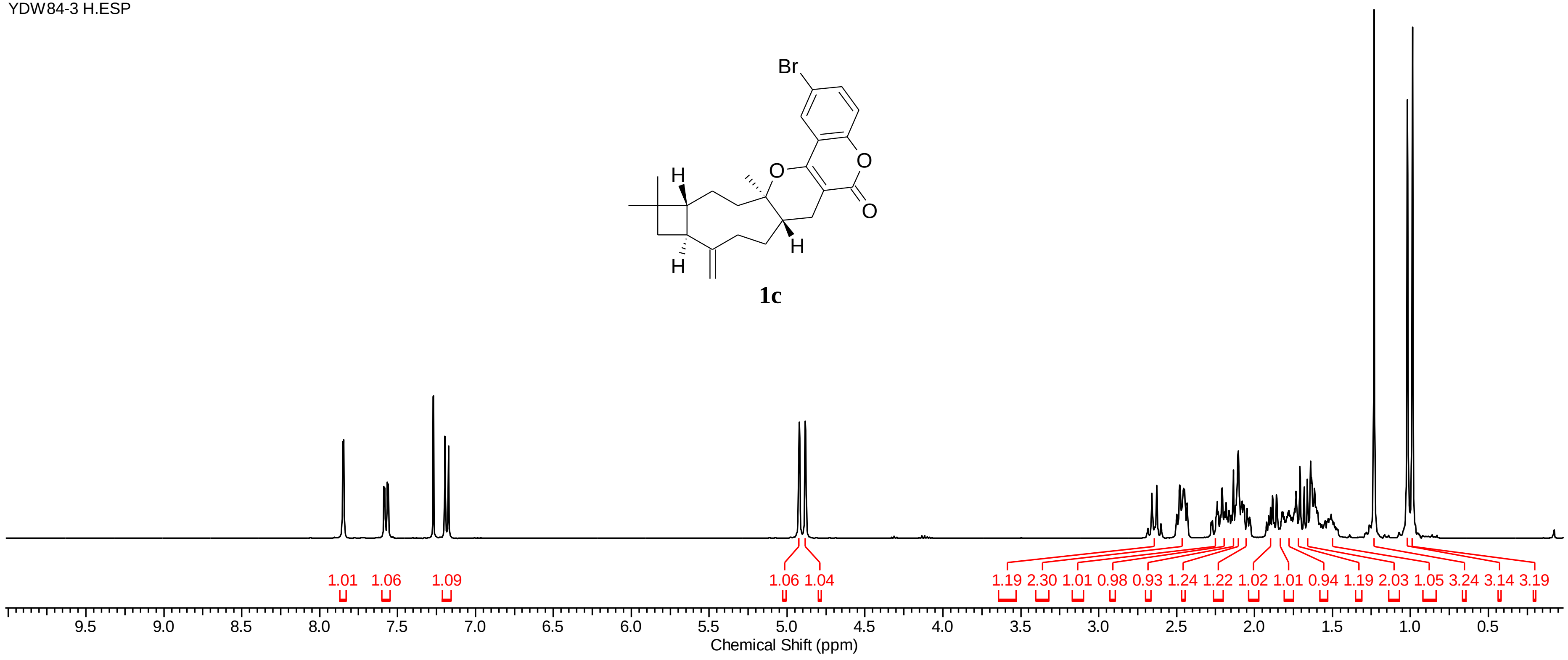

DEPT135 and ^{13}C NMR

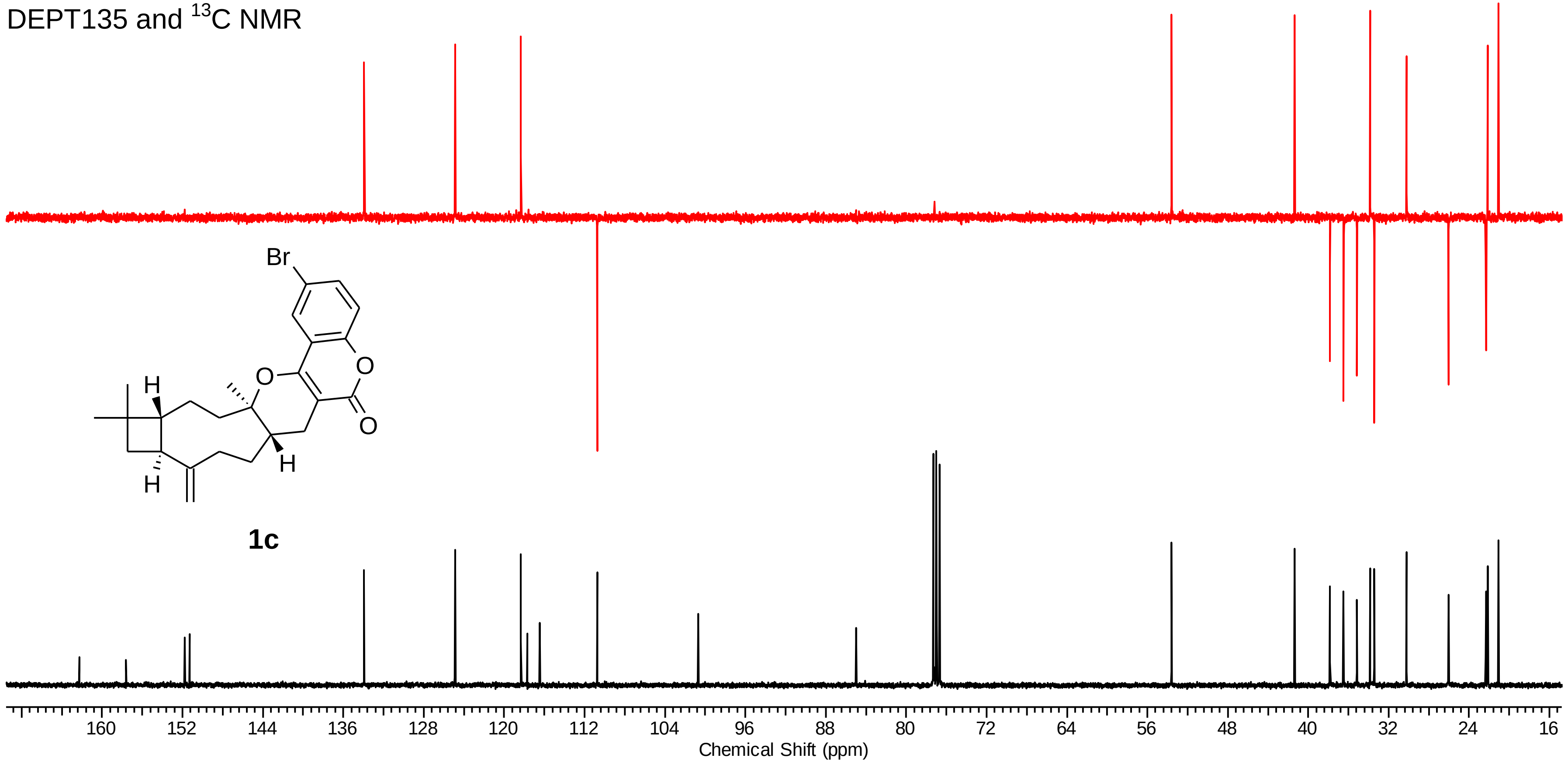

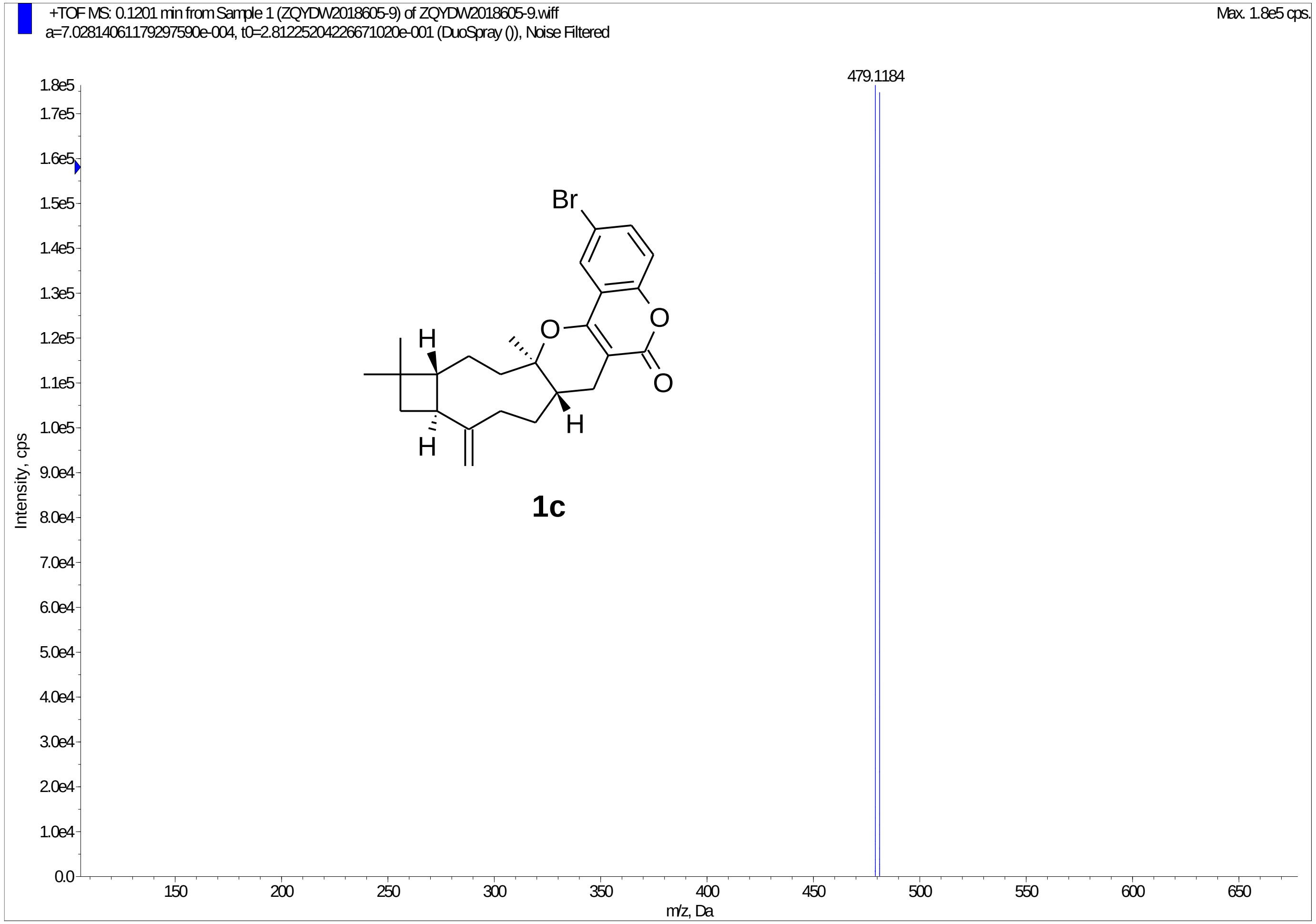
+TOF MS: 0.1201 min from Sample 1 (ZQYDW2018605-9) of ZQYDW2018605-9.wiff
a=7.02814061179297590e-004, t0=2.81225204226671020e-001 (DuoSpray ()), Noise Filtered
Max. 1.8e5 cps.
479.1184
Intensity, cps
m/z, Da
Br
O
O
O
H
H
H
1c

Compound 2c

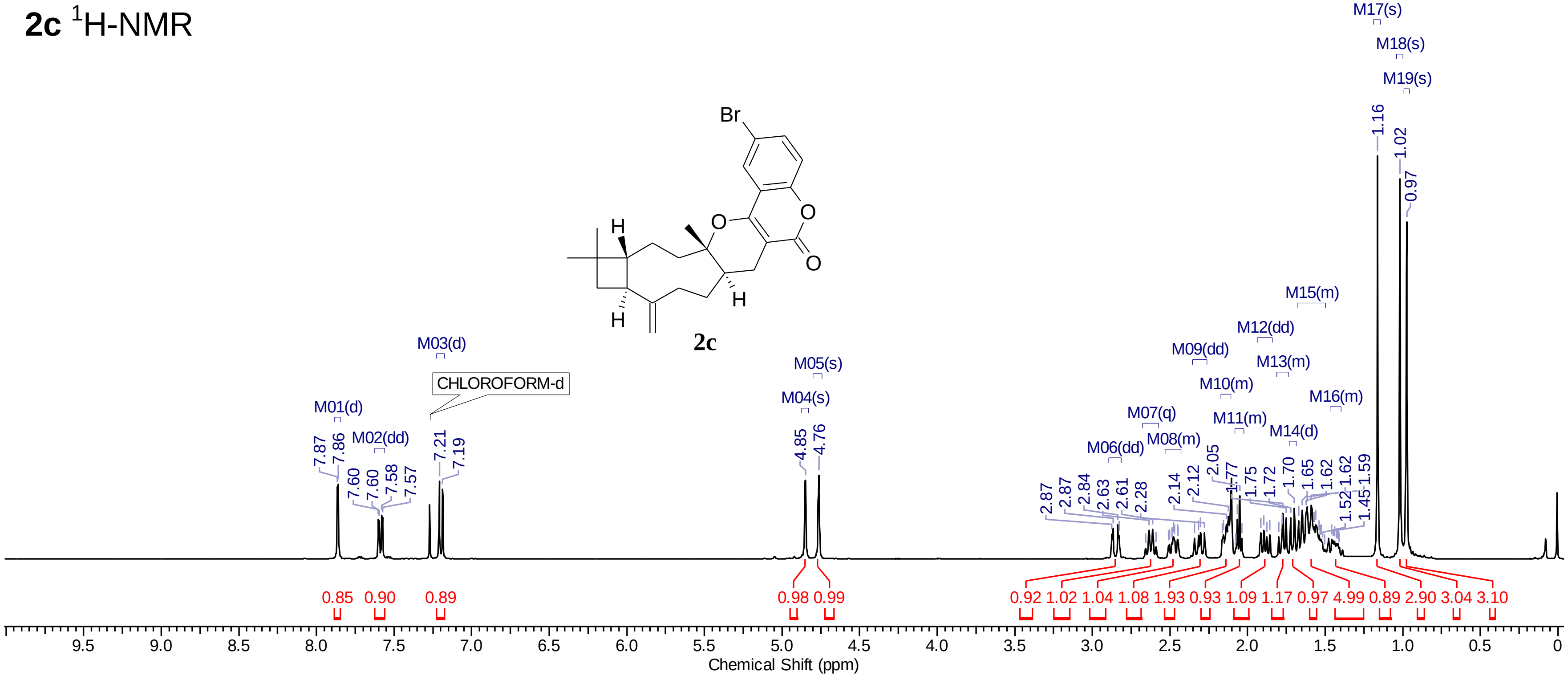
2c ¹H-NMR
Br
O
O
O
H
H
H
2c
CHLOROFORM-d
M01(d)
M02(dd)
M03(d)
M04(s)
M05(s)
M06(dd)
M07(q)
M08(m)
M09(dd)
M10(m)
M11(m)
M12(dd)
M13(m)
M14(d)
M15(m)
M16(m)
M17(s)
M18(s)
M19(s)
0.85 0.90 0.89 0.98 0.99 0.92 1.02 1.04 1.08 1.93 0.93 1.09 1.17 0.97 4.99 0.89 2.90 3.04 3.10
Chemical Shift (ppm)

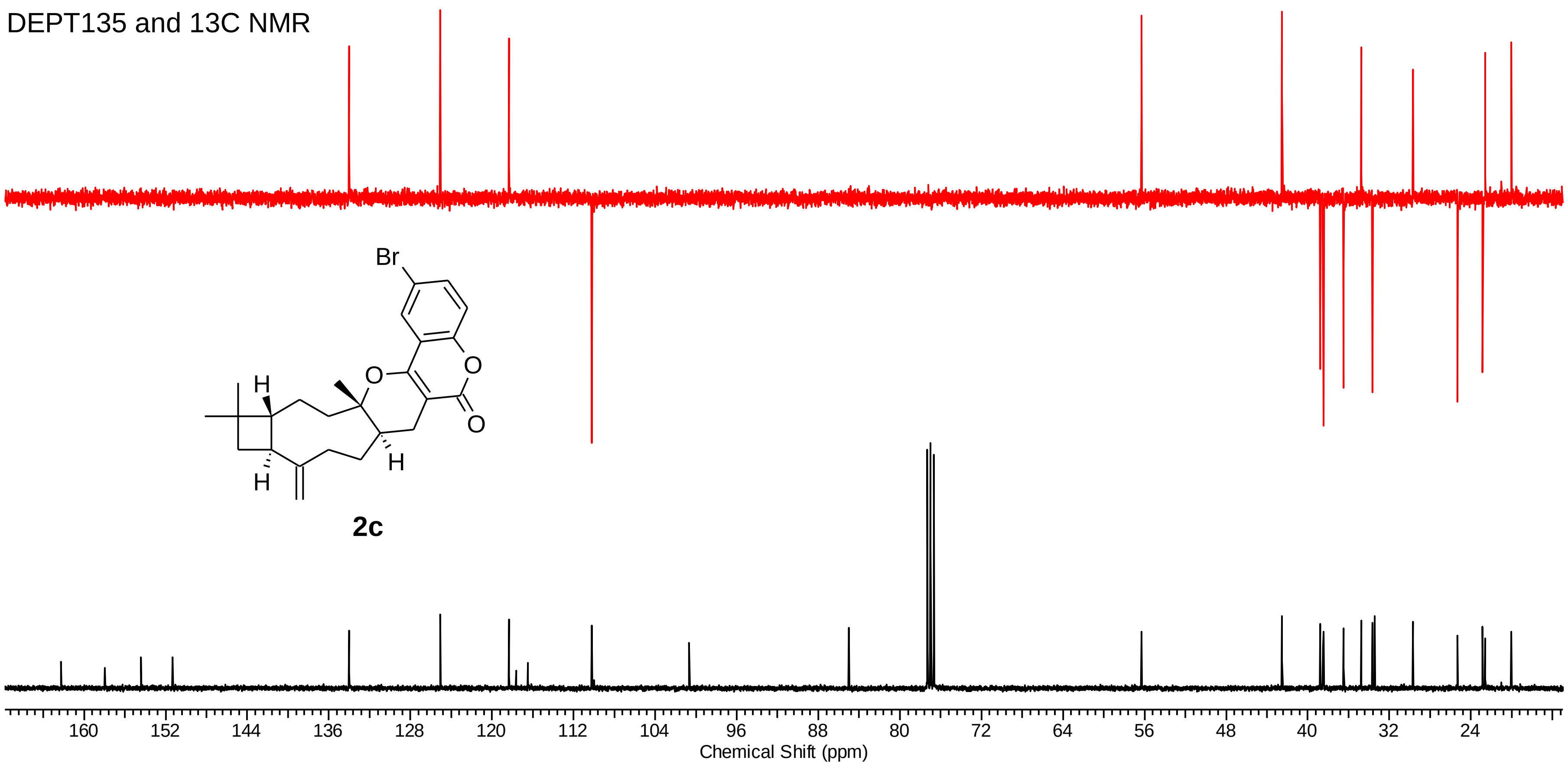
DEPT135 and 13C NMR
Br
O
O
O
H
H
H
2c
160
152
144
136
128
120
112
104
96
88
80
72
64
56
48
40
32
24
Chemical Shift (ppm)

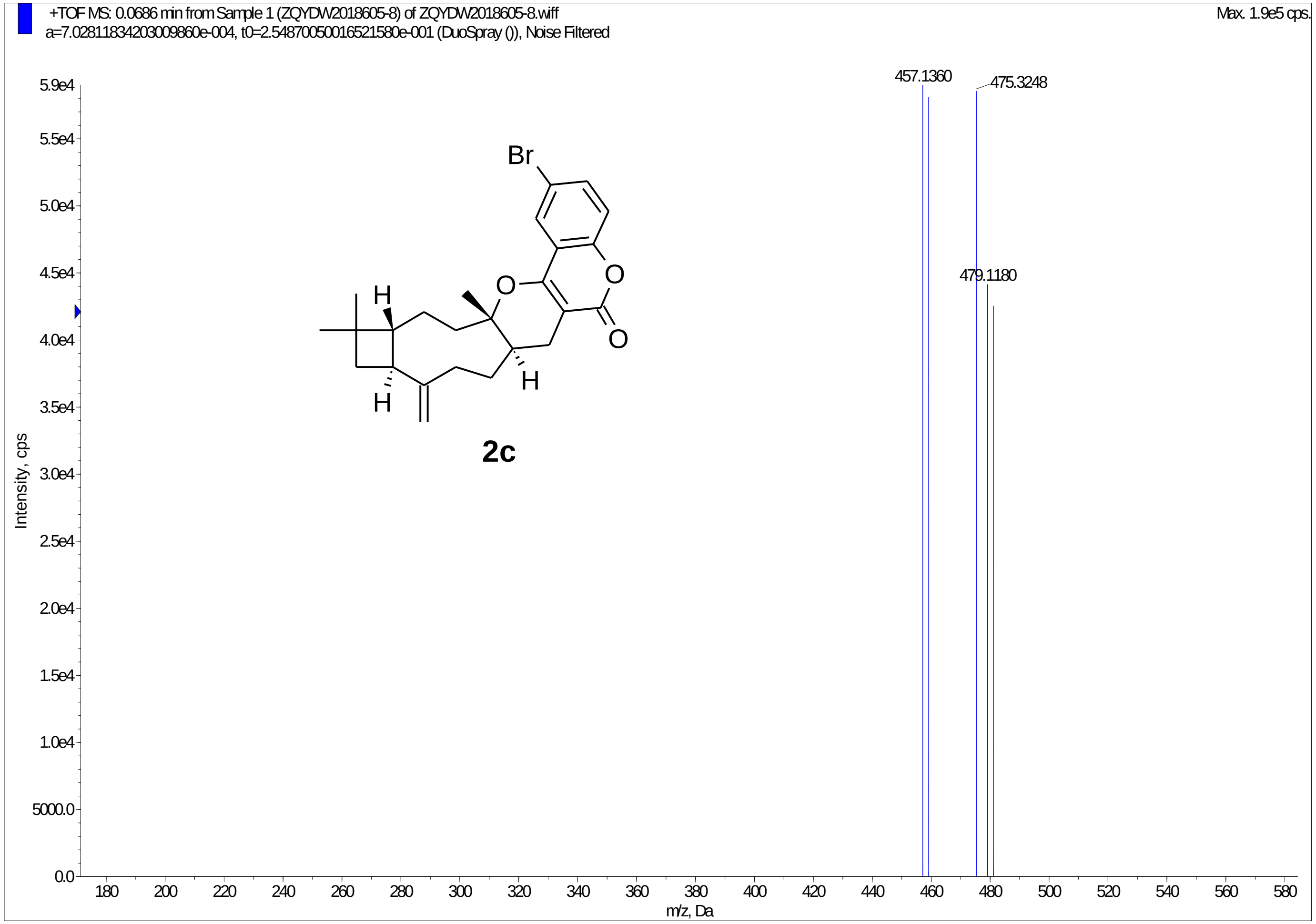
+TOF MS: 0.0686 min from Sample 1 (ZQYDW2018605-8) of ZQYDW2018605-8.wiff
a=7.02811834203009860e-004, t0=2.54870050016521580e-001 (DuoSpray ()), Noise Filtered
Max. 1.9e5 cps.
457.1360
475.3248
479.1180
Br
O
O
O
H
H
H
2c
Intensity, cps
m/z, Da

Compound 3c

3c ^{1}H-NMR

YDW84-5 H.ESP

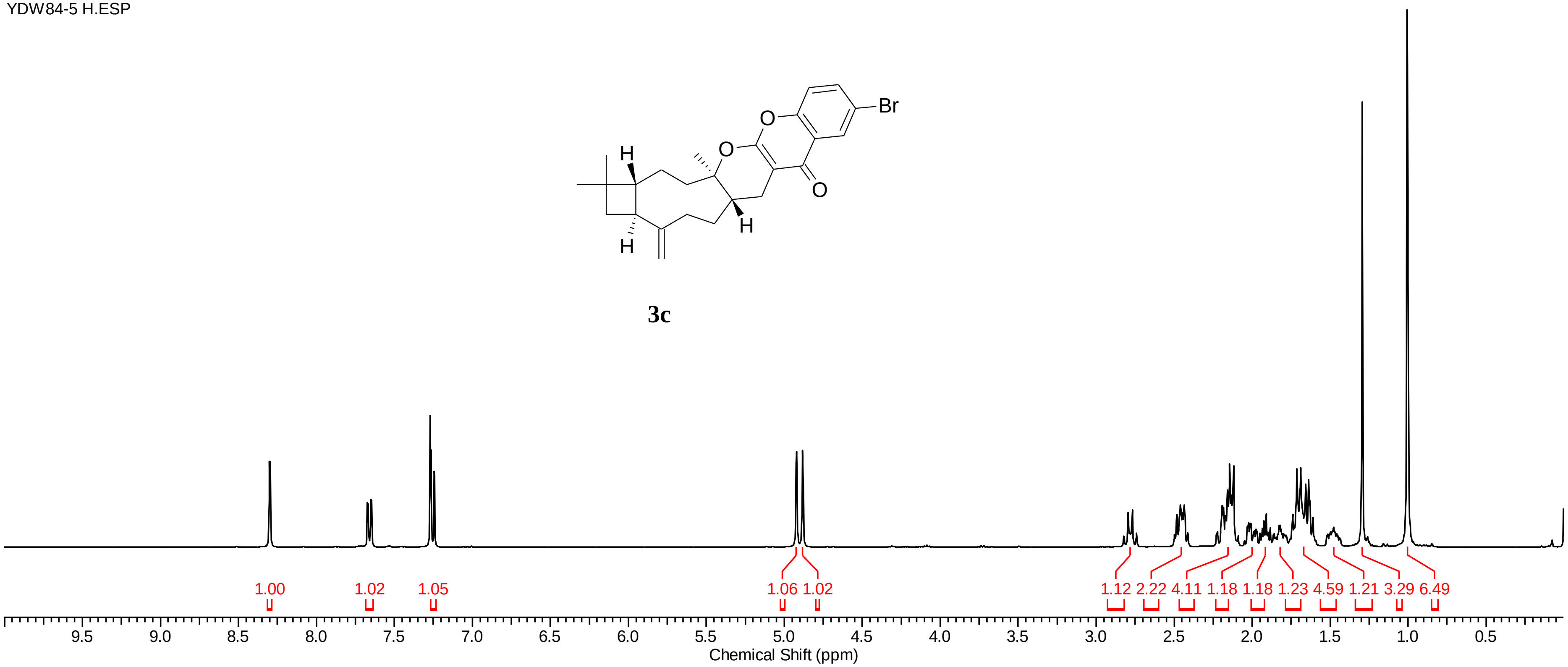

DEPT135 and ^{13}C NMR

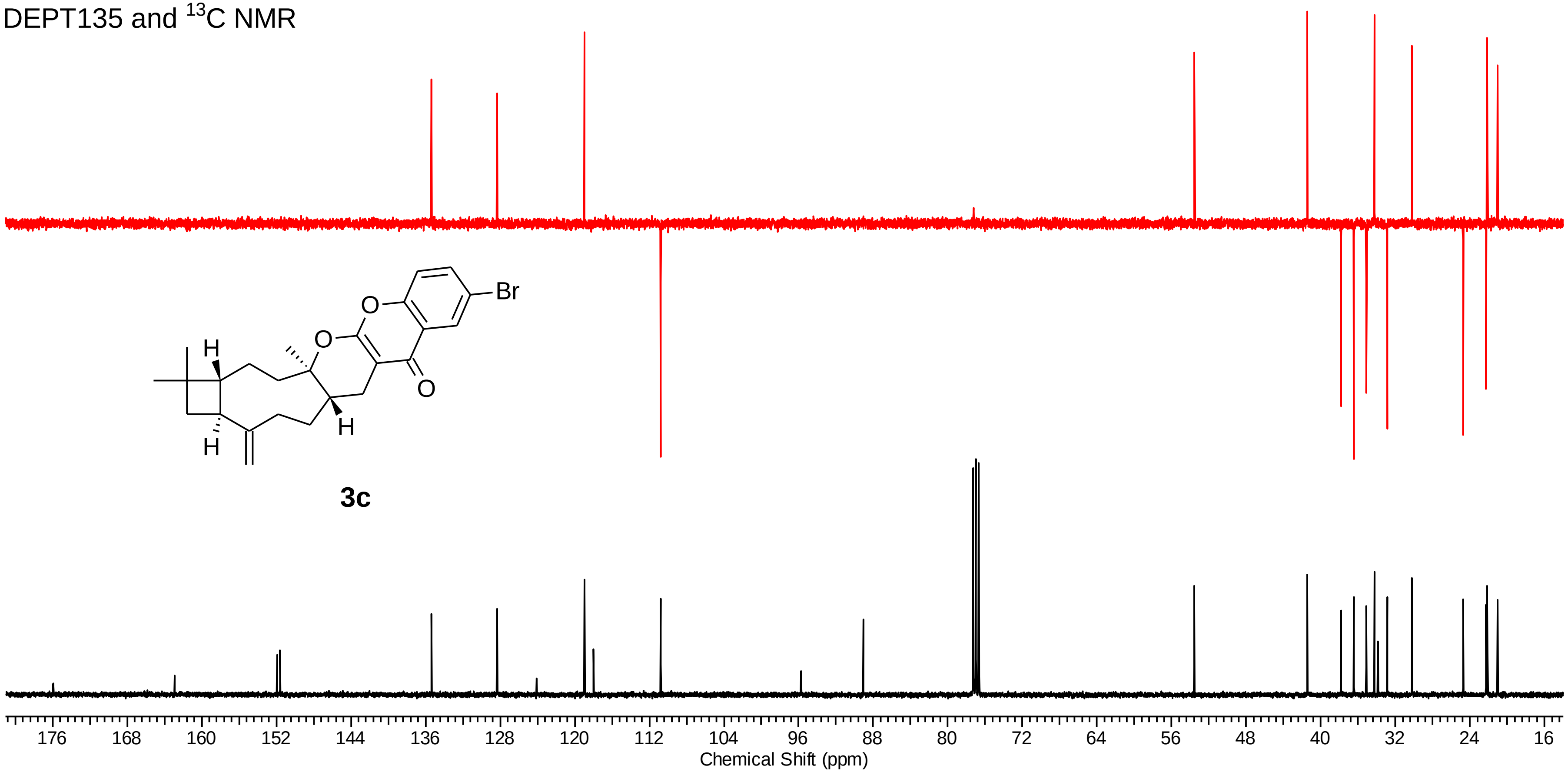

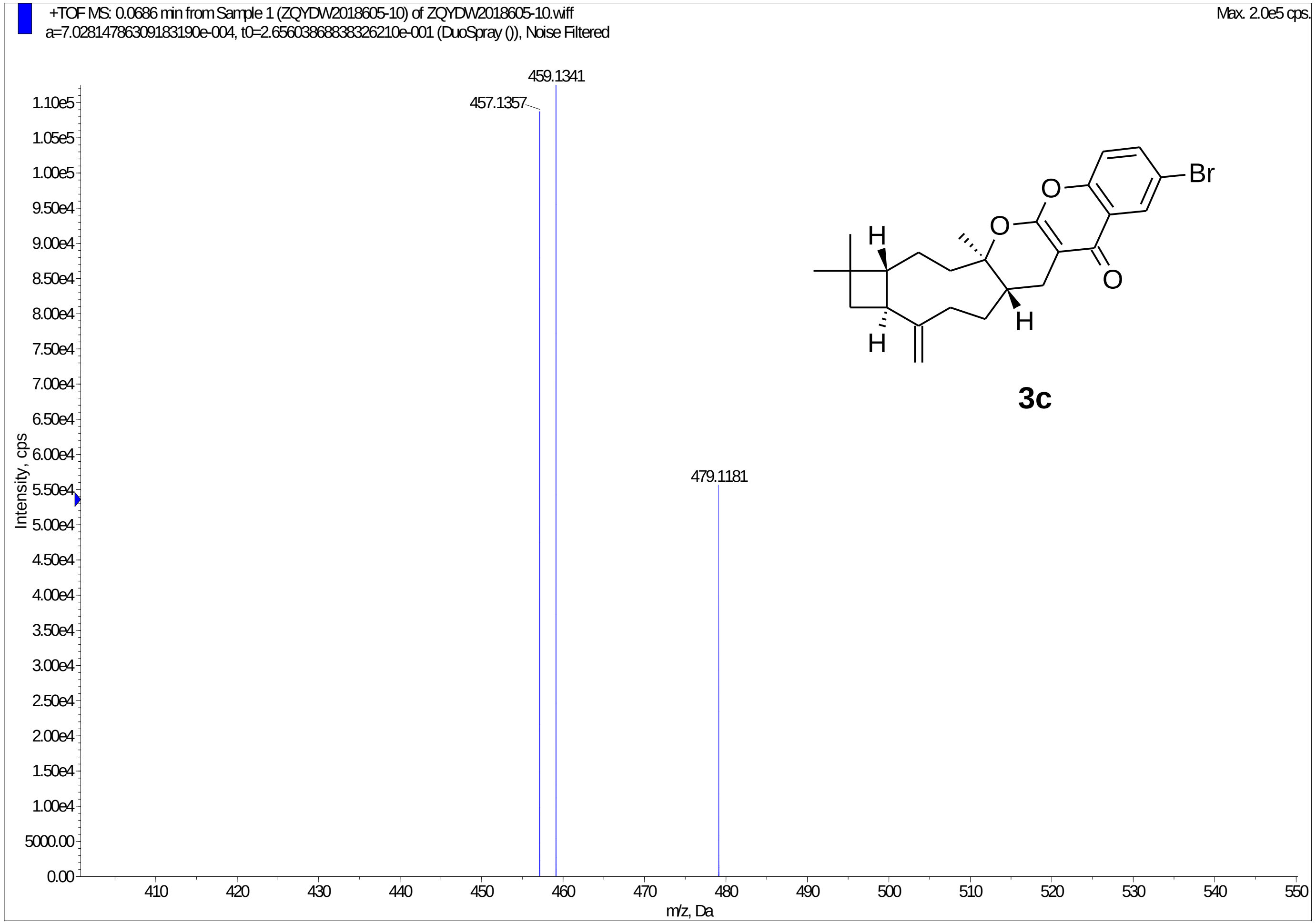
+TOF MS: 0.0686 min from Sample 1 (ZQYDW2018605-10) of ZQYDW2018605-10.wiff
a=7.02814786309183190e-004, t0=2.65603868838326210e-001 (DuoSpray ()), Noise Filtered
Max. 2.0e5 cps.
459.1341
457.1357
479.1181
Intensity, cps
m/z, Da
Br
O
O
O
H
H
H
3c

Compound 4c

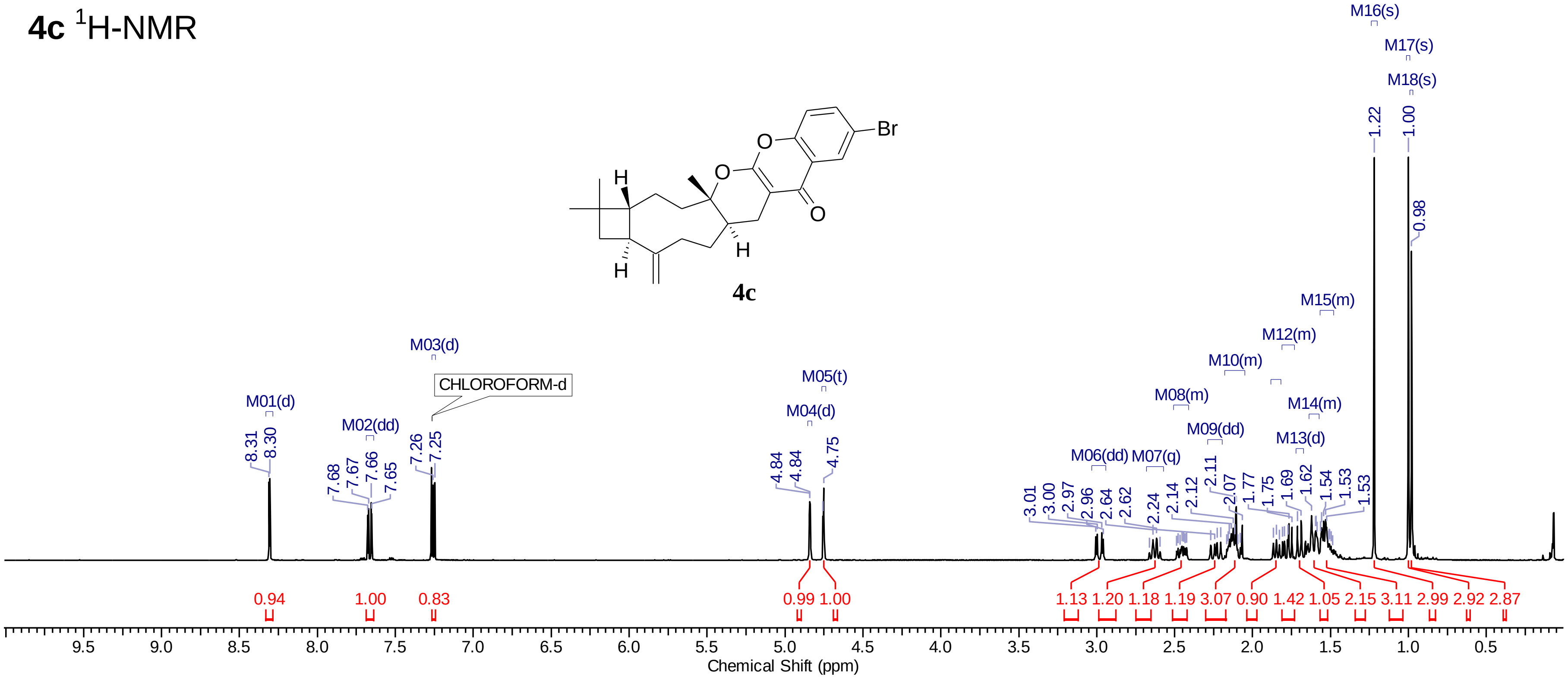
4c 1H-NMR
Br
4c
CHLOROFORM-d
M01(d)
M02(dd)
M03(d)
M04(d)
M05(t)
M06(dd)
M07(q)
M08(m)
M09(dd)
M10(m)
M12(m)
M13(d)
M14(m)
M15(m)
M16(s)
M17(s)
M18(s)
8.31 8.30
7.68 7.67 7.66 7.65
7.26 7.25
4.84 4.84 4.75
3.01 3.00 2.97 2.96 2.64 2.62 2.24 2.14 2.12 2.11 2.07 1.77 1.75 1.69 1.62 1.54 1.53 1.53
1.22 1.00 0.98
0.94 1.00 0.83
0.99 1.00
1.13 1.20 1.18 1.19 3.07 0.90 1.42 1.05 2.15 3.11 2.99 2.92 2.87
9.5 9.0 8.5 8.0 7.5 7.0 6.5 6.0 5.5 5.0 4.5 4.0 3.5 3.0 2.5 2.0 1.5 1.0 0.5
Chemical Shift (ppm)

DEPT135 and ^{13}C NMR

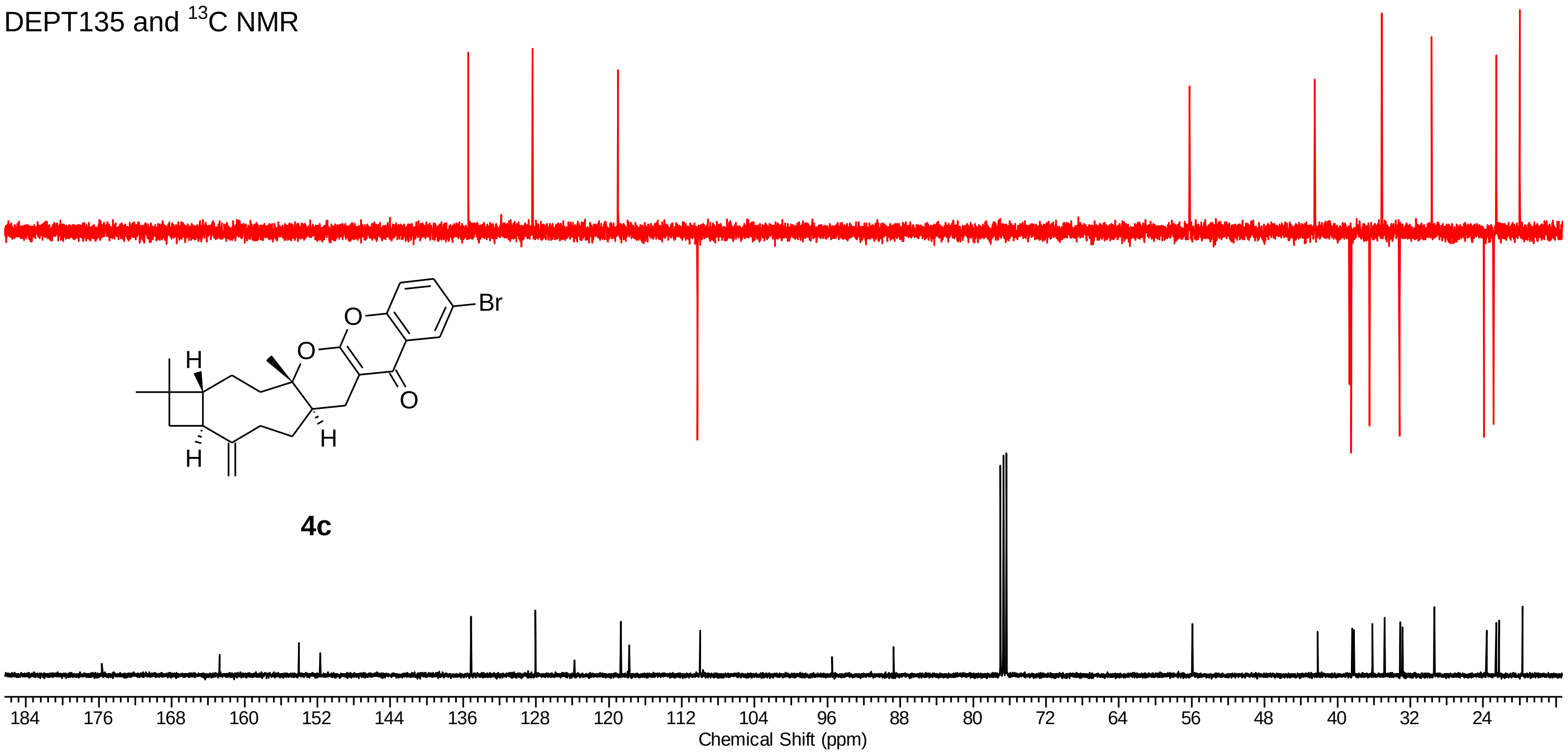

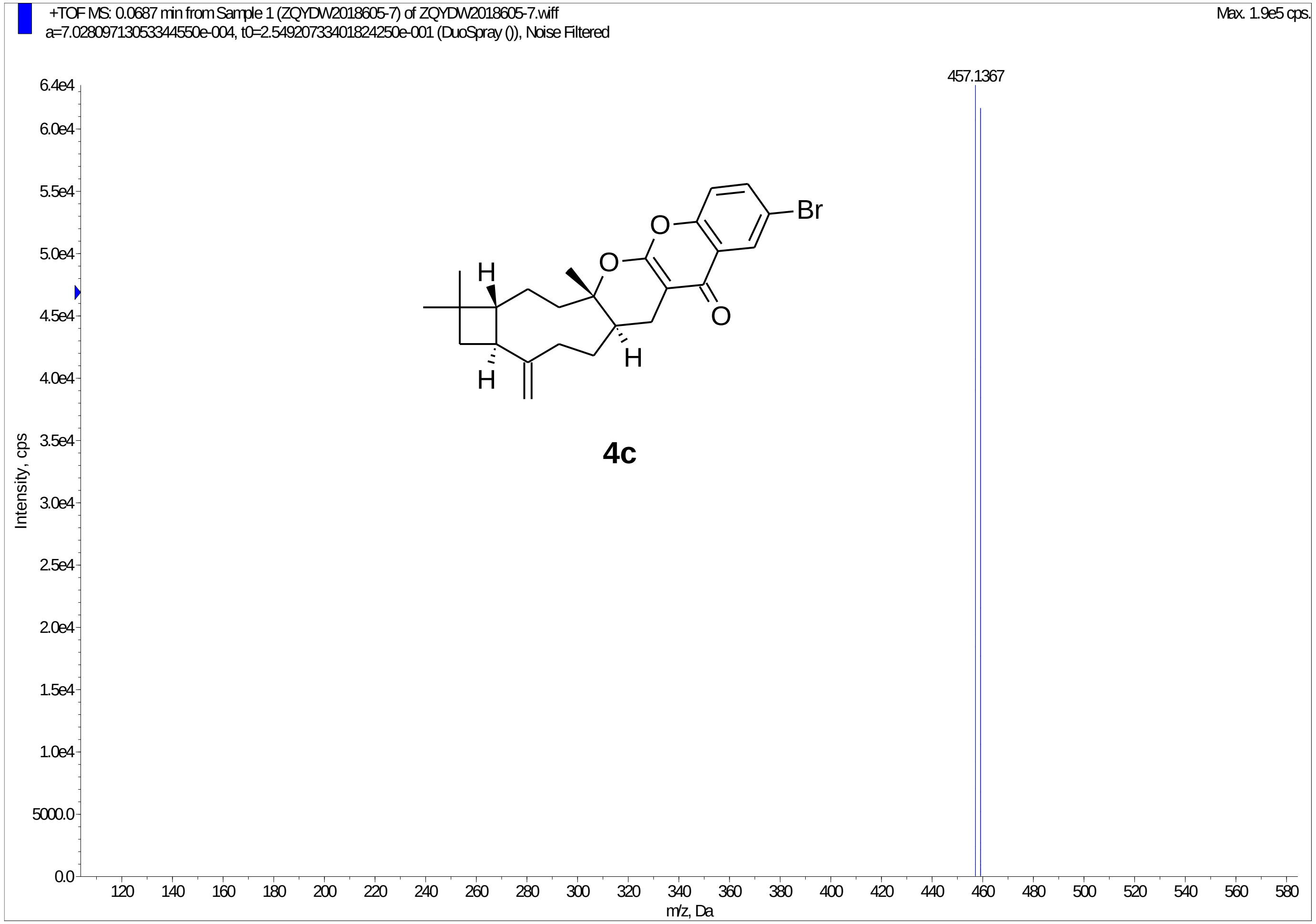
+TOF MS: 0.0687 min from Sample 1 (ZQYDW2018605-7) of ZQYDW2018605-7.wiff
a=7.02809713053344550e-004, t0=2.54920733401824250e-001 (DuoSpray ()), Noise Filtered
Max. 1.9e5 cps.
457.1367
Intensity, cps
m/z, Da
Br
O
H
4c

Compound 5c

5c ^{1}H-NMR

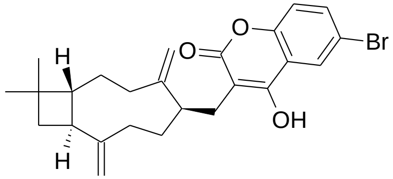

5c

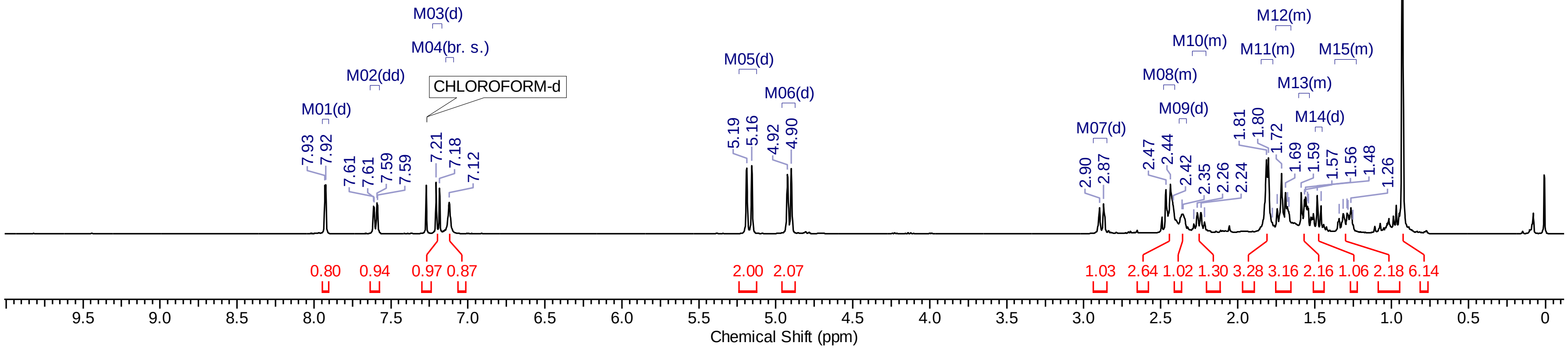

DEPT135 and ^{13}C NMR

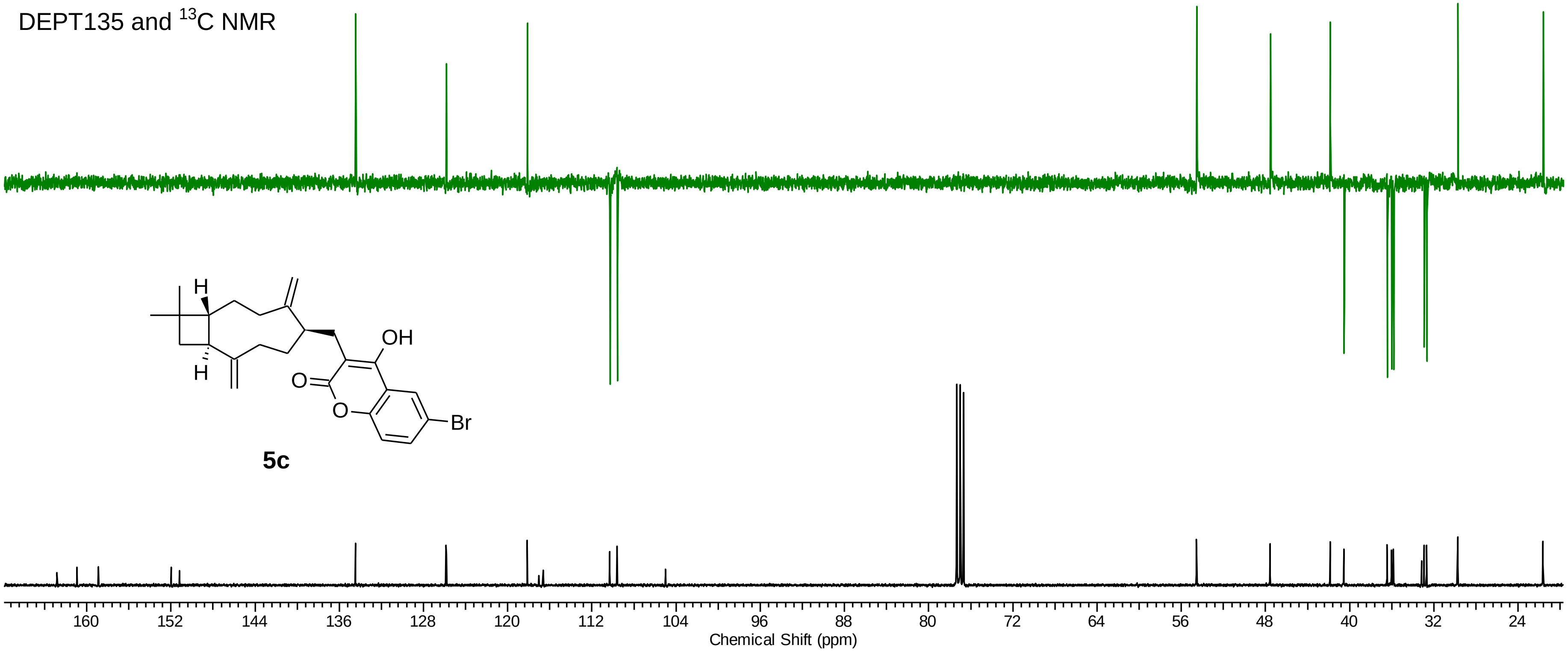

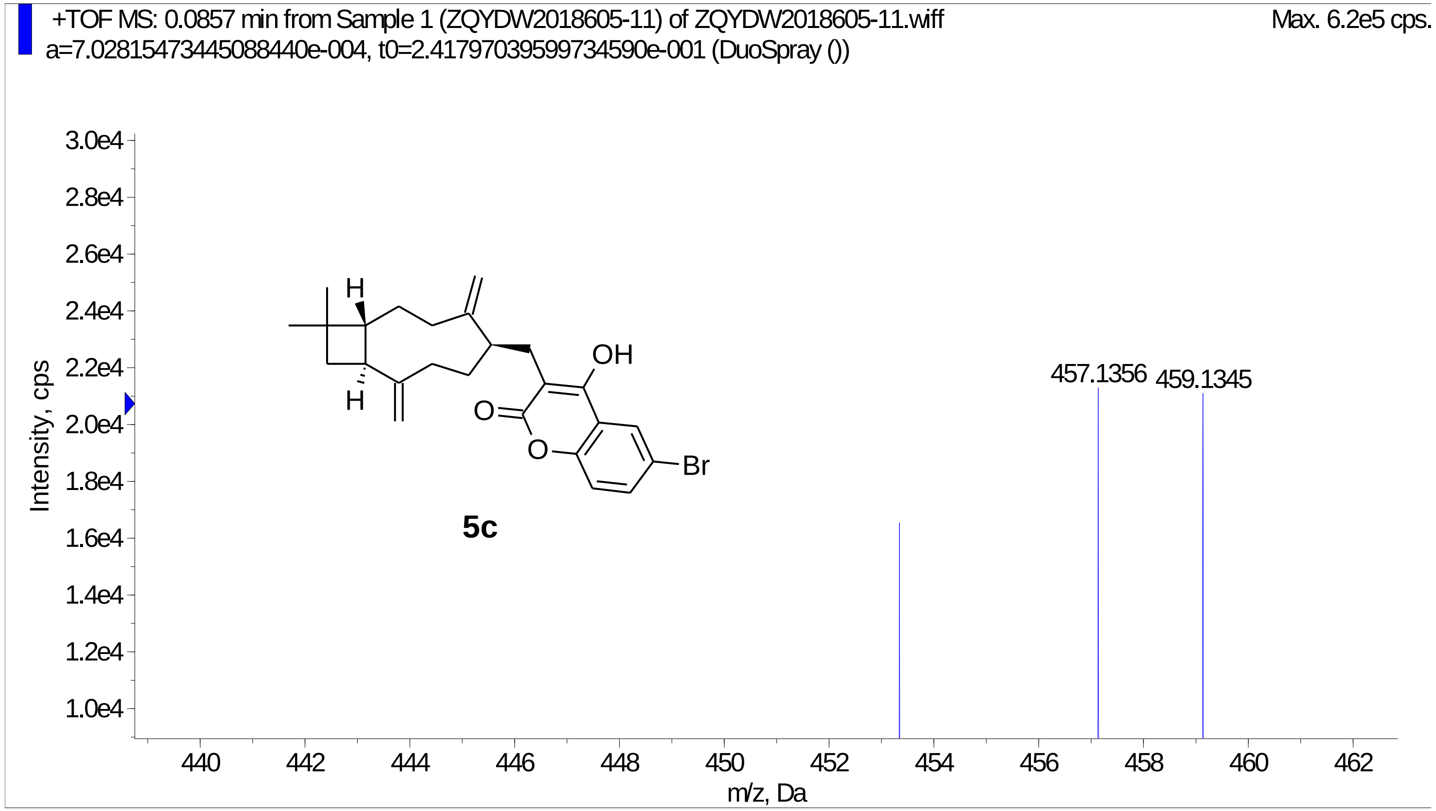
+TOF MS: 0.0857 min from Sample 1 (ZQYDW2018605-11) of ZQYDW2018605-11.wiff
a=7.02815473445088440e-004, t0=2.41797039599734590e-001 (DuoSpray ())
Max. 6.2e5 cps.
Intensity, cps
m/z, Da
457.1356
459.1345
OH
O
O
Br
H
H
5c

Compound 6

6 ^{1}H-NMR

MSJ 96-2 H.ESP

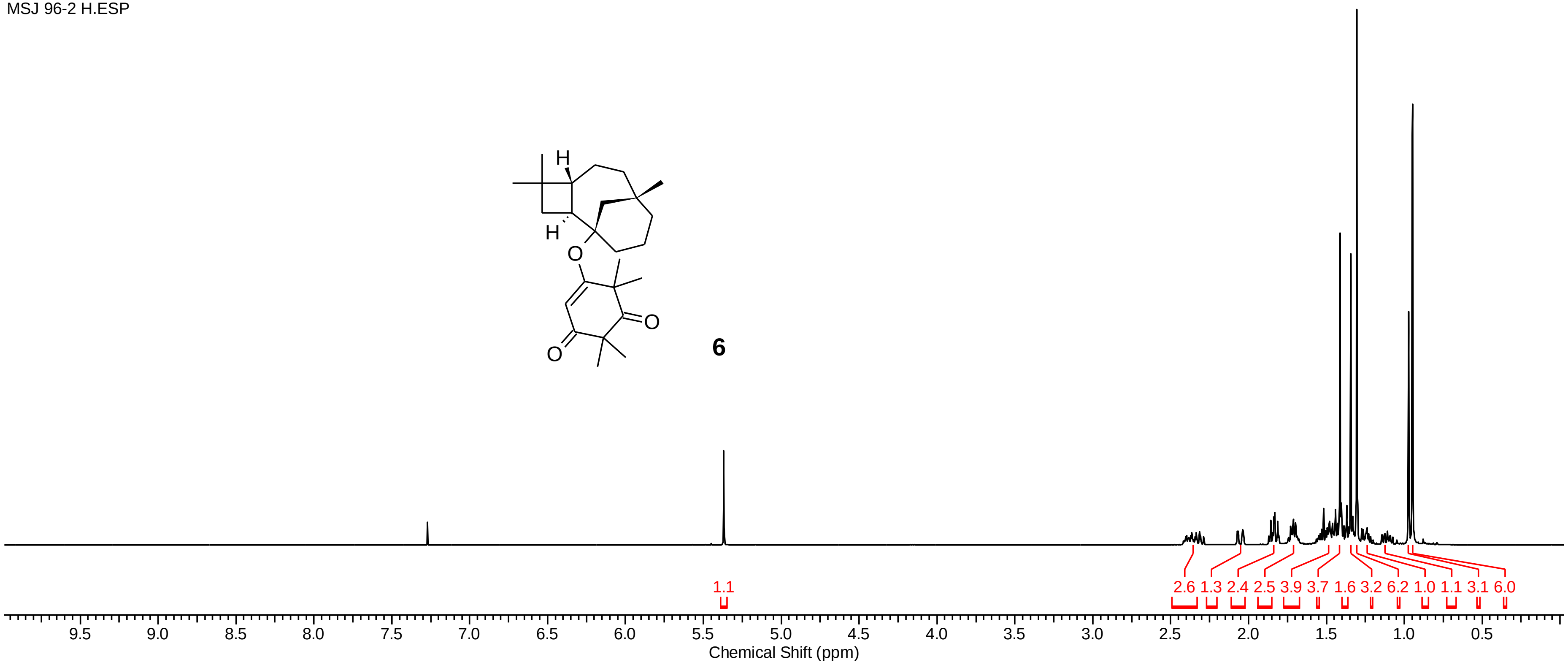

DEPT and ^{13}CNMR

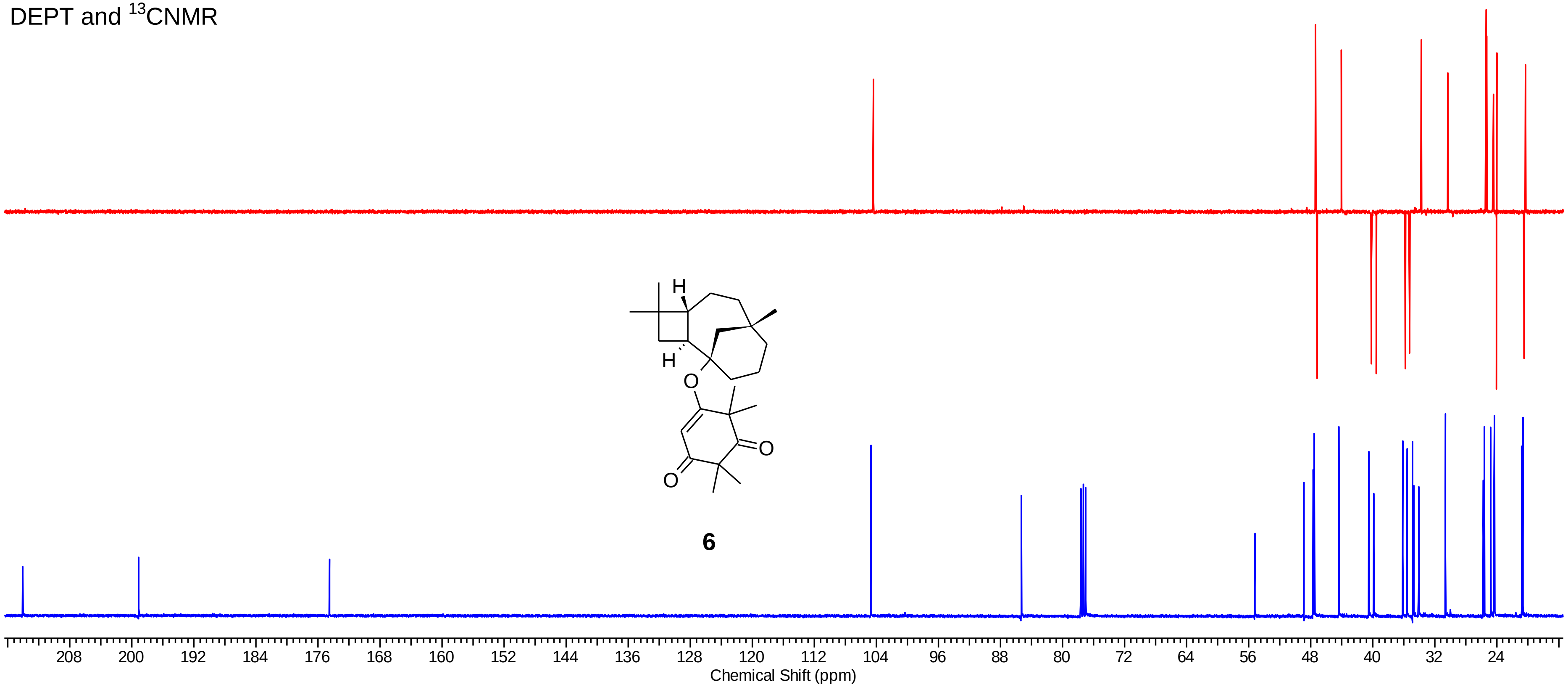

HSQC

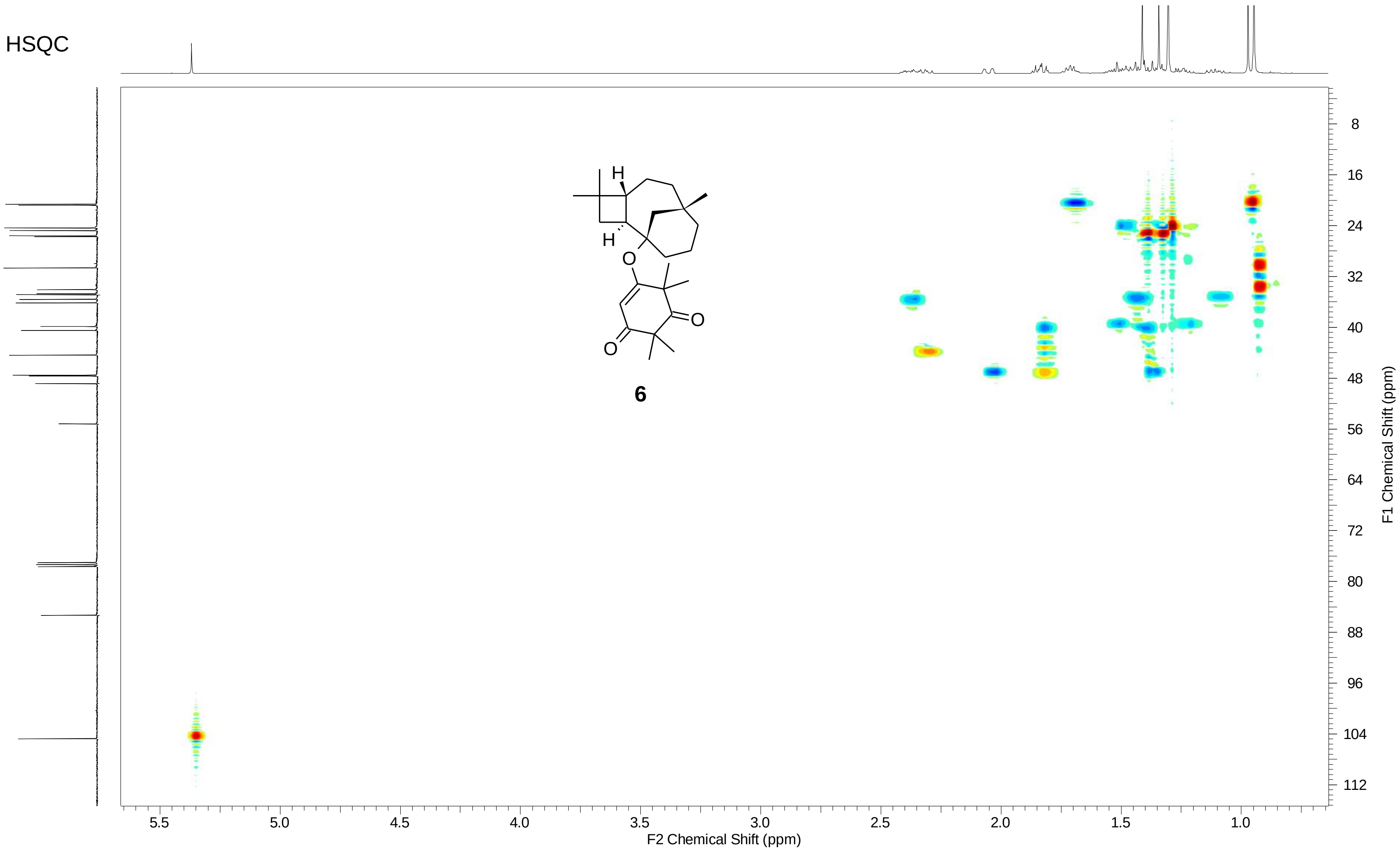

COSY

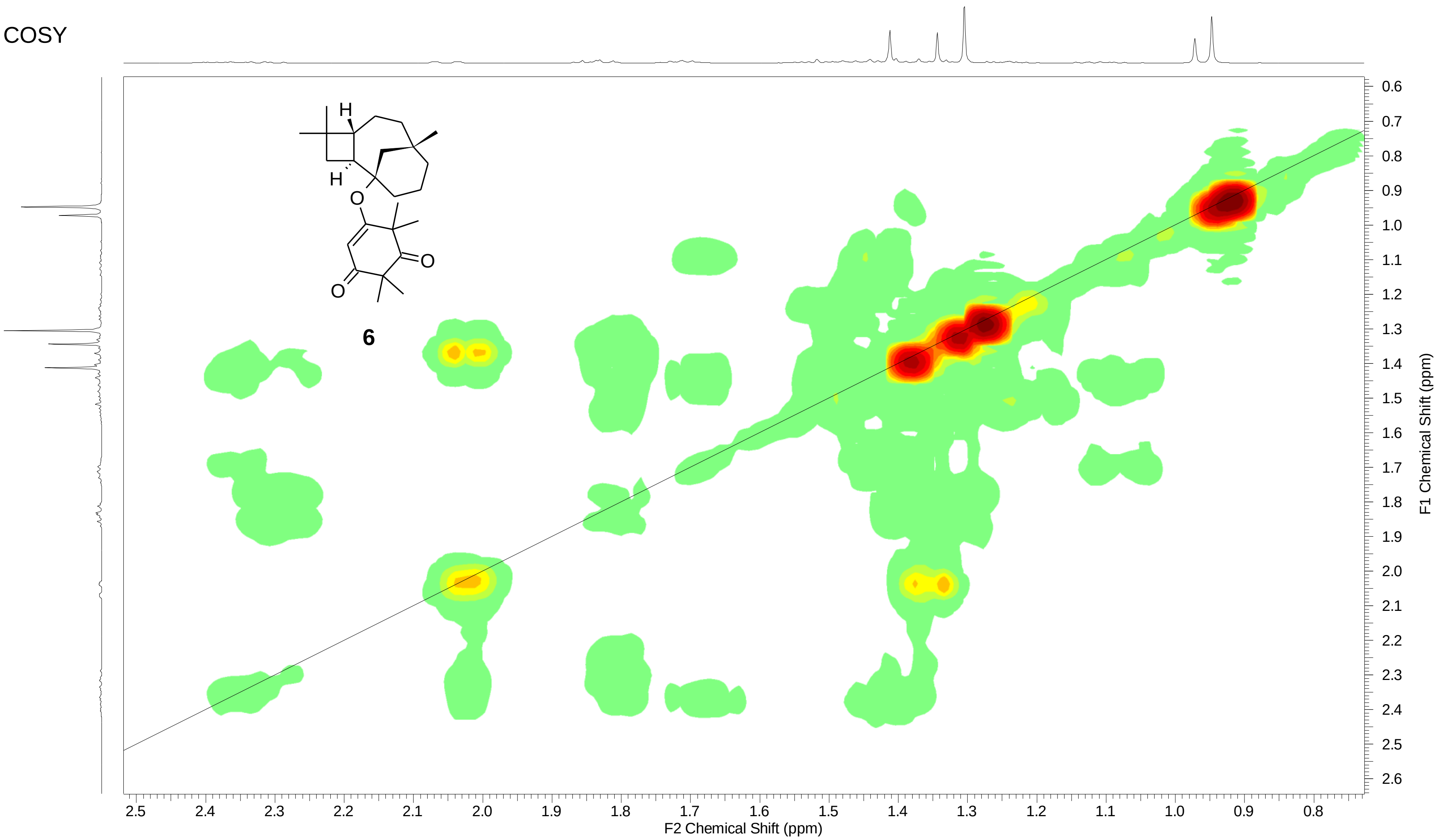
H
H
O
O
O
6
F2 Chemical Shift (ppm)
F1 Chemical Shift (ppm)
2.5
2.4
2.3
2.2
2.1
2.0
1.9
1.8
1.7
1.6
1.5
1.4
1.3
1.2
1.1
1.0
0.9
0.8
0.6
0.7
0.8
0.9
1.0
1.1
1.2
1.3
1.4
1.5
1.6
1.7
1.8
1.9
2.0
2.1
2.2
2.3
2.4
2.5
2.6

HMBC

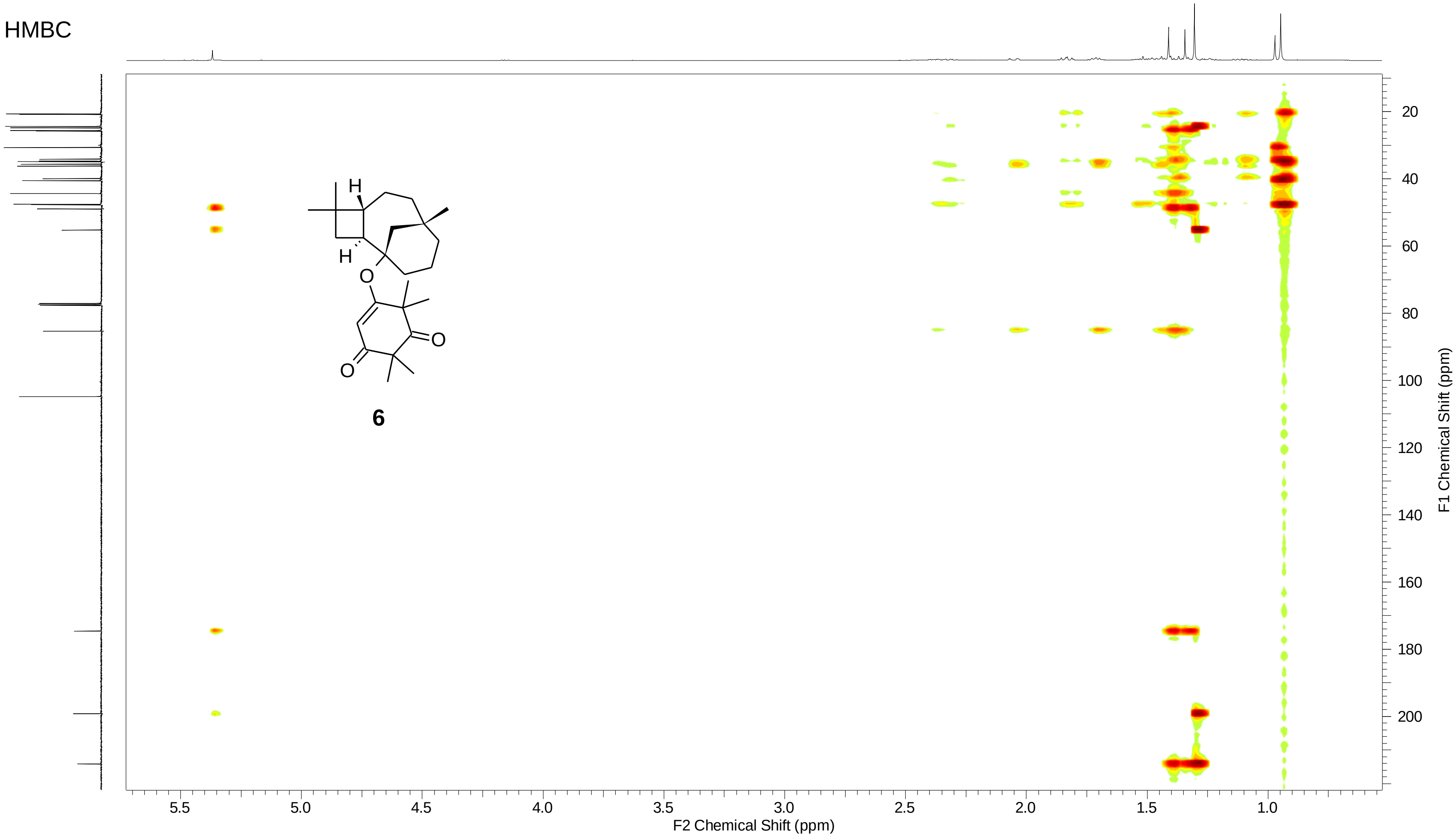

H
H
O
O
O
6
F2 Chemical Shift (ppm)
F1 Chemical Shift (ppm)
5.5
5.0
4.5
4.0
3.5
3.0
2.5
2.0
1.5
1.0
20
40
60
80
100
120
140
160
180
200

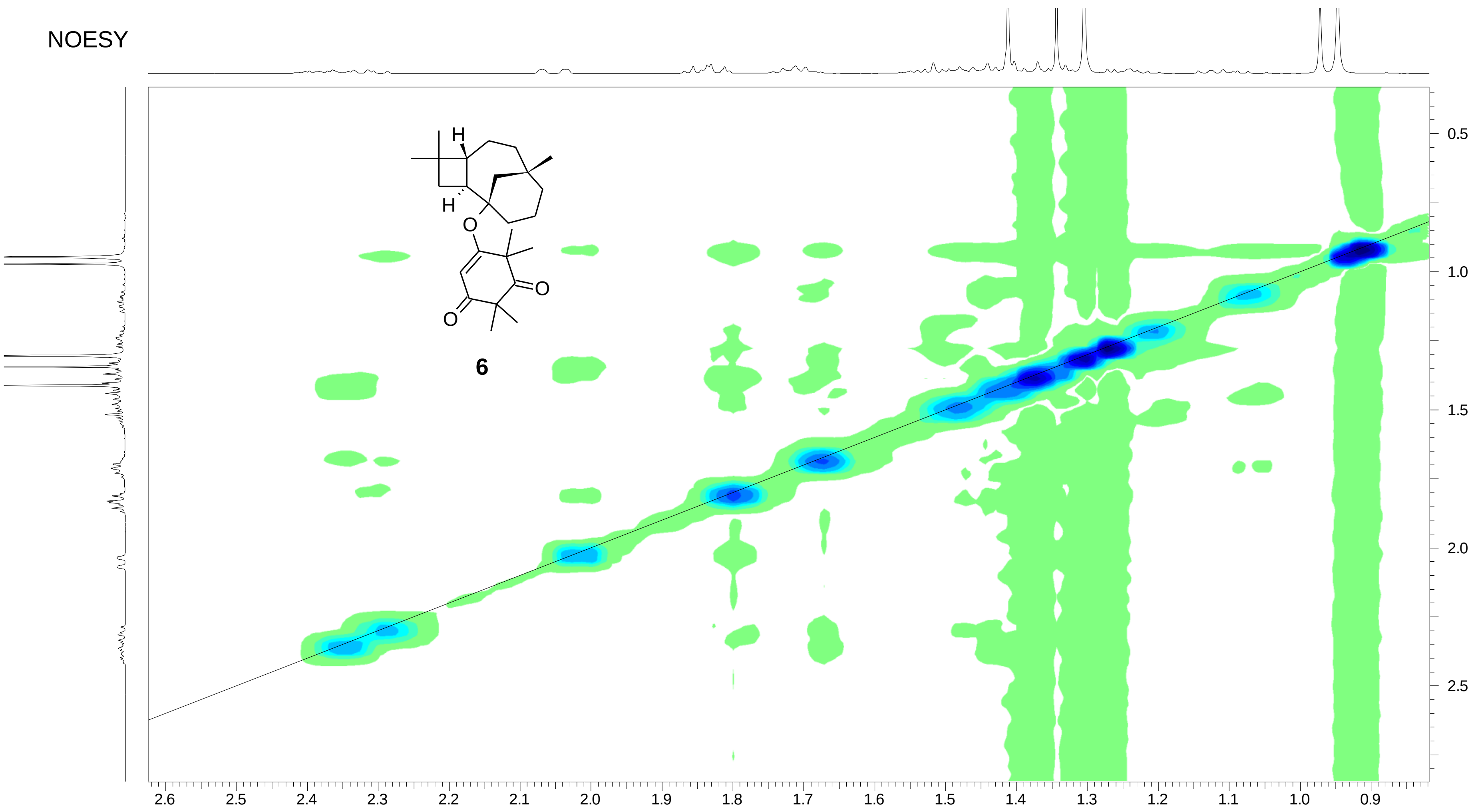
NOESY
H
H
O
O
O
6
2.6
2.5
2.4
2.3
2.2
2.1
2.0
1.9
1.8
1.7
1.6
1.5
1.4
1.3
1.2
1.1
1.0
0.9
0.5
1.0
1.5
2.0
2.5

HR-ESI-MS

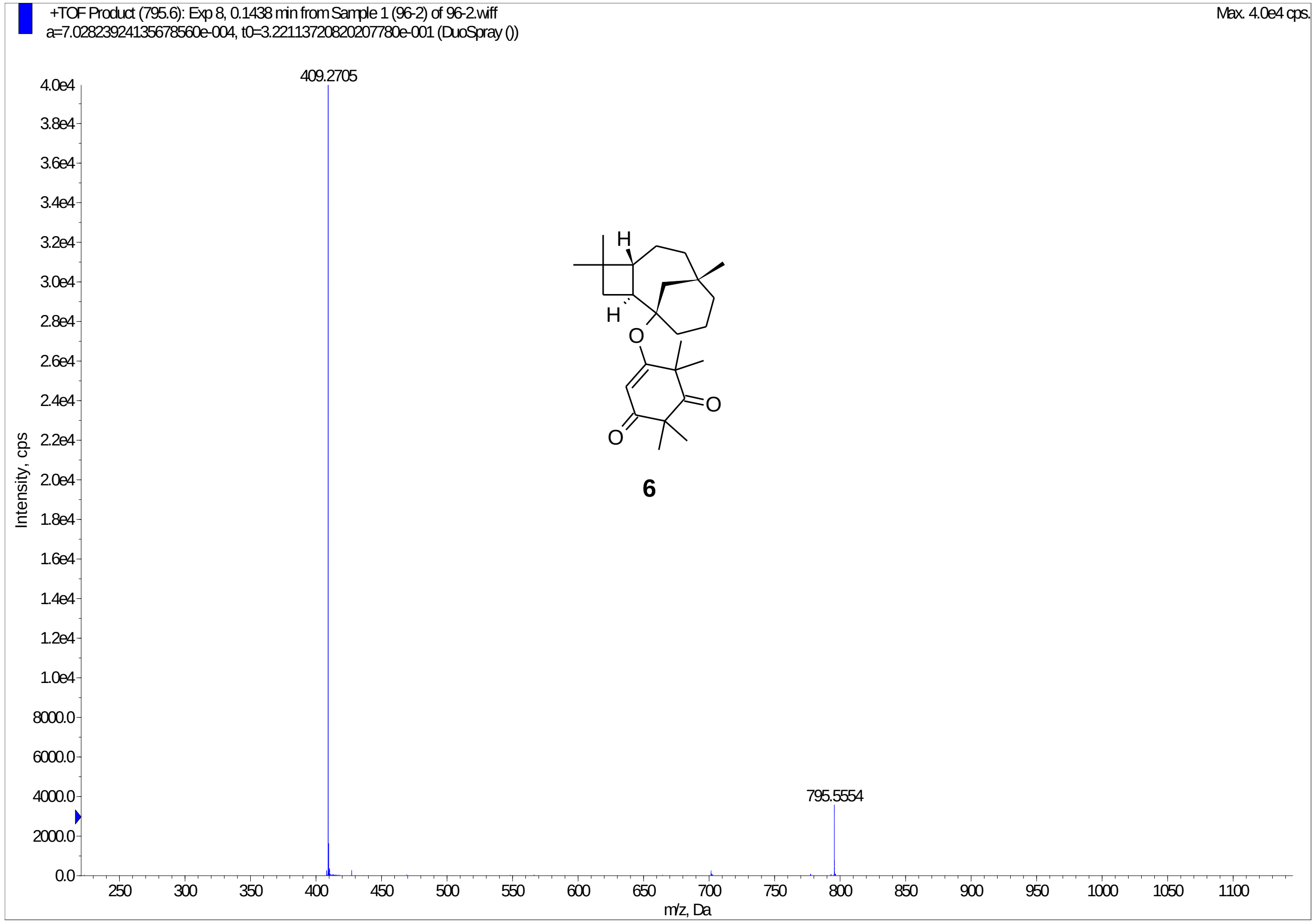

+TOF Product (795.6): Exp 8, 0.1438 min from Sample 1 (96-2) of 96-2.wiff
a=7.02823924135678560e-004, t0=3.22113720820207780e-001 (DuoSpray ())
Max. 4.0e4 cps.
409.2705
795.5554
Intensity, cps
m/z, Da
6

Compound 7

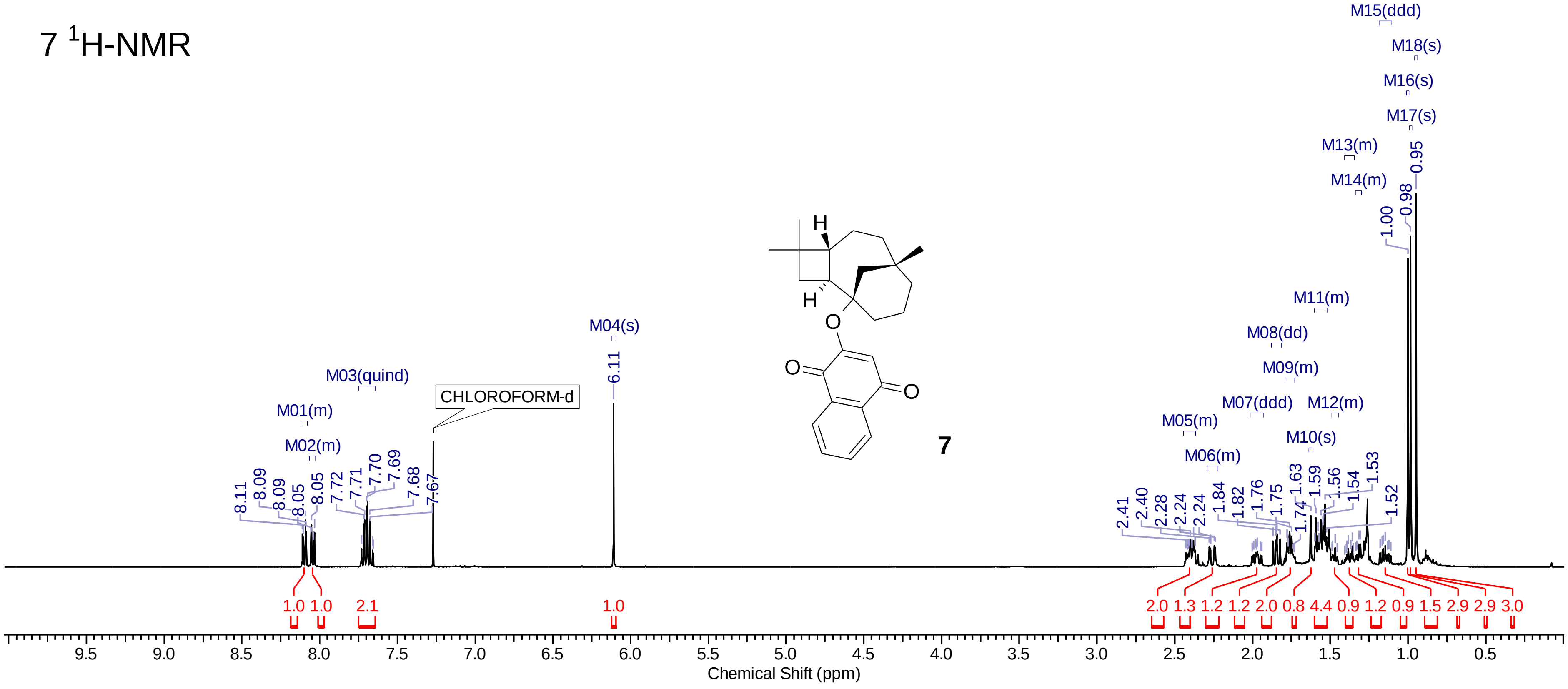

^{13}C-NMR and DEPT135

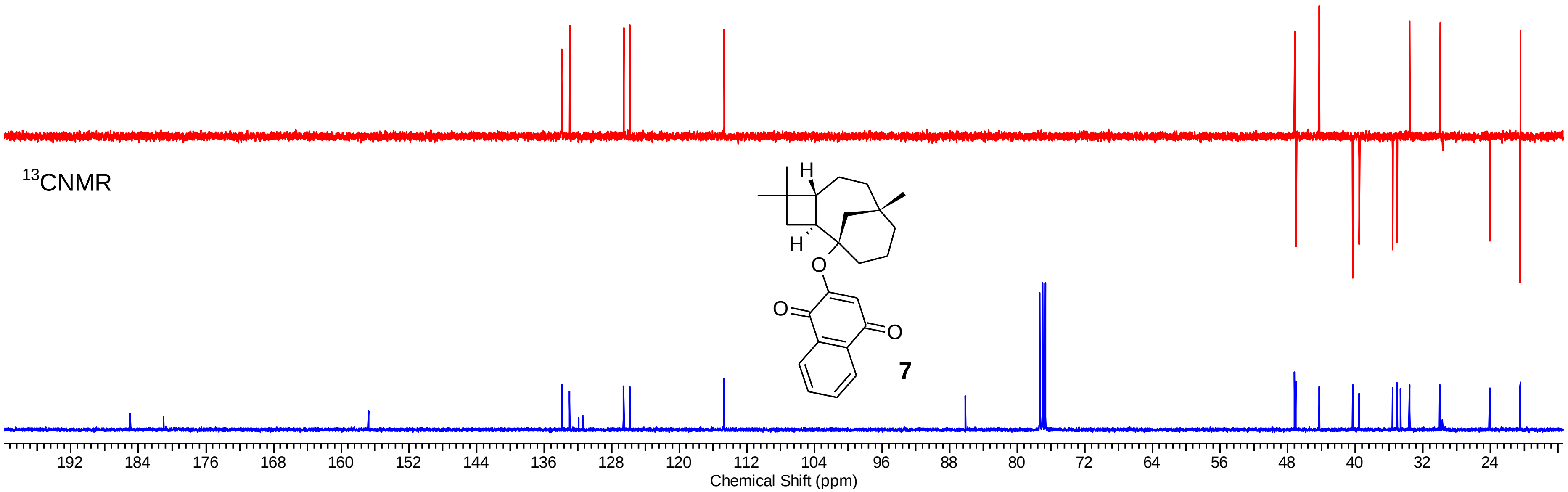

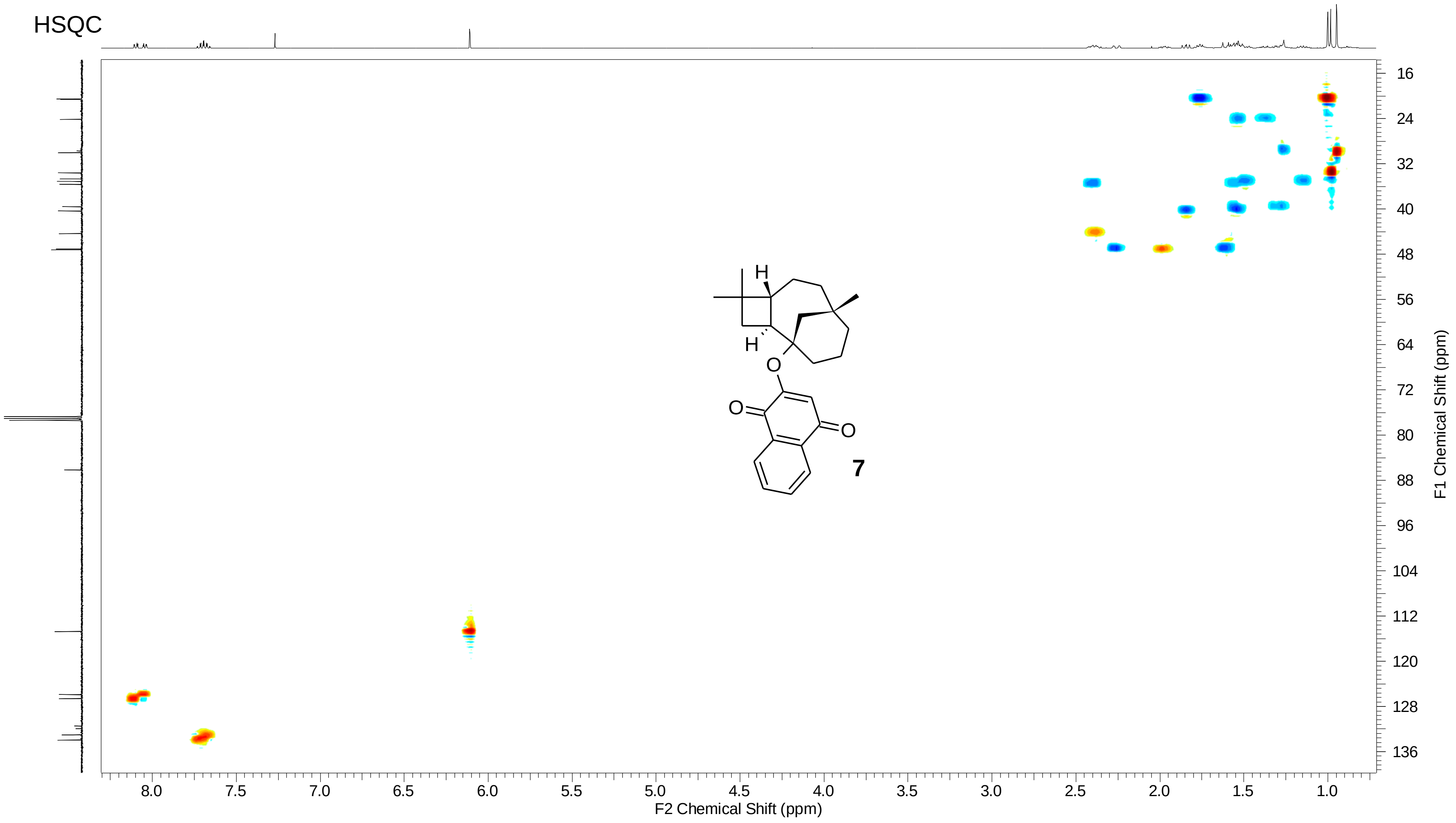
HSQC
16
24
32
40
48
56
64
72
80
88
96
104
112
120
128
136
F1 Chemical Shift (ppm)
8.0
7.5
7.0
6.5
6.0
5.5
5.0
4.5
4.0
3.5
3.0
2.5
2.0
1.5
1.0
F2 Chemical Shift (ppm)
H
H
O
O
O
7

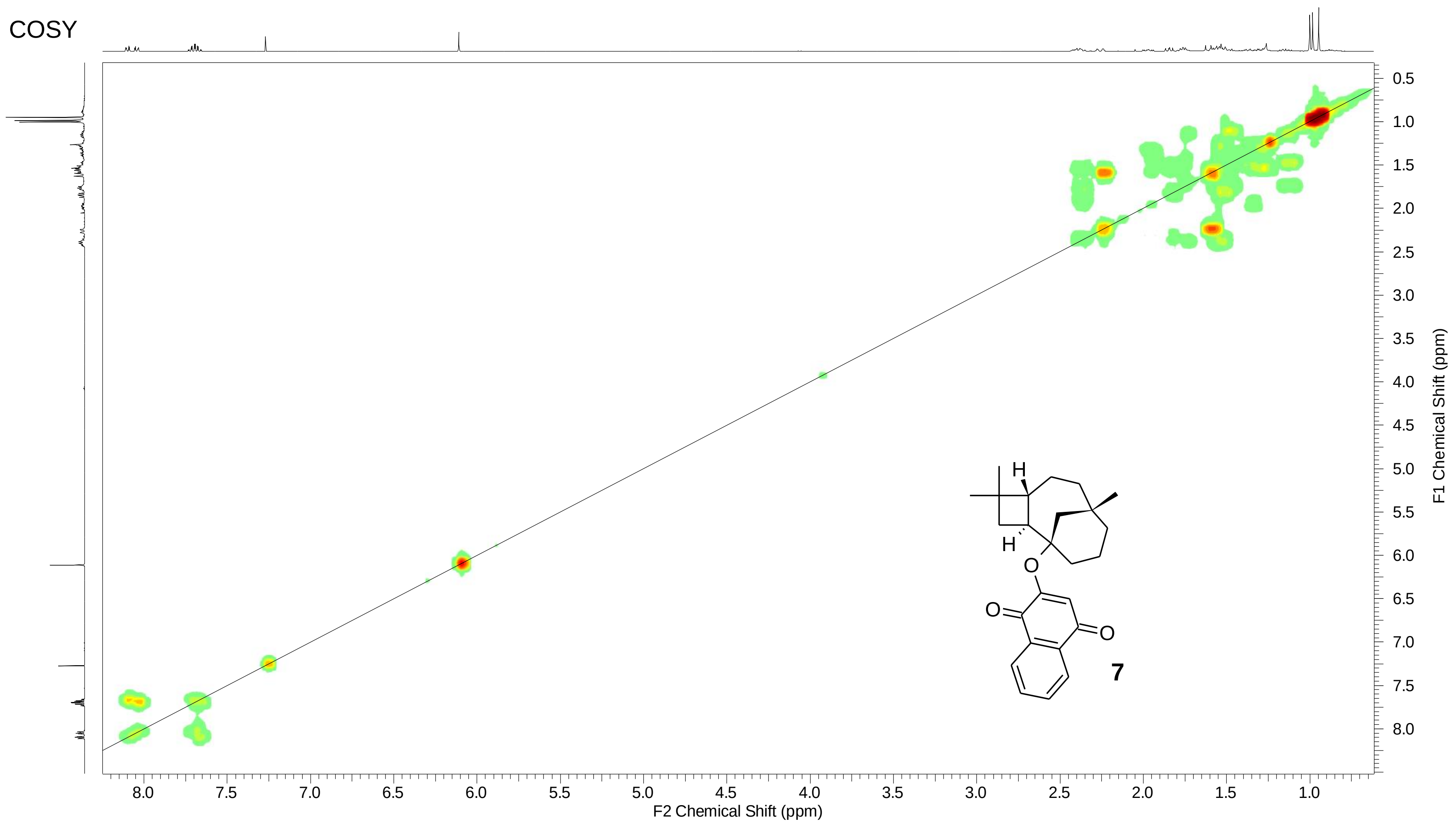
COSY
F2 Chemical Shift (ppm)
F1 Chemical Shift (ppm)
H
H
O
O
O
7

HMBC

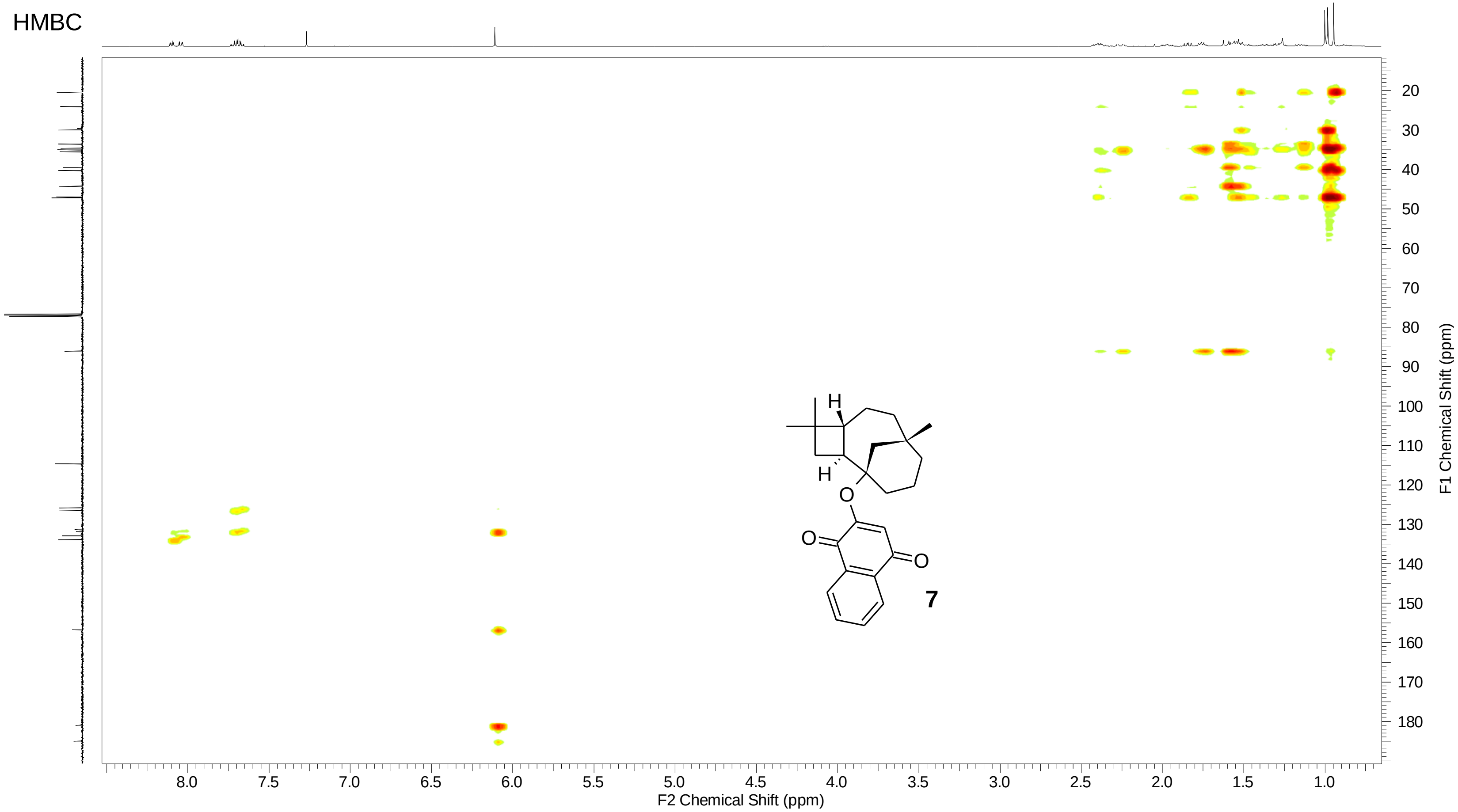

NOESY

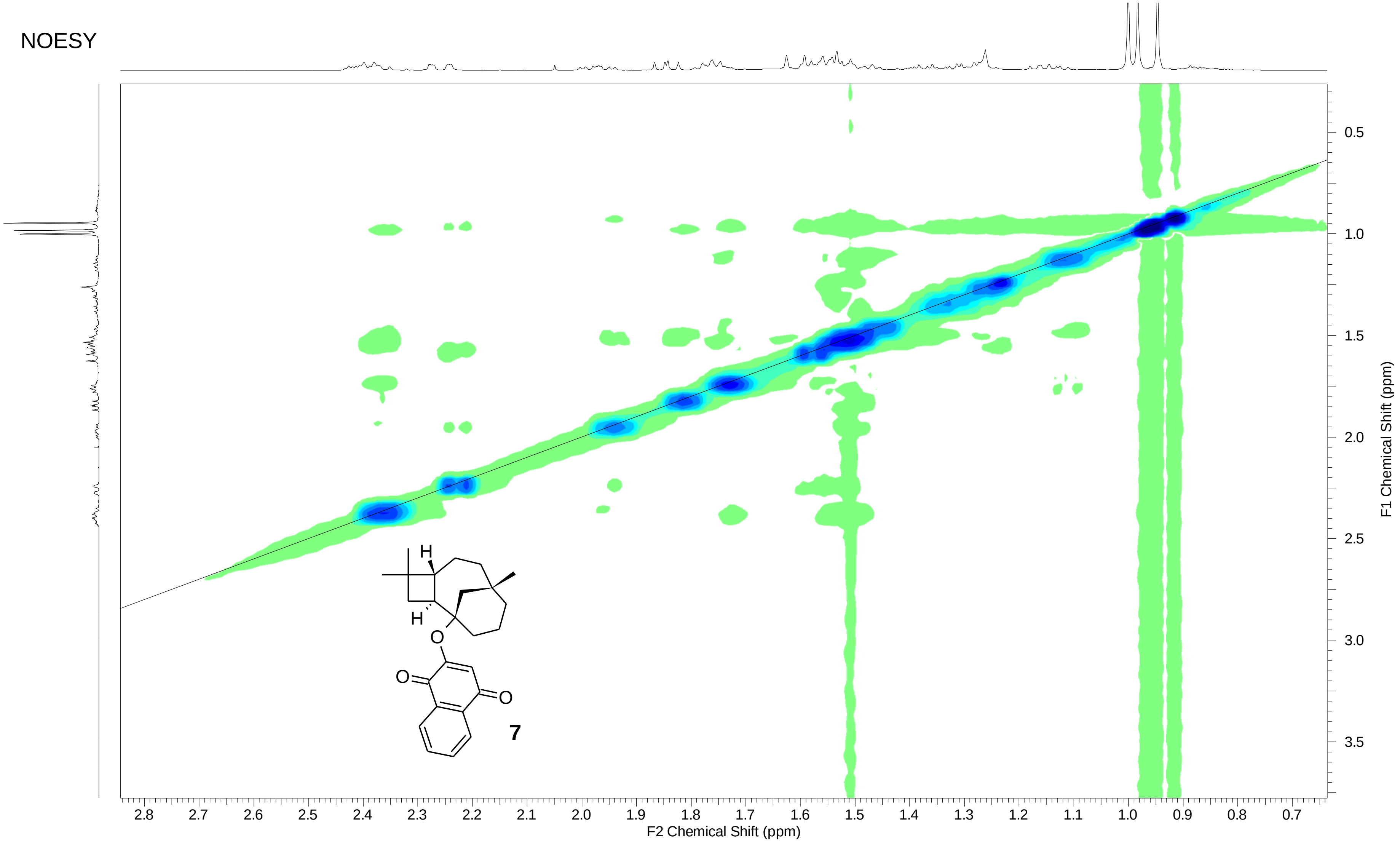

H
H
O
O
O
7
F2 Chemical Shift (ppm)
F1 Chemical Shift (ppm)
2.8
2.7
2.6
2.5
2.4
2.3
2.2
2.1
2.0
1.9
1.8
1.7
1.6
1.5
1.4
1.3
1.2
1.1
1.0
0.9
0.8
0.7
0.5
1.0
1.5
2.0
2.5
3.0
3.5

HR-ESI-MS

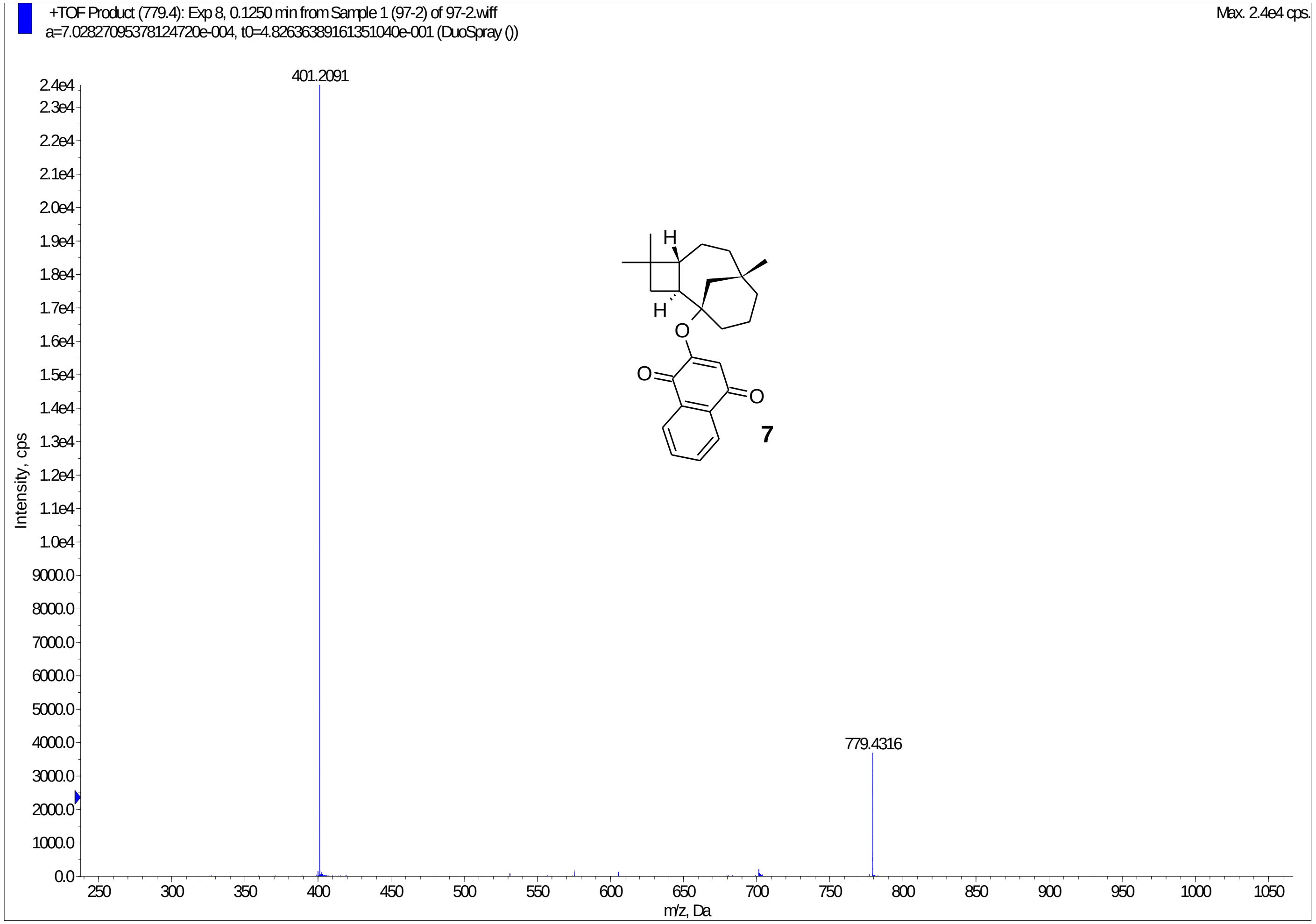

+TOF Product (779.4): Exp 8, 0.1250 min from Sample 1 (97-2) of 97-2.wiff
a=7.02827095378124720e-004, t0=4.82636389161351040e-001 (DuoSpray ())
Max. 2.4e4 cps.
401.2091
779.4316
Intensity, cps
m/z, Da
H
H
O
O
O
7
